# A pangenome analysis pipeline provides insights into functional gene identification in rice

**DOI:** 10.1186/s13059-023-02861-9

**Published:** 2023-01-26

**Authors:** Jian Wang, Wu Yang, Shaohong Zhang, Haifei Hu, Yuxuan Yuan, Jingfang Dong, Luo Chen, Yamei Ma, Tifeng Yang, Lian Zhou, Jiansong Chen, Bin Liu, Chengdao Li, David Edwards, Junliang Zhao

**Affiliations:** 1 Rice Research Institute & Guangdong Key Laboratory of New Technology in Rice Breeding & Guangdong Rice Engineering Laboratory, Guangdong Academy of Agricultural Sciences, Guangzhou, 510640 China; 2 Western Crop Genetics Alliance, Murdoch University, Murdoch, Western Australia 6150 Australia; 3 School of Life Sciences and State Key Laboratory of Agrobiotechnology, The Chinese University of Hong Kong, Hong Kong, SAR China; 4 School of Biological Sciences and Centre for Applied Bioinformatics, University of Western Australia, Perth, WA Australia

**Keywords:** Pangenome, Presence/absence variation, Genomic diversity, PAV-based GWAS

## Abstract

**Background:**

A pangenome aims to capture the complete genetic diversity within a species and reduce bias in genetic analysis inherent in using a single reference genome. However, the current linear format of most plant pangenomes limits the presentation of position information for novel sequences. Graph pangenomes have been developed to overcome this limitation. However, bioinformatics analysis tools for graph format genomes are lacking.

**Results:**

To overcome this problem, we develop a novel strategy for pangenome construction and a downstream pangenome analysis pipeline (PSVCP) that captures genetic variants’ position information while maintaining a linearized layout. Using PSVCP, we construct a high-quality rice pangenome using 12 representative rice genomes and analyze an international rice panel with 413 diverse accessions using the pangenome as the reference. We show that PSVCP successfully identifies causal structural variations for rice grain weight and plant height. Our results provide insights into rice population structure and genomic diversity. We characterize a new locus (*qPH8-1*) associated with plant height on chromosome 8 undetected by the SNP-based genome-wide association study (GWAS).

**Conclusions:**

Our results demonstrate that the pangenome constructed by our pipeline combined with a presence and absence variation-based GWAS can provide additional power for genomic and genetic analysis. The pangenome constructed in this study and the associated genome sequence and genetic variants data provide valuable genomic resources for rice genomics research and improvement in future.

**Supplementary Information:**

The online version contains supplementary material available at 10.1186/s13059-023-02861-9.

## Background

Rice (*Oryza sativa* L*.*) is one of the most important staple crops, feeding nearly half of the world’s population. As the population expands to 10 billion people [1, 2], there is an urgent need to increase the productivity of crops, while facing the impact of climate change on agricultural productivity. The application of genomics-assisted breeding is believed as one of the best opportunities to increase crop productivity, with the exploitation of diversity stored in germplasm collections as a major resource for crop improvement [3, 4]. With rapid advances in DNA sequencing technologies, genomic diversity within rice germplasm has been characterized by resequencing thousands of individuals and comparing the resulting data with reference genome assemblies. However, it is now understood that a single reference genome does not represent the genomic diversity of a species due to significant structure variations (SVs) between individuals [5]. To capture the genomic variations in a population, pangenome assemblies have been constructed. Pangenomes represent the gene contents of a species rather than a single individual [6], and using a pangenome as a reference, SVs can be more easily and accurately genotyped by low-cost short-read sequencing technologies, facilitating efficient characterization and capture of genomic diversity within a species.

Pangenomes have now been constructed and analyzed for several crop species, including wheat, Brassicas, barley, banana, and pigeon pea [7–11]. Several pangenomes have been constructed in rice, and pangenomic analyses have identified genome sequences absent in the Nipponbare reference, the most commonly used reference in rice genomic studies [12–14]. For example, a pangenome study using 3010 rice accessions identified 268 Mb of new sequences, with 12,465 new genes and 19,721 dispensable genes compared to the Nipponbare rice reference genome [15].

Recent advances in pangenomics have led to the construction of graph-based pangenomes [16, 17] that code genetic variants as nodes and edges and preserve the contiguity of the sequence and structural variation between individuals [18]. Graph-based pangenome approaches are relatively new, but have been applied to many important crops, including soybean, bread wheat, and rice [13, 19–21]. Recently, a comprehensive graph-based rice pangenome was constructed with 251 accessions spanning both cultivated and wild species of Asian and African rice, which helps characterize lineage-specific haplotypes for agronomic-related genes and shed light on rice evolutionary events [22]. Despite its advantages, the graph-based pangenome also has some limitations; for example, as most genome analysis tools were developed for linear sequences, scalable software and mature data structures designed explicitly for graph-based pangenome analysis are still limited at present. A linear format pangenome with a fixed order coordinate system is still valuable for genomic studies. However, linear pangenomes struggle to represent the position of SVs, resulting in the loss of valuable information.

In this study, we developed a pangenome construction strategy that can preserve the position information of novel sequences identified during pangenome construction and embed them into a linear pangenome. We also developed a suite of tools for mapping short-read sequencing data to this pangenome for SV genotyping, including presence and absence variations (PAVs), translocations, and inversions, that can recover the genomic position of sequence variations. We then applied this pipeline to construct a rice pangenome using 12 diverse accessions representing major subpopulations of Asian rice, and identified SVs from an international rice mini core panel consisting of 413 accessions [23]. Our results revealed extensive genomic diversity among rice germplasm, and population genetic analysis based on PAVs identified in the pangenome provided insights into the population structure of Asian rice. The PAVs were further used for a genome-wide association study (GWAS), resulting in the successful identification of causal PAVs that affect grain weight and plant height. This study presents a new tool for pangenome analysis, demonstrating the advantages of using a coordinate-linked linear pangenome to identify PAVs for functional analysis. Our results provide valuable resources for rice functional genomics study.

## Results

### A novel pangenome construction and PAV analysis pipeline

**Fig. 1:**
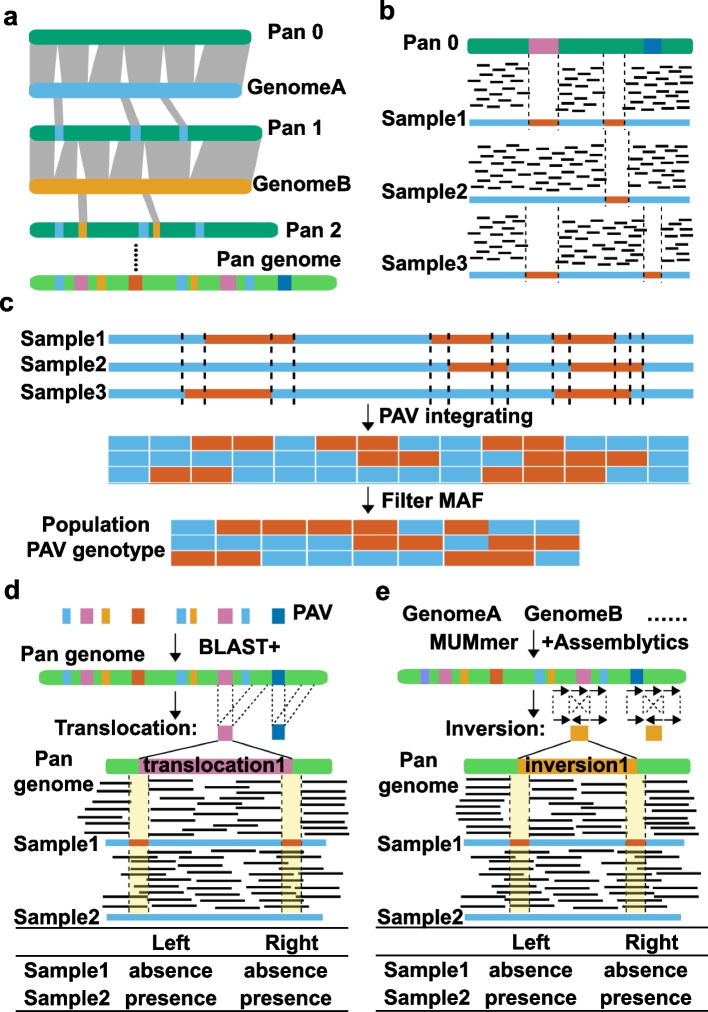
Scheme diagram of PSVCP pipeline. **a** Construction of linearized pangenome. Genomes used for pangenome construction are iteratively aligned between genomes and the starting reference to identify novel segments, then integrate these sequences into the reference genome to construct a pangenome. **b** PAV genotyping in the pangenome for single accession. PAVs are detected by mapping sequencing reads of each accession to the pangenome and calculating mapping coverage using a 20-bp window. Two adjacent 20-bp regions are merged if they show the same presence or absence pattern. **c** Population-wide PAV genotyping. The breakpoint positions of each accession are recorded, and a complete set of all breakpoint positions (the breakpoints’ union) are generated. Every segment with two adjacent breakpoints from the breakpoints’ union is defined as a new PAV region, named by the adjacent left breakpoint position. PAVs in each accession are re-called using the integrated PAV genotypes. All accessions’ new PAV genotypes are combined by row, generating a PAV genotype matrix with accession names as row labels and the adjacent left breakpoint position as column labels. The PAV genotype matrix was further filtered by minor allele frequency (MAF) >0.05. **d** Translocation genotyping by PSVCP. The novel insertions are aligned against the pangenome using BLAST+. The novel insertions matched to 2 or more sequences on the pangenome are identified as potential translocations. The presence or absence of translocations is determined by looking at the read mapping around its breakpoints, spanning a 39-bp region with a conjunction point at the center. **e** Inversions genotyping by PSVCP. The inversions are identified by comparing each genome used for pangenome construction with the pangenome using assemblytics. We further genotyped each potential inversion on the pangenome from 413 accessions by examining the mapping coverage of the 39-bp region with breakpoints in the center

In this study, we developed a pangenome construction and SV genotype calling pipeline (PSVCP) (Additional file 1: Fig. S1). The pipeline includes three main steps, (1) iterative alignment between genomes to identify novel segments, then integrating these sequences into the reference genome to construct a pangenome (Fig. 1a). (2) Mapping short-read sequencing data to the pangenome to detect PAVs based on read coverage (Fig. 1b). (3) Calling SVs (including PAVs, translocations and inversions) based on the pangenome. The PAVs are identified by investigating short-read mapping and population-level comparison (Fig. 1c). Potential translocations are first identified by aligning the novel PAV sequences to the reference genome (Fig. 1d). Potential inversions are identified by comparing genomes used for pangenome construction with the pangenome (Fig. 1e). Finally, translocations and inversions are genotyped in individual accession by surveying the mapping coverage of the breakpoints of inversions and translocations (Fig. 1d, e).

**Fig. 2:**
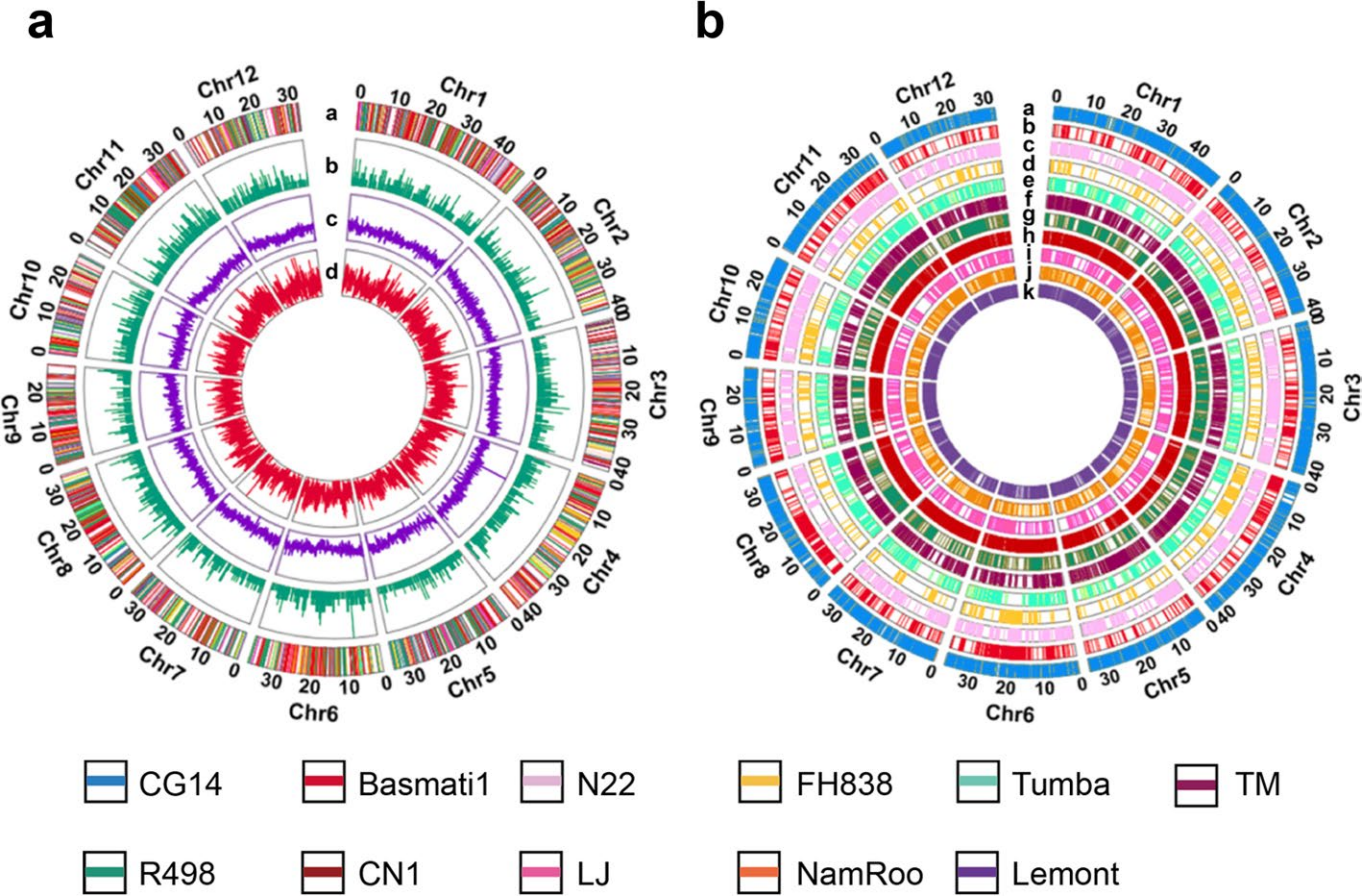
Feature of the rice pangenome constructed by PSVCP and distributions of PAVs in the other 11 rice genomes. **a** Feature of the rice pangenome. From outer-most track to innermost track: (a) New genes inserted in the Nipponbare reference (MSU7.0) from PAVs. The different colors indicate the origin of the PAVs; (b) Density of genes in the Nipponbare reference genome interrupted by PAVs; (c) Gene density; (d) PAV density; **b** PAV distributions of the 11 genomes. Genomes from outer-most track to innermost track: (a) CG14; (b) Basmati1; (c) N22; (d) FH838; (e) Tumba; (f) TM; (g) R498; (h) CN1; (i) LJ; (j) NamRoo; (k) Lemont

We initially used 12 de novo assembled genome sequences of cultivated rice, including 11 Asian cultivated rice (*Oryza sativa*) accessions selected from 33 representative accessions based on their subpopulation [13] and one African cultivated rice (*Oryza glaberrima*) (Additional file 2: Table S1) for pangenome construction using Nipponbare as the primary reference [24]. By whole genome comparisons we identified 24,585 novel sequences that were inserted into the Nipponbare reference, with 1250 (out of 24,585) sequences further classified as potential translocations and 3326 inversions. The mean, median, maximum, and sum of novel sequence lengths are 2607 bp, 338 bp, 96,797 bp, and 64.10 Mbp respectively (Additional file 1: Fig. S2a, b). A subset of these sequences was validated by PCR amplification and sequencing (Additional file 1: Fig. S3). We analyzed the distribution of these additional sequences and found that 43.1% overlapped ±2 kb upstream/downstream of genes, while 35.7% of the additional PAV sequences overlapped with genic regions (Additional file 1: Fig. S2c). Altogether, 6797 sequences were inserted into 5925 Nipponbare genes (Fig. 2). A total of 1939 new genes were de novo annotated, and functional analysis suggests that they are enriched with terms associated with photosynthesis, the generation of precursor metabolites, and energy (Additional file 2: Table S2). Modelling suggests that the initial 12 rice accessions were sufficient to capture the majority of sequence diversity within rice (Additional file 1: Fig. S4 and S5a).

The completeness of the pangenome was evaluated using Benchmarking Universal Single-Copy Orthologs (BUSCO) [25] (Additional file 2: Table S3). Of the 1614 single-copy orthologs identified in embryophytes, 98.8% were complete in our assembly, which is similar to the Telomere-to-Telomere MH63KL1 genome or a little higher than the 3K rice pangenome (98.0%) [15] (Additional file 2: Table S3). We mapped short-read sequencing data for 413 rice accessions collected from a diverse international panel (RPD2) [23] to the pangenome and the Nipponbare genome respectively. The results showed the average mapping rate to the pangenome was 97.84%, which was higher than the mapping rate to the Nipponbare rice reference (93.05%) (Additional file 1: Fig. S5b). These results demonstrate that our pangenome captured more diversity than the single Nipponbare reference.

### Population-wide TE and PAV analysis in an international diverse rice panel

Illumina whole genome sequencing data were obtained from 413 accessions representing an international rice collection [23]. The reads were mapped to the pangenome, and PAVs, translocations, and inversions were genotyped using the PSVCP pipeline. In addition, we also applied our pipeline using the 3K rice genome sequencing data (>15×) (Additional file 2: Table S4) to generate an additional PAV matrix as a supplementary resource for the rice genomic community, which can be accessed at https://osf.io/38gtp/. The PAVs were validated in randomly selected accessions as described above (Additional file 1: Fig. S3). We validated the potential translocations (Additional file 2: Table S5-7 and Additional file 1: Fig. S6-11) and inversions (Additional file 2: Table S8-11 and Additional file 1: Fig. S12-17) identified in the present study using PacBio long-read sequencing data and de novo genome assemblies (Additional file 3. Supplemental notes). The influence of the breakpoints introduced during pangenome construction on short-read mapping was also evaluated (Additional file 1: Fig. S18-21, and Additional file 2: Table S12-14). The results demonstrated that read coverage changes caused by breakpoints do not affect the PAV genotyping results in our pipeline (Additional file 3. Supplemental notes).

Around 85% of the inserted sequences were transposable elements, with 40% annotated as Gypsy LTR-retrotransposons and 28.6% as Helitron DNA transposons (Additional file 2: Table S15). We examined the diversity of representative retrotransposon families across the 413 accessions [26] and identified 66,441 variable retrotransposon sequences, with 29,281 (44%) absent from the Nipponbare reference assembly. Retrotransposon abundance ranged from 12 (Rn60/Gypsy) to 15,599 copies (Rire3 /Gypsy). Notably, half of the copies in the retrotransposon TE families Rn60, Rire3, Fam81-fam82, Rire2, Hopi, Fam93_ors14, Fam51_osr4, and Tos17 were not identified in the Nipponbare reference. The majority of retrotransposons were from Hopi, Fam81-fam82, and Rire3 TE families, which belong to the Gypsy family, and most of these originate from *Indica* accessions, suggesting an expansion of Gypsy elements in *Indica* compared to *Japonica* [27, 28]. TE families Fam93_ors14, Hopi, and Fam81-fam82 show significantly higher frequency in *Indica* than *Japonica* and *Aus* accessions, while the Rire3 family is less abundant in *Aus* varieties compared to the other populations (Additional file 2: Table S16). This suggests an ongoing transposition during domestication and subsequent breeding.

We further analyzed the minor allele frequency (MAF) of PAVs within genic and intergenic regions. A higher MAF was observed within genic regions (Additional file 1: Fig. S22 and Additional file 2: Table S17), suggesting a lower purifying selection for PAVs in genic regions. It is noteworthy that although our analysis was based on a representative subset of an international diverse panel (the selected 413 accessions), analysis using this subset may not fully display the MAF distribution of all rice germplasms. A total of 11,617 (28.9%) dispensable genes across the 413 rice accessions were identified (Additional file 2: Table S18). Annotation suggests that these genes are enriched for functions associated with protein phosphorylation, telomere maintenance, DNA duplex unwinding, photosynthesis, defense response, and pathogenesis (Additional file 2: Table S19), which is similar to the findings in other crop pangenome studies [29, 30].

**Fig. 3:**
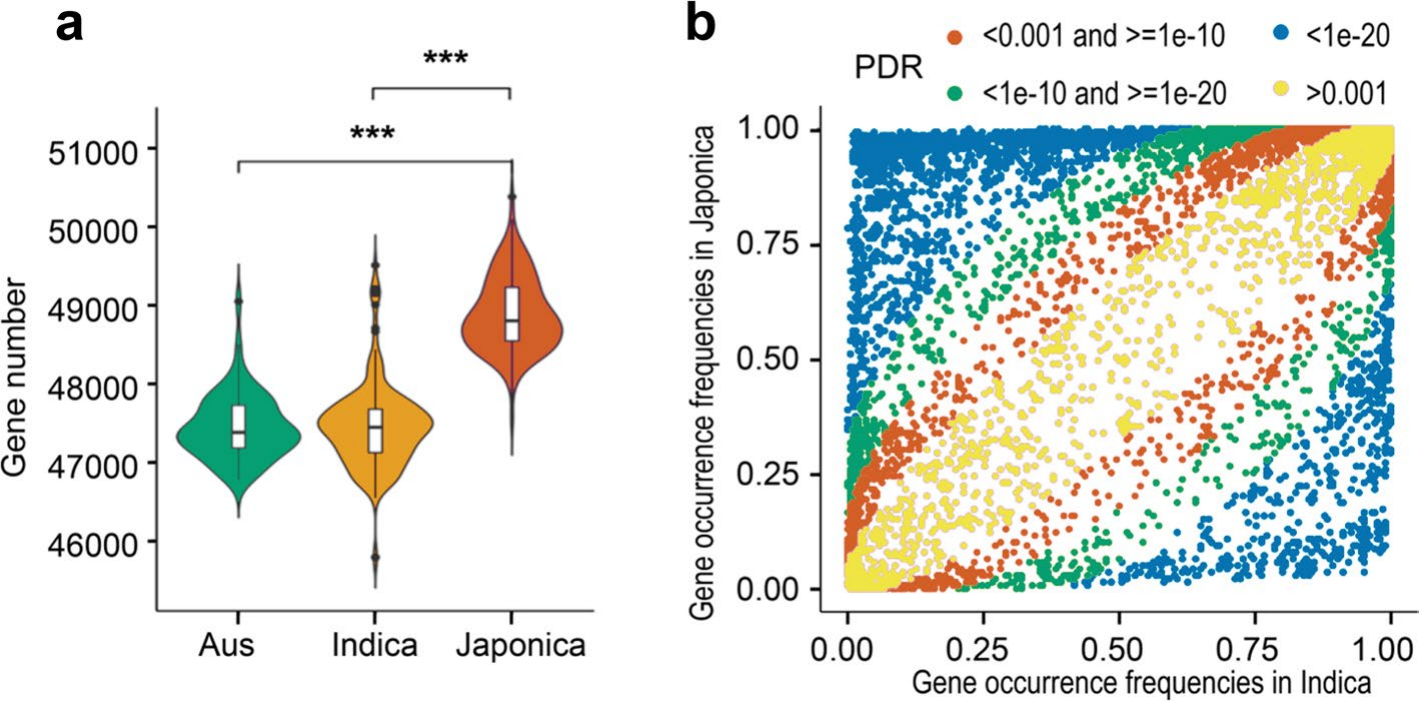
Gene number and frequency analysis among different rice subpopulations based on pangenome. **a** Violin plots showing gene abundance for the *Aus*, *Indica*, and *Japonica*. Significance differences between groups are indicated (***p-value < 0.005 ). **b** Comparison of gene frequency between Indica and Japonica. Different colors indicate different *p*-value ranges

We observed a significant difference in average gene numbers between *Japonica*, *Indica*, and *Aus* (Fig. 3a)*. Japonica* contains the largest number of genes (48,884 ± 472), with fewer genes in *Indica* (47,455 ± 537) and *Aus* (47,441 ± 405). The difference in average gene number hides a complex pattern of increases and decreases in the frequency of specific genes (Fig. 3b). A total of 978 genes show increased frequency in *Japonica*, while 2986 genes show decreased frequency. Genes showing increased frequency are enriched in functions associated with DNA integration (Additional file 2: Table S20), while genes showing decreased frequency are annotated with disease resistance terms, including pathogenesis and defense response (Additional file 2: Table S21). Among the 2986 genes with lower frequency in *Indica*, 116 (3.8%) genes are absent from the Nipponbare reference. In contrast, of the 978 genes exhibiting higher frequency in *Indica*, 513 (52.5%) genes are absent from the Nipponbare reference, with 482 genes derived from the *Indica* rice genomes. This reflects differences in gene content between sub-species at the population level.

### Population structure analysis based on pangenome PAVs

**Fig. 4:**
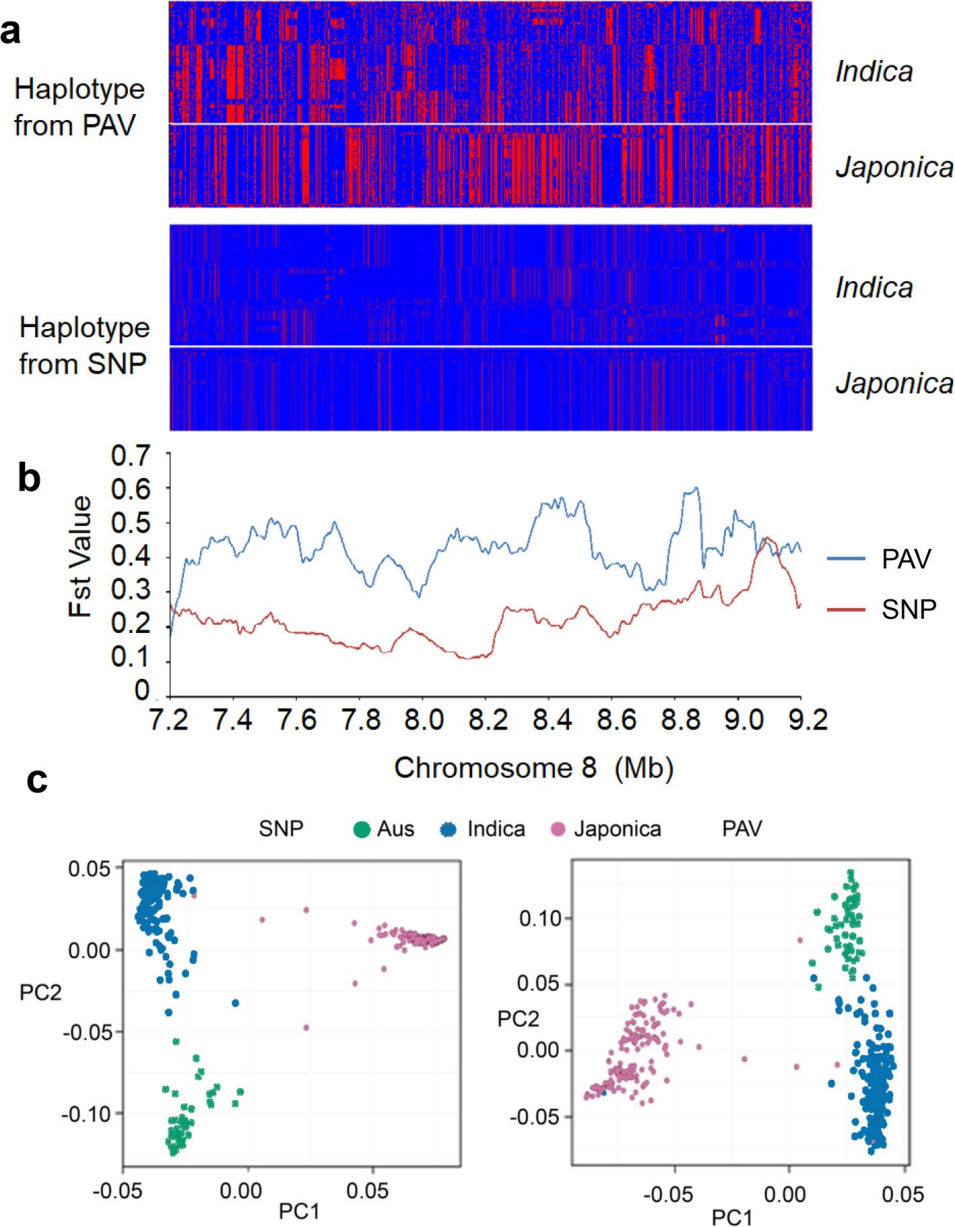
Population structure analysis based on PAVs and SNPs. **a** Haplotype landscape from PAVs and SNPs in the 7.2–9.2-Mb interval of chromosome 8 using the pangenome coordinate. **b** Fst calculated using PAV and SNP data in the 7.2–9.2-Mb interval of chromosome 8 using the pangenome coordinate. The red line indicates the Fst values calculated from SNPs, and the blue line indicates the Fst values calculated from PAVs. **c** PCA plot showing the population structure of different rice accessions which were generated by PAV and SNP data

We performed population genetic analysis in the international panel using PAVs and compared the results with SNP-based analysis. The genetic differentiation between populations estimated based on fixation index (Fst) using the SNPs (*Japonica*-*Indica*: 0.476 ±0.207, *Japonica-Aus*: 0.525 ±0.205, and *Indica-Aus*: 0.304 ±0.158) is higher than that calculated using PAVs (*Japonica-Indica*: 0.416 ±0.183, *Japonica-Aus*: 0.430 ±0.184, and *India-Aus*: 0.204 ±0.128) (Additional file 2: Table S22). Fst analysis results show similar distribution trends between PAVs and SNPs on the whole genome scale (Additional file 1: Fig. S23). SNP-based analysis shared genetic differentiation regions with PAV-based analysis (within the top 1% Fst windows) between populations. For example, SNP and PAV results share 33 out of 54 of the *Japonica*-*Indica* differentiation regions, containing 376 genes. We analyzed 15 well-studied rice domestication and breeding improvement-associated genes and compared the Fst detection between SNPs and PAVs. Among these 15 genes, three were within the top 10% of differentiation regions among *Indica*, *Japonica*, and *Aus* subpopulations using SNPs and PAVs (Additional file 2: Table S23). However, we also detected regions displaying significant differences between Fst values based on PAVs and SNPs. To investigate this discrepancy further, we selected a prominent region at 7.2–9.2 Mbp of chromosome 8, where we observed a much higher Fst value between *Indica* and *Japonica* calculated by PAVs than that was calculated by SNPs (Fig. 4a, b). Further analysis revealed that PAVs could detect more genetic diversity than SNPs in this region (Fig. 4a, b). The length of this region is about 1600 kb in the Nipponbare genome, while in the pangenome, the interval is 2 Mb in size, with 271 annotated genes, of which 162 are transposons. This demonstrates our linear pangenome can capture missing genetic diversity in the single reference genome.

PAV-based population structure shows similar clustering to SNP-based phylogeny, with 413 accessions clustered into three main subpopulations. However, the PAV-based phylogeny does not cluster individuals completely according to subpopulations, and the PAV-based PCA suggests a greater variation between rice accessions than the SNP-based analyses (Fig. 4c). For example, accessions in *Indica* and *Aus* subpopulations were grouped into two clusters compared with the SNP-based PCA results, and some accessions in the *Indica* subpopulation clustered with the *Aus* subpopulation. A similar pattern was observed in the PAV-based phylogeny with 73 *Indica* accessions clustering with the *Aus* subpopulation (Additional file 1: Fig. S24).

### Using pangenome for PAV-based GWAS

As a pangenome permits the genotyping of a greater amount of genetic diversity than a single reference, it supports more powerful genetic analysis and facilitates capturing missing heritability. To explore this additional potential, particularly for identifying functional PAVs underlying QTLs, we conducted GWAS for two important agronomic traits of rice, namely thousand grain weight (TGW) and plant height (PH), using SNPs genotyped from the Nipponbare reference genome and PAVs genotyped across the pangenome.

**Fig. 5:**
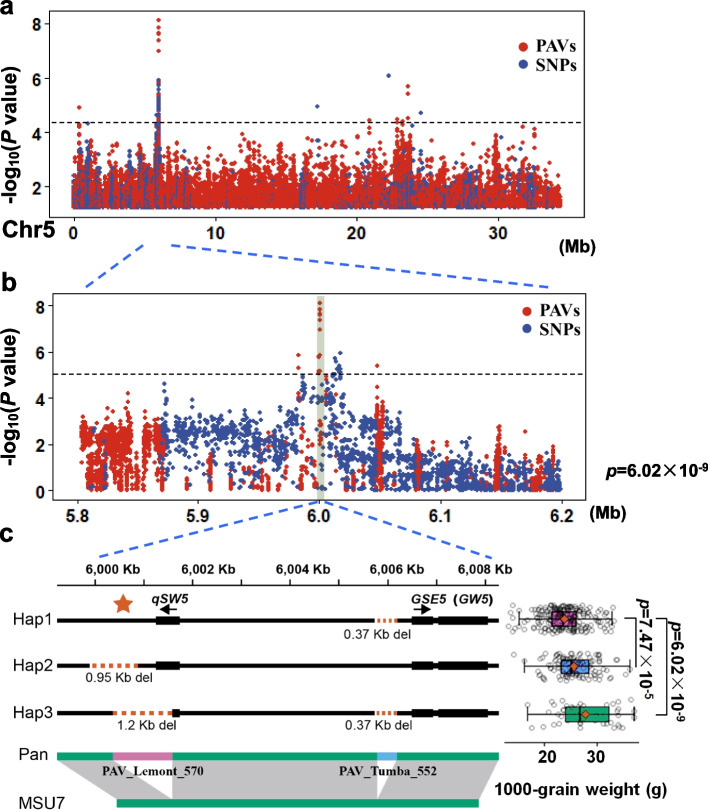
GWAS of thousand grain weight in 413 international accessions. **a** Manhattan plots of SNP-GWAS and PAV-GWAS in chromosome 5 for the thousand grain weight trait. SNPs and PAVs were genotyped in the pangenome. **b** A zoomed view in 5.8 to 6.2 Mb of Manhattan plots of SNP-GWAS and PAV-GWAS in chromosome 5 for the thousand grain weight trait. **c** Haplotype analysis for the loci regulating the thousand grain traits. Hap1 contains the 370 bp deletion and without the 1212 bp and 950 bp deletions. Hap2 contains the 950bp deletion and without the 370bp deletion. Hap3 contains the 370 bp deletion as well as the 1212 bp deletions. PAV_Lemont_570 and PAV_Tumba_552 denoted the PAVs. The PAVs’ locations are shown in the pangenome by comparing them to the Nipponbare reference genome (MSU7.0). The red star indicates the position of the highest −log10(*p*-value) in PAV-GWAS

For TGW, the SNP-GWAS identified 354 significant associations (Additional file 1: Fig. S25a), with one of the most significant signals located in Nipponbare (NIP) Chr5: 5,375,764 bp (pangenome Chr5: 6,017,339 bp), which is 9063 bp away from *GW5*, a known functional gene controlling rice grain weight [31]. However, none of the associated SNPs were the causal variations of *GW5*, which are two PAVs (950 bp and 1212 bp) in this region [31]. Using the PAVs genotyped from the pangenome to perform GWAS can also narrow down the association signal in the same interval as SNP-GWAS (Fig. 5a and Additional file 1: Fig. S25a). Notably, the most significant associated signal directly pinpointed the causal variations of *GW5* (Fig. 5b, c). We further analyzed the PAV genotypes, and three haplotypes (Hap1-3) according to the pangenome were identified in our GWAS panel. The accessions with Hap1 (containing the 370bp deletion and without the 1212 bp and 950 bp deletions) showed significantly lower grain weight than accessions with the other two haplotypes (Hap2, Hap3) with *p*-values (two-tailed Student’s *t* test) of 7.47×10^−5^ and 6.02×10^−9^ respectively (Fig. 5c). This result is in accordance with a previous study, which demonstrated that the 950 bp deletion decreased the expression of the functional gene (*qSW5*), while the 1212 bp deletion disrupts the coding region of *qSW5*, leading to TGW phenotype variations [31].

SNP-GWAS identified 37 SNPs associated with PH in rice (Additional file 1: Fig. S25b). Similar to the GWAS results of TGW, both SNP- and PAV-GWAS were able to locate previously characterized locus harboring the “Green Revolution Gene” (*sd1*) [32]. Similarly, the most significant PAV is located inside the causal PAV of the *sd1* gene (Additional file 1: Fig. S26) [32]. Statistical analysis showed that this PAV is significantly correlated with the PH phenotype (two-tailed Student’s *t* test, *p*-value: 3.3× 10^−29^), further validating the accuracy and efficiency of GWAS using PAVs genotyped from our pangenome.

**Fig. 6:**
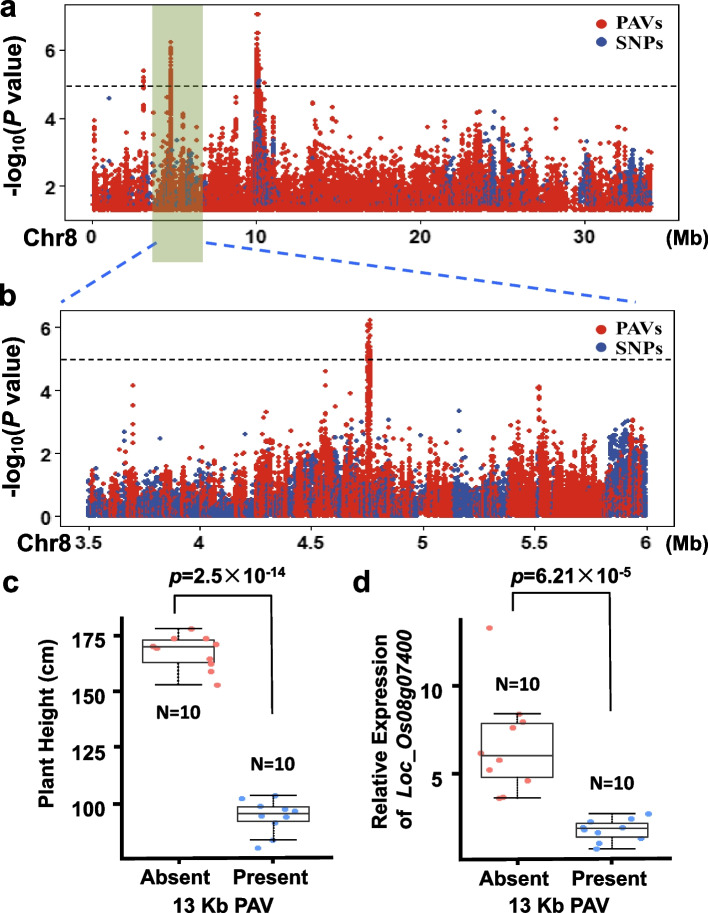
GWAS of plant height in 413 international rice accessions. **a** Manhattan plot of SNP- and PAV-GWAS in chromosome 8 for plant height. SNPs and PAVs were genotyped using the pangenome. The most significant hit was detected around the 10-Mb region. Two regions (PAV markers at 3,117,040 and 4,761,060 bp) also surpass the significance threshold of 5 −log(P). **b** A zoomed view of the 3.5- to 6-Mb region of the Manhattan plot of SNP-GWAS and PAV-GWAS in chromosome 8 for plant height. **c, d** Box plot of plant height and relative expressions level of *Loc_Os08g07400* in 10 accessions contains (present, blue points) and 10 accessions without (absent, red points) the 13-kb insertion. The *p*-values were determined using two-tailed Student’s *t* tests. The middle bars represent the median, and the bottom and top of each box represent the 25th and 75th percentiles. The whiskers extend to 1.5 times the interquartile range

Interestingly, we also identified a novel locus (*qPH8-1*) controlling PH in rice on chromosome 8 by PAV-GWAS (interval 4,660,000–4,860,000 bp in the pangenome) that was not identified by SNP-GWAS (Fig. 6a, b). The most significant PAV was in a 13-kb region containing two retrotransposon genes (*LOC_Os08g07410, LOC_Os08g07420*), located 1 kb upstream of *LOC_Os08g07400* (Additional file 1: Fig. S27). This sequence was present in 288 out of the 413 accessions and validated in randomly selected accessions using PCR amplification (Additional file 1: Fig. S28). The accessions without the 13-kb sequence had significantly greater plant height (two-tailed Student’s *t* test, *p*-value: 5.7 × 10^−20^) than those with the sequence. Expression analysis showed that the presence and absence of this 13-kb sequence significantly correlated with the expression level of *LOC_Os08g07400*, located 1 kb downstream from the 13-kb sequence (Fig. 6c, d). These results suggested that this PAV, which is possibly caused by retrotransposon movement, may impact downstream gene expression and the plant height phenotype.

**Fig. 7:**
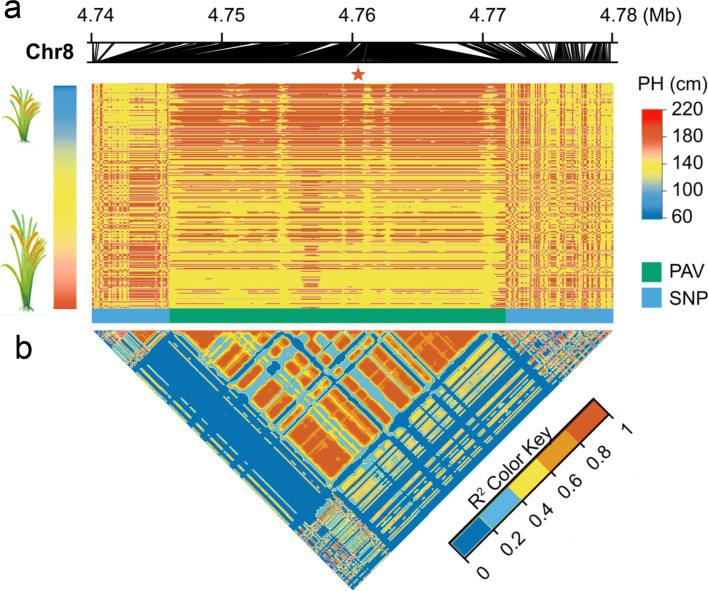
Population-wide landscape of PAVs and SNPs in the plant height QTL in Chromosome 8. **a** Genotype of PAVs and SNPs in the plant height QTL in Chromosome 8. The red bar indicates the presence of the PAVs; the yellow bar indicates the absence of the PAVs. The five-pointed red star indicates the position of the peak association PAV marker. The plant height phenotypes of each accession are sorted and displayed in the sidebar. **b** Linkage disequilibrium (LD) heatmap shows the regions surrounding the strong peaks of the PAV-GWAS

We further investigated the potential mechanism underlying the discordance of results between SNP-GWAS and PAV-GWAS in *qPH8-1*. We examined the landscape of genome structure at the population level and examined the relationship between the 13-kb PAV and the nearby SNPs. The results showed the presence or absence of the 13-kb sequence strongly correlated with the plant height phenotype. In contrast, the SNPs on both sides of the PAV did not (Fig. 7a). Linkage disequilibrium (LD) analysis further demonstrated the PAV interval formed an independent LD block. At the same time, the PAV genotype was not correlated with the SNP genotype (Fig. 7b).

## Discussion

### PSVCP provides an accurate and robust tool for pangenome construction and analysis

Many genomics studies include mapping sequencing data to reference genomes to identify genomic variations. However, these analyses suffer from bias due to the use of a single reference genome. Reference bias is especially problematic in the analysis of SVs, which are major forms of genomic variation in plants [33]. As an alternative, a pangenome can represent the genomic diversity of a species or population better than a single reference. Using a pangenome as a reference for sequencing data mapping supports accurate downstream analysis and avoids reference bias.

Currently, the most advanced method for pangenome construction and analysis is the graph-based strategy, which maintains the position of variable genetic information for each accession [17–19]. However, the graph-based pangenome approach also faces some challenges. This strategy is still in the early development stage, and we lack a standard approach for graph-based pangenome construction and analysis. Furthermore, using the current graph-based pangenome, it is still difficult to detect complex structural variations such as translocations and inversions that are common in plants. Many pangenomic approaches stem from research on the human genome, which has a lower level of genomic variations between individuals than plant genomes. As such, the current graph-based pangenomes may not fully represent large structural variations, which is still a great challenge for plant pangenomic studies [34]. Furthermore, since plants contain complex repeat regions, they require significant computational resources for graph-based pangenome construction, especially for crops with large genome sizes. There are still insufficient tools available for the analysis of graph-based pangenomes. For example, while pangenome mapping algorithms have been developed for mapping reads to sequence graphs [35], none could replace linear genome-based mapping tools.

Because of the challenges in applying graph-based pangenomes, the linear pangenome is valuable for both functional genomic studies and breeding applications. In this study, we developed a new pipeline for constructing linear pangenomes (PSVCP) and aimed to overcome the bottleneck of other linear pangenome strategies. A major challenge for current linear pangenome construction strategies is the ability to accurately embed the newly identified PAV sequences into the linear reference genome. In several recent pangenome studies, including the 3010 rice pangenome [15], the tomato pangenome [36], and *Brassica napus* pangenomes [7], novel sequences are placed as separate contigs that do not consider their genomic context. This can limit further use of the pangenome in downstream gene mapping or functional validation of the PAVs, since the nearby sequences may be important and informative for the functional characterization. For example, a Pan-SV analysis in tomatoes revealed that the majority of gene-associated SVs are in cis-regulatory regions, and many are associated with subtle changes in gene expression [37].

To address this issue, PSVCP was designed to place novel sequences into the correct genome position, providing an accurate genomic map for functional genomic studies. The accuracy of the placement of the novel sequences by PSVCP was confirmed in the present study by successfully identifying the existence of the novel sequences and the sequence surrounding them by PCR amplification followed by sequencing. The advantage of our strategy was further demonstrated by GWAS analysis using PAVs genotyped in our pangenome. Our PAV-GWAS successfully captured the casual structural variations of TGW and PH, which are not available in the Nipponbare reference genome or are difficult for functional characterization without the sequence information surrounding them. Furthermore, the pangenome constructed using PSVCP benefits from its linear format, which can directly integrate with currently available bioinformatics pipelines such as GATK [38] for genome variation discovery, and JBrowse [39] for genome visualization.

PSVCP is robust in placing novel PAV sequences into the linear pangenome; it may be limited to display more complex SVs such as translocations and inversions, which is a challenge in current pangenomics studies, even for the advanced graph-based pangenome. In addition, due to its read length, short-read sequencing data may have lower sensitivity for SV detection compared with long-read sequencing data.

### PAVs provide insights into rice population structure

Most population structure studies are currently performed using SNPs [40]; however, structural variants such as PAVs are increasingly used since they provide additional information about the population structure [7, 19, 36]. SV-based population structure studies are likely to improve our understanding of the adaptation and evolution of species.

The rice pangenome constructed in this study contains novel genome sequences and annotated genes from comprehensive comparative genomic analysis. Our results indicated that compared to SNPs, PAVs provided novel insights into rice evolution when used to identify genetic differentiation regions using Fst and phylogenetic inferences. In most cases, we found that SNP- and PAV-based population structure analyses shared similar patterns of Fst value changes along the chromosomes. However, in some genomic regions, PAV-based analysis has significant different Fst values compared with SNP-based results, providing higher resolution to differentiate the population structure in these regions. As an example, the present study identified a 1.6-Mb interval in chromosome 8 displaying much higher Fst values in PAV-based analysis between *Japonica* and *Indica* than in SNP-based analysis. Higher frequencies of novel sequence insertions and more haplotype diversity were discovered in this region using PAVs than SNPs, which may cause by transposon movement. This suggests that SNPs may underestimate genetic differentiation in some highly diverse genomic regions containing PAVs. These results demonstrate that PAV genotypes in our pangenome can provide additional power and information in analyzing genomic divergence and evolution.

Detailed analysis of the PAVs identified in the present study indicated that the majority of the newly inserted PAV sequences are transposable elements. Compared with SNP-based phylogeny, PAV-based phylogeny shows that some *Indica* accessions clustered with the *Aus* subpopulation, which is consistent with the TE-insertion phylogeny analysis using the 3000 rice accessions [15]. This result also reflects the fact that *Aus* and *Indica* contain more common TE-insertions, since the divergence of the *Indica/Aus* lineages occurred more recently (~540,000 years ago) than the divergence of *Indica/Japonica* (~800,000 years ago) [26]. Additionally, introgression is potentially detected between *Indica* and *Aus* subpopulations based on the PAV data, consistent with previous studies showing that *Indica* accessions contain *Aus* introgressions [41] and *Indica* and *Aus* show closer genetic affinity [42]. The topological variation between SNP- and PAV-based phylogenetic analyses are consistent with observations in other plants such as *Arabidopsis thaliana* [43], *Amborella trichopoda* [29], green millet *Setaria viridis* [44], and *Brassica oleracea* [7], showing that PAVs or SVs can provide additional resolution to characterize population structure that might be associated with transposon movement during genome evolution, highlighting the value of using PAVs or SVs in addition to SNPs in assessing species evolution.

### PAV-based GWAS provides additional power to identify causal variants

Most GWAS analysis uses SNPs identified from a single reference genome as markers to detect marker-trait associations. However, recent studies suggest that SVs, including PAVs, contribute to and explain more phenotypic variation than SNPs for many traits [45]. Phenotypes associated with regions that are absent in the reference genome can only be mapped to a region in the LD block linked with the PAV. However, this association cannot be identified if the PAV haplotypes are not in LD with SNPs surrounding them, as we observed in our results (Fig. 7a). Furthermore, using variations identified from a single reference in GWAS may cause bias, which weakens the ability of GWAS to identify associations. For example, a maize gene conferring resistance to sugarcane mosaic virus is present in the B73 reference genome but not in the PH207 reference. Conducting GWAS using SNPs genotyped using the B73 reference can identify the gene, while the PH207 cannot [46]. Using PAVs identified from a pangenome would resolve such problems, and PAV-GWAS can be a valuable complement to SNP-GWAS. For example, a recent study in *Brassica napus* showed that a PAV-based pangenome-wide association study could directly pinpoint the causal SVs for silique length, seed weight, and flowering time [47].

In this study, PAVs were genotyped from the pangenome constructed by the PSVCP pipeline and used for GWAS analysis of TGW and PH in an international rice panel. Both PAV- and SNP-GWAS methods can identify previous characterized QTLs, such as *GW5* for TGW and *sd1* for PH. Surprisingly, the peak PAV-GWAS signals are directly and accurately located in the functional PAVs causing the phenotypic variations. In contrast, the most significant signal for SNP-GWAS can only identify the approximate location of the causal variants.

More importantly, PAV-GWAS can identify new candidate causal variations that SNP-GWAS cannot discover. In the present study, a 13-kb PAV containing two retrotransposons was found to be strongly associated with plant height using PAV-GWAS, and this variant was not identified using SNP-GWAS. Transposon movements are important sources of phenotypic variations. A GWAS study in tomatoes based on TE-insertion polymorphisms revealed that transposon movement was associated with leaf morphology and fruit color [48]. Further investigation of the 13-kb sequence showed that it was located 1 kb upstream from *LOC_Os08g07400*, whose expression was significantly associated with the presence and absence of the 13-kb sequence. These results suggest that retrotransposon movement in this locus may lead to phenotypic variation by affecting the promoter region of *LOC_Os08g07400*.

To unravel why SNP-GWAS cannot identify this locus, we investigated the candidate variant region at the population level. Our results showed that no SNPs were found in the 13 Kb PAV sequence, while SNPs located near the 13-kb PAV sequence show a poor correlation with the PAV, with no association between SNPs and the plant height phenotype. TEs having a low LD with nearby SNPs were observed in other genomic studies in rice and tomato [49]. Akakpo et al. reported that TE-GWAS could identify a signal associated with rice grain width on chromosome 4 that was missing in SNP-GWAS [50]. Recent independent retrotransposon insertion may cause the low LD of SNPs by breaking previous linkage disequilibrium across haplotypes [51, 52]. However, further investigation is required to understand how they affect functional gene expression and phenotype variations. Our study demonstrates that the PAV-based pangenome-wide association analysis is a powerful approach to detect and dissect the genetic variants causing phenotypic variation of agronomical traits.

## Conclusions

A new strategy and pipeline to construct a linear pangenome by whole genome comparison were developed in the present study. This strategy supported the construction of a linear pangenome that can solve the problems of preserving the location information of SVs and facilitates downstream pangenomic analysis. A rice pangenome was constructed using 12 complete genomes spanning all rice subpopulations. Downstream population analysis demonstrated that using the pangenome provided insights into the rice population structure and evolution, which are not available by analysis using SNPs from a single reference. GWAS analysis using PAVs genotyped from the pangenome revealed a significant improvement in power, especially in characterizing causal PAVs. The new pangenome construction pipeline and the rice pangenome provide a novel framework for future pangenomic studies in rice and other plants.

## Methods

### Plant materials

The RPD panel [23] contains about 2000 accessions and is a representative collection from 96 countries. The 413 accessions (Additional file 2: Table S24) from this study were selected from the RPD panel [23] based on diversity and representativeness calculated using the whole genome genotypes, representing the genetic diversity of worldwide Asian rice. Seeds for 413 accessions were sown on July 28th, 2020, in Guangzhou, Guangdong, China. High-molecular-weight genomic DNA was extracted from 30-day-old leaves following a standard CTAB (hexadecyltrimethylammonium bromide) protocol. Sequencing was performed on the Illumina NovaSeq6000 platform (BerryGenomics, China). A fastx_toolkit (http://hannonlab.cshl.edu/fastx_toolkit) was used to remove adaptor and low-quality reads. All raw sequence data have been deposited in the NCBI sequence read archive (BioProject accession PRJNA820969). Plant height and thousand grain weight were assessed at the mature growth stage with three biological replicates.

### Construction of the pangenome

Data for twelve assembled genomes were downloaded from the Rice Resource Center (https://ricerc.sicau.edu.cn/) [13], representing Nipponbare, Lemont, NamRoo, LJ, CN1, R498, TM, Tumba, FH838, N22, Basmati1, and CG14. We employed an iterative strategy to construct the pangenome. First, we performed a pairwise collinearity comparison between Nipponbare and Lemont using MUMmer v4.0.0 [53], with parameters: “--maxgap 500 --mincluster 1000 --diagdiff 20.” Nipponbare was named as ref0. We used Assemblytics V1.2.1 [54] to detect and analyze variants from MUMmer. SVs were identified by comparison of the first genome (Lemont) with the Nipponbare reference genome assembly (ref0). The insertions larger than 50 bp were identified and incorporated to generate the new reference genome (ref1). The ref1 genome was then further compared with the next genome iteratively until all genomes were incorporated into the pangenome (Additional file 2: Table S1).

### Short-read data processing for PAV-GWAS

Paired-end short-read sequencing data for each accession was aligned to the pangenome using BWA-MEM with default settings [55]. Mapping results were sorted using Picard (https://github.com/broadinstitute/picard) [38] and filtered using SAMtools [56], retaining reads with a mapping quality over 20. We used the SAMtools with the parameters: “-F 4 -F 256” to remove reads that did not map to the pangenome or mapped to the pangenome repeatedly. Using the pangenome as the reference genome, we detected the coverage of each accession in every 20 bp region by Mosdepth [57] with the parameters: “-b 20.” Two adjacent 20-bp regions were merged if adjacent sequences showed the same presence or absence pattern (had coverage of ≥5 reads or < 5 reads).

### PAV identification

PAVs were called based on the mapping coverage of sequencing reads to the pangenome for each accession. The breakpoint positions of each accession were recorded, and a complete set of all breakpoint positions (the breakpoints’ union) were generated. Every segment with two adjacent breakpoints from the breakpoints’ union is defined as a new PAV region, named by the adjacent left breakpoint position. Each accession was re-called using the integrated PAV genotypes. All accessions’ new PAV genotypes were combined by row, generating a PAV genotype matrix with accession names as row labels and the adjacent left breakpoint position as column labels. The PAV genotype matrix was further filtered by MAF>0.05.

### Gene PAV detection

A gene was considered missing when the horizontal coverage across the CDS is less than 95% and the vertical coverage less than two, as used in the 3K-RG study [15] using Mosdepth v0.2.6 (-Q 20 -i 2 -x) [57]. A gene PAV matrix was generated showing the presence or absence of each gene for each accession. The statistical significance of gene frequency changes was calculated using Fisher’s exact test. *P*-values were adjusted for multiple comparisons using the Bonferroni method as implemented in p.adjust from R v3.5.0. Genes with an adjusted *p*-value<0.001 and difference frequency between groups ≥10% [36] were defined as significant.

### Potential translocation and inversion identification

During pangenome construction, the PAV positions were recorded and updated in an annotation file in “gff” format. We used seqkit v1.2 [58] with the parameters “seqkit subseq -gtf pan.pav.gff -o pan_pav.fa pan.fa” to get the PAV sequences. Then we aligned the PAV sequences against the reference genome using BLASTN [59] with parameters “-evalue 1e-10 -perc_identity 95 -word_size 1000.” PAV sequences larger than 1kb and matched to 2 or more sequences on pangenome positions with sequence similarity greater than 95% were identified as potential translocations. The positions of the translocations were recorded. When checking short-read mapping near breakpoints on the Integrative Genomics Viewer (IGV) [60], we found that accessions did not contain the translocations can have reads covering the breakpoints with 1–15 mismatched bases. To filter these reads, we set the threshold for a mismatched base to 19bp. Reads spanned at least 39 bp (19 bp on the left and 19 bp on the right) are used to determine whether the accession’s sequencing read covers the breakpoint. We genotyped the presence or absence of translocations for 413 accessions by checking the read mapping around breakpoints, with reads spanning a 39-bp region with a conjunction point at the center. For each accession, we used SAM tools [56] to collect the short reads near breakpoint with parameters “samtools view BamFile Chr:(position_of_breakpoint-20)-(position_of_breakpoint+20).” Then we counted the number of the reads spanning a 39-bp region with a conjunction point at the center. If the mapping coverage of this breakpoint was less than 5×, we defined the genotype of the breakpoint as absence (“A”). Otherwise, we defined the genotype (≥5×) as presence (“C”). We identified inversions by comparing each genome used for pangenome construction with the pangenome using MUMmer v4.0.0 [53] and Assemblytics V1.2.1 [54]. We further genotyped each potential inversion in the pangenome using the short-read sequencing data of 413 accessions by the same method used for translocation genotyping (examining the mapping coverage of the 39-bp region with breakpoints in the center).

### Short-read sequencing data processing for SNP-GWAS

Short-read sequencing data were aligned to the Nipponbare reference genome using BWA-MEM [55]. The results were sorted using Picard and filtered using SAMtools, retaining reads with a mapping quality over 20. Nucleotide variants for each accession were detected using HaplotypeCaller in GATK (v3.8-1-0) [38] with the default parameters. Population nucleotide variants were called using CombineGVCFs and GenotypeGVCFs tools in GATK. Finally, we used the SelectVariants and VariantFiltration tool in GATK to filter the genotype of the population.

### GWAS analysis

To construct the PAV genotype map for GWAS, we used “A” to represent “Absent” and “C” to represent “Present” in the HapMap genotype file. PAVs and SNPs were selected for GWAS analysis based on the criteria of missing data <15% and MAF >0.05. GWAS was performed using a mixed linear model (MLM) with kinship matrix and principal component analysis in GAPIT version 2 [61]. The significance cutoff was defined as the threshold of –log_10_(*p*) <5. Manhattan plots were produced using the CMplot package (https://github.com/YinLiLin/R-CMplot) in R v3.5.0.

### GO analysis

Functional annotation was performed using Blast2GO v2.5 [62]. Genes were aligned to the proteins in the Viridiplantae database using BLASTP [59] (*E*-values <1 × 10^−5^). Gene ontology (GO) analysis was conducted using topGO [63], and Fisher’s exact test with “elim” was used to correct for multiple comparisons.

### Population structure and genotype analysis

Filtered PAV and SNP data were used for the population structure study. SNP-based and PAV-based phylogenetic trees of 413 rice accessions were constructed by IQ-tree [64] using a maximum likelihood method (with the alrt 1000 -bb 1000), respectively. SNP-based and PAV-based principal component analyses were performed with GCTA (Genome-wide Complex Trait Analysis) v1.93.2 [65]. SNP-based and PAV-based Fst values were calculated using a 100-kb sliding window (with a 10-kb step for FST value calculation) using VCFtools [66]. Plink v1.9 [67] was used to detect the minor allele frequency between genic PAV and intergenic PAV.

### TE analysis

A de novo transposable element (TE) library was generated for the rice pangenome using EDTA v1 (--sensitive 1 --anno 1 --species Rice) [68]. Using BLAST+ v 2.2.3 [59], the representative retrotransposon TE families in Carpentier et al. [26] were used to search the rice pangenome library to identify the whole genome-wide TEs (with >85% sequence identity and e-value < 10^−5^).

### PCR validation of the 13-kb insertion in *qPH8-1*

For validation of the 13-kb insertion in *qPH8-1*, primers were designed (Additional file 1: Fig. S28a and Additional file 2: Table S25) on both sides of the sequence spanning the whole insertion. Accessions with or without the insertion were selected for validation. Seedlings of selected accessions were used for DNA extraction by the DNeasy Plant Mini Kit (Qiagen, Germany). The DNA samples of the selected rice accessions were used as templates for PCR by KOD-FX polymerase (Toyobo, Japan) using the primers described above.

## Supplementary Information

**Additional file 1:** **Supplemental figures 1-28.**

**Additional file 2:** **Supplemental tables 1-25.**

**Additional file 3:** **Supplemental notes.**

**Additional file 4.** Uncropped images for the blots in Figure S3 and Figure S28.

**Additional file 5.** Review history.

## Data Availability

The selected twelve published rice genome assemblies from Qian’s study [13] were available in the Rice Resource Center database [69]. The raw read data (FASTQ files) of 413 accessions were uploaded to NCBI’s sequence read archive (BioProject accession PRJNA820969) [70]. The PacBio long-read sequences and genome assemblies of two lines (IRRI2K_86 and IRRI2K_91) are available at NCBI (BioProject accession PRJNA886102) [71]. The source code of PSVCP is available under GPL-3.0 license on GitHub [72] and Zenodo [73]. The constructed rice pangenome, the annotated files, and the PAV matrix of 413 accessions in our study and the PAV matrix of 1032 selected from the 3K rice genome study can be accessed OSF [74].

## References

[1] Tomlinson I (2013). Doubling food production to feed the 9 billion: a critical perspective on a key discourse of food security in the UK. J Rural Stud.

[2] Ehrlich PR, Harte J (2015). To feed the world in 2050 will require a global revolution. Proc Natl Acad Sci U S A.

[3] Varshney RK, Graner A, Sorrells ME (2005). Genomics-assisted breeding for crop improvement. Trends Plant Sci.

[4] He T, Li C (2020). Harness the power of genomic selection and the potential of germplasm in crop breeding for global food security in the era with rapid climate change. Crop J.

[5] Bayer PE, Golicz AA, Scheben A, Batley J, Edwards D (2020). Plant pan-genomes are the new reference. Nat Plants.

[6] Tettelin H, Masignani V, Cieslewicz MJ, Donati C, Medini D, Ward NL (2005). Genome analysis of multiple pathogenic isolates of Streptococcus agalactiae: implications for the microbial “pan-genome”. Proc Natl Acad Sci U S A.

[7] Golicz AA, Bayer PE, Barker GC, Edger PP, Kim H, Martinez PA (2016). The pangenome of an agronomically important crop plant Brassica oleracea. Nat Commun.

[8] Montenegro JD, Golicz AA, Bayer PE, Hurgobin B, Lee H, Chan CKK (2017). The pangenome of hexaploid bread wheat. Plant J.

[9] Jayakodi M, Padmarasu S, Haberer G, Bonthala VS, Gundlach H, Monat C (2020). The barley pan-genome reveals the hidden legacy of mutation breeding. Nature..

[10] Rijzaani H, Bayer PE, Rouard M, Doležel J, Batley J, Edwards D (2022). The pangenome of banana highlights differences between genera and genomes. Plant Genome.

[11] Zhao J, Bayer PE, Ruperao P, Saxena RK, Khan AW, Golicz AA (2020). Trait associations in the pangenome of pigeon pea (*Cajanus cajan*). Plant Biotechnol J.

[12] Zhao Q, Feng Q, Lu H, Li Y, Wang A, Tian Q (2018). Pan-genome analysis highlights the extent of genomic variation in cultivated and wild rice. Nat Genet.

[13] Qin P, Lu H, Du H, Wang H, Chen W, Chen Z (2021). Pan-genome analysis of 33 genetically diverse rice accessions reveals hidden genomic variations. Cell..

[14] Zhou Y, Chebotarov D, Kudrna D, Llaca V, Lee S, Rajasekar S (2020). A platinum standard pan-genome resource that represents the population structure of Asian rice. Sci Data.

[15] Wang W, Mauleon R, Hu Z, Chebotarov D, Tai S, Wu Z (2018). Genomic variation in 3,010 diverse accessions of Asian cultivated rice. Nature..

[16] Liu Y, Tian Z (2020). From one linear genome to a graph-based pan-genome: a new era for genomics. Sci China Life Sci.

[17] Zhou Y, Zhang Z, Bao Z, Li H, Lyu Y, Zan Y, et al. Graph pangenome captures missing heritability and empowers tomato breeding. Nature. 2022. 10.1038/s41586-022-04808-9. 10.1038/s41586-022-04808-9 PMC9200638 35676474

[18] Eizenga JM, Novak AM, Sibbesen JA, Heumos S, Ghaffaari A, Hickey G (2020). Pangenome graphs. Annu Rev Genom Hum G.

[19] Liu Y, Du H, Li P, Shen Y, Peng H, Liu S (2020). Pan-genome of wild and cultivated soybeans. Cell..

[20] Bayer PE, Petereit J, Durant É, Monat C, Rouard M, Hu H (2022). Wheat panache: a pangenome graph database representing presence–absence variation across sixteen bread wheat genomes. Plant Genome.

[21] Bayer PE, Valliyodan B, Hu H, Marsh JI, Yuan Y, Vuong TD (2022). Sequencing the USDA core soybean collection reveals gene loss during domestication and breeding. Plant Genome.

[22] Shang L, Li X, He H, Yuan Q, Song Y, Wei Z (2022). A super pan-genomic landscape of rice. Cell Res.

[23] McCouch SR, Wright MH, Tung C, Maron LG, McNally KL, Fitzgerald M (2016). Open access resources for genome-wide association mapping in rice. Nat Commun.

[24] Kawahara Y, de la Bastide M, Hamilton JP, Kanamori H, McCombie WR, Ouyang S (2013). Improvement of the *Oryza sativa* Nipponbare reference genome using next generation sequence and optical map data. Rice..

[25] Simão FA, Waterhouse RM, Ioannidis P, Kriventseva EV, Zdobnov EM (2015). BUSCO: assessing genome assembly and annotation completeness with single-copy orthologs. Bioinformatics..

[26] Carpentier M, Manfroi E, Wei F, Wu H, Lasserre E, Llauro C (2019). Retrotranspositional landscape of Asian rice revealed by 3000 genomes. Nat Commun.

[27] Zhang J, Chen L, Xing F, Kudrna DA, Yao W, Copetti D (2016). Extensive sequence divergence between the reference genomes of two elite indica rice varieties Zhenshan 97 and Minghui 63. Proc Natl Acad Sci U S A.

[28] Sasaki T (2005). The map-based sequence of the rice genome. Nature..

[29] Hu H, Scheben A, Verpaalen B, Tirnaz S, Bayer PE, Hodel RGJ (2022). Amborella gene presence/absence variation is associated with abiotic stress responses that may contribute to environmental adaptation. New Phytol.

[30] Tao Y, Jordan DR, Mace ES (2020). A graph-based pan-genome guides biological discovery. Mol Plant.

[31] Liu J, Chen J, Zheng X, Wu F, Lin Q, Heng Y (2017). GW5 acts in the brassinosteroid signalling pathway to regulate grain width and weight in rice. Nat Plants.

[32] Sasaki A, Ashikari M, Ueguchi-Tanaka M, Itoh H, Nishimura A, Swapan D (2002). A mutant gibberellin-synthesis gene in rice. Nature..

[33] Khan AW, Garg V, Roorkiwal M, Golicz AA, Edwards D, Varshney RK (2020). Super-pangenome by integrating the wild side of a species for accelerated crop improvement. Trends Plant Sci.

[34] Hübner S (2022). Are we there yet? Driving the road to evolutionary graph-pangenomics. Curr Opin Plant Biol.

[35] Sirén J, Monlong J, Chang X, Novak AM, Eizenga JM, Markello C (2021). Pangenomics enables genotyping of known structural variants in 5202 diverse genomes. Science..

[36] Gao L, Gonda I, Sun H, Ma Q, Bao K, Tieman DM (2019). The tomato pan-genome uncovers new genes and a rare allele regulating fruit flavor. Nat Genet.

[37] Alonge M, Wang X, Benoit M, Soyk S, Pereira L, Zhang L (2020). Major impacts of widespread structural variation on gene expression and crop improvement in tomato. Cell..

[38] McKenna AHMBE (2010). The genome analysis toolkit: a MapReduce framework for analysing next-generation DNA sequencing data. Genome Res.

[39] Skinner ME, Uzilov AV, Stein LD, Mungall CJ, Holmes IH (2009). JBrowse: a next-generation genome browser. Genome Res.

[40] Morin PA, Martien KK, Taylor BL (2009). Assessing statistical power of SNPs for population structure and conservation studies. Mol Ecol Resour.

[41] Wang Q, Tang J, Han B, Huang X (2020). Advances in genome-wide association studies of complex traits in rice. Theor Appl Genet.

[42] Garris AJ, Tai TH, Coburn J, Kresovich S, McCouch S (2005). Genetic structure and diversity in *Oryza sativa* L. Genetics..

[43] Tan S, Zhong Y, Hou H, Yang S, Tian D (2012). Variation of presence/absence genes among *Arabidopsis* populations. BMC Evol Biol.

[44] Mamidi S, Healey A, Huang P, Grimwood J, Jenkins J, Barry K (2020). A genome resource for green millet *Setaria viridis* enables discovery of agronomically valuable loci. Nat Biotechnol.

[45] Gabur I, Chawla HS, Snowdon RJ, Parkin IAP (2019). Connecting genome structural variation with complex traits in crop plants. Theor Appl Genet.

[46] Gage JL, Vaillancourt B, Hamilton J. Multiple maise reference genomes impact the identification of variants by genome-wide association study in a diverse inbred panel. Plant Genome. 2019;12(2). 10.3835/plantgenome2018.09.0069. 10.3835/plantgenome2018.09.0069 PMC12810008 31290926

[47] Song J, Guan Z, Hu J, Guo C, Yang Z, Wang S (2020). Eight high-quality genomes reveal pan-genome architecture and ecotype differentiation of *Brassica napus*. Nat Plants.

[48] Domínguez M, Dugas E, Benchouaia M, Leduque B, Jiménez-Gómez JM, Colot V (2020). The impact of transposable elements on tomato diversity. Nat Commun.

[49] Yan H, Haak DC, Li S, Huang L, Bombarely A (2022). Exploring transposable element-based markers to identify allelic variations underlying agronomic traits in rice. Plant Commun.

[50] Akakpo R, Carpentier M, Ie Hsing Y, Panaud O (2020). The impact of transposable elements on the structure, evolution and function of the rice genome. New Phytol.

[51] Lewerentz J, Johansson A, Larsson J, Stenberg P (2022). Transposon activity, local duplications and propagation of structural variants across haplotypes drive the evolution of the drosophila S2 cell line. BMC Genomics.

[52] Lai J, Li Y, Messing J, Dooner HK (2005). Gene movement by Helitron transposons contributes to the haplotype variability of maise. Proc Natl Acad Sci U S A.

[53] Marçais G, Delcher AL, Phillippy AM, Coston R, Salzberg SL, Zimin A (2018). MUMmer4: a fast and versatile genome alignment system. PLoS Comput Biol.

[54] Nattestad M, Schatz MC (2016). Assemblytics: a web analytics tool for the detection of variants from an assembly. Bioinformatics..

[55] Li H. Aligning sequence reads, clone sequences and assembly contigs with BWA-MEM. arXiv. 2013;1303.3997v2.

[56] Li H, Handsaker B, Wysoker A, Fennell T, Ruan J, Homer N (2009). The sequence alignment/map format and SAMtools. Bioinformatics..

[57] Pedersen BS, Quinlan AR (2017). Mosdepth: quick coverage calculation for genomes and exomes. Bioinformatics..

[58] Shen W, Le S, Li Y, Hu F (2016). SeqKit: a cross-platform and ultrafast toolkit for FASTA/Q file manipulation. PLoS One.

[59] Camacho C, Coulouris G, Avagyan V, Ma N, Papadopoulos J, Bealer K (2009). BLAST+: architecture and applications. BMC Bioinformatics.

[60] Thorvaldsdóttir H, Robinson JT, Mesirov JP (2013). Integrative genomics viewer (IGV): high-performance genomics data visualisation and exploration. Brief Bioinform.

[61] Tang Y, Liu X, Wang J, Li M, Wang Q, Tian F (2016). GAPIT version 2: an enhanced integrated tool for genomic association and prediction. Plant Genome.

[62] Conesa A, Götz S, García-Gómez JM, Terol J, Talón M, Robles M (2005). Blast2GO: a universal tool for annotation, visualisation and analysis in functional genomics research. Bioinformatics..

[63] Alexa AAJR (2009). Gene set enrichment analysis with topGO. Bioconductor Improv.

[64] Nguyen LT, Schmidt HA, von Haeseler A, Minh BQ (2015). IQ-TREE: a fast and effective stochastic algorithm for estimating maximum-likelihood phylogenies. Mol Biol Evol.

[65] Yang J, Lee SH, Goddard ME, Visscher PM (2011). GCTA: a tool for genome-wide complex trait analysis. Am J Hum Genet.

[66] Danecek P, Auton A, Abecasis G, Albers CA, Banks E, DePristo MA (2011). The variant call format and VCFtools. Bioinformatics..

[67] Slifer SH (2018). PLINK: key functions for data analysis. Curr Protoc Hum Genet.

[68] Ou S, Su W, Liao Y, Chougule K, Agda JRA, Hellinga AJ (2019). Benchmarking transposable element annotation methods for creation of a streamlined, comprehensive pipeline. Genome Biol.

[69] Qin P, Lu H, Du H, Wang H, Chen W, Chen Z, He Q, Ou S, Zhang H, Li X, Li X, Li Y, Liao Y, Gao Q, Tu B, Yuan H, Ma B, Wang Y, Qian Y, Fan S, Li W, Wang J, He M, Yin J, Li T, Jiang N, Chen X, Liang C, Li S. Rice Resource Center database. https://ricerc.sicau.edu.cn/RiceRC/download/downloadBefore. Accessed 10 Dec 2021.

[70] Wang J, Yang W, Zhang S, Hu H, Yuan Y, Dong J, Chen L, Ma Y, Yang T, Zhou L, Chen J, Liu B, Li C, Edwards D, Zhao J. A pangenome analysis pipeline provides insights into functional gene identification in rice. Sequence Read Archive: PRJNA630113. https://www.ncbi.nlm.nih.gov/bioproject/PRJNA820969. Accessed 28 Mar 2022. 10.1186/s13059-023-02861-9 PMC9878884 36703158

[71] Wang J, Yang W, Zhang S, Hu H, Yuan Y, Dong J, Chen L, Ma Y, Yang T, Zhou L, Chen J, Liu B, Li C, Edwards D, Zhao J. A pangenome analysis pipeline provides insights into functional gene identification in rice. Sequence Read Archive: PRJNA886102. https://www.ncbi.nlm.nih.gov/bioproject/PRJNA886102. Accessed 1 Oct 2022. 10.1186/s13059-023-02861-9 PMC9878884 36703158

[72] Wang J, Yang W, Zhang S, Hu H, Yuan Y, Dong J, Chen L, Ma Y, Yang T, Zhou L, Chen J, Liu B, Li C, Edwards D, Zhao J. A pangenome analysis pipeline provides insights into functional gene identification in rice. GitHub. https://github.com/wjian8/psvcp_v1.01. Accessed 4 Oct 2022. 10.1186/s13059-023-02861-9 PMC9878884 36703158

[73] Wang J, Yang W, Zhang S, Hu H, Yuan Y, Dong J, Chen L, Ma Y, Yang T, Zhou L, Chen J, Liu B, Li C, Edwards D, Zhao J. A pangenome analysis pipeline provides insights into functional gene identification in rice. Zenodo. https://zenodo.org/record/7034295. Accessed 14 Jan 2023. 10.1186/s13059-023-02861-9 PMC9878884 36703158

[74] Wang J, Yang W, Zhang S, Hu H, Yuan Y, Dong J, Chen L, Ma Y, Yang T, Zhou L, Chen J, Liu B, Li C, Edwards D, Zhao J. A pangenome analysis pipeline provides insights into functional gene identification in rice. OSF. https://osf.io/38gtp. Accessed 30 Sep 2022. 10.1186/s13059-023-02861-9 PMC9878884 36703158

