# Supplementary material for: A pangenome analysis pipeline provides insights into functional gene identification in rice
Source: Genome Biol. 2023 Jan 26;24:19. doi: 10.1186/s13059-023-02861-9 (PMC9878884; doi:10.1186/s13059-023-02861-9)
Supplement: Supplementary file 1 — Additional file 1: Supplemental figures 1-28. [file 13059_2023_2861_MOESM1_ESM.pptx]

## Slide 1
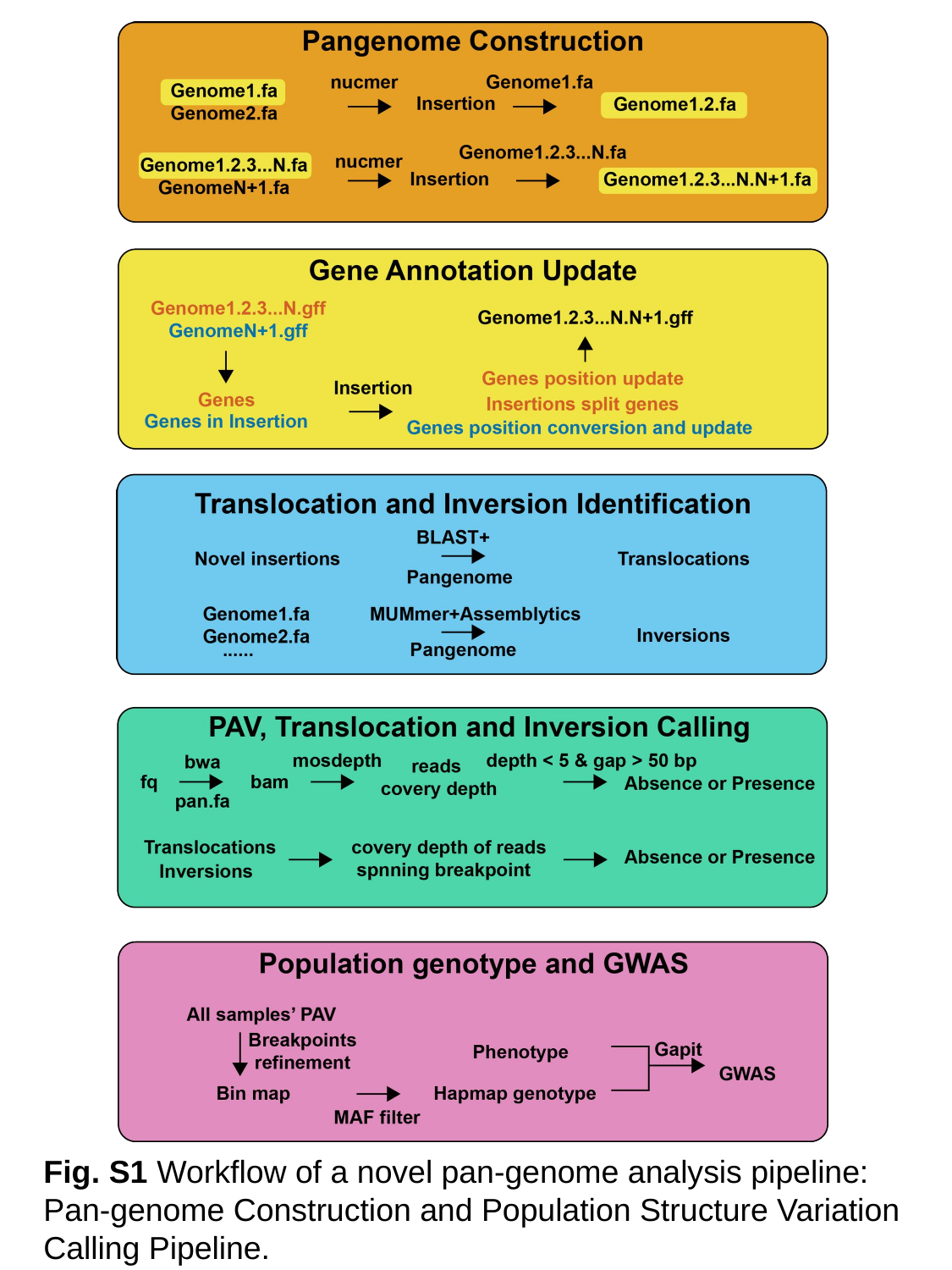

Fig. S1 Workflow of a novel pan-genome analysis pipeline: Pan-genome Construction and Population Structure Variation Calling Pipeline.

## Slide 2
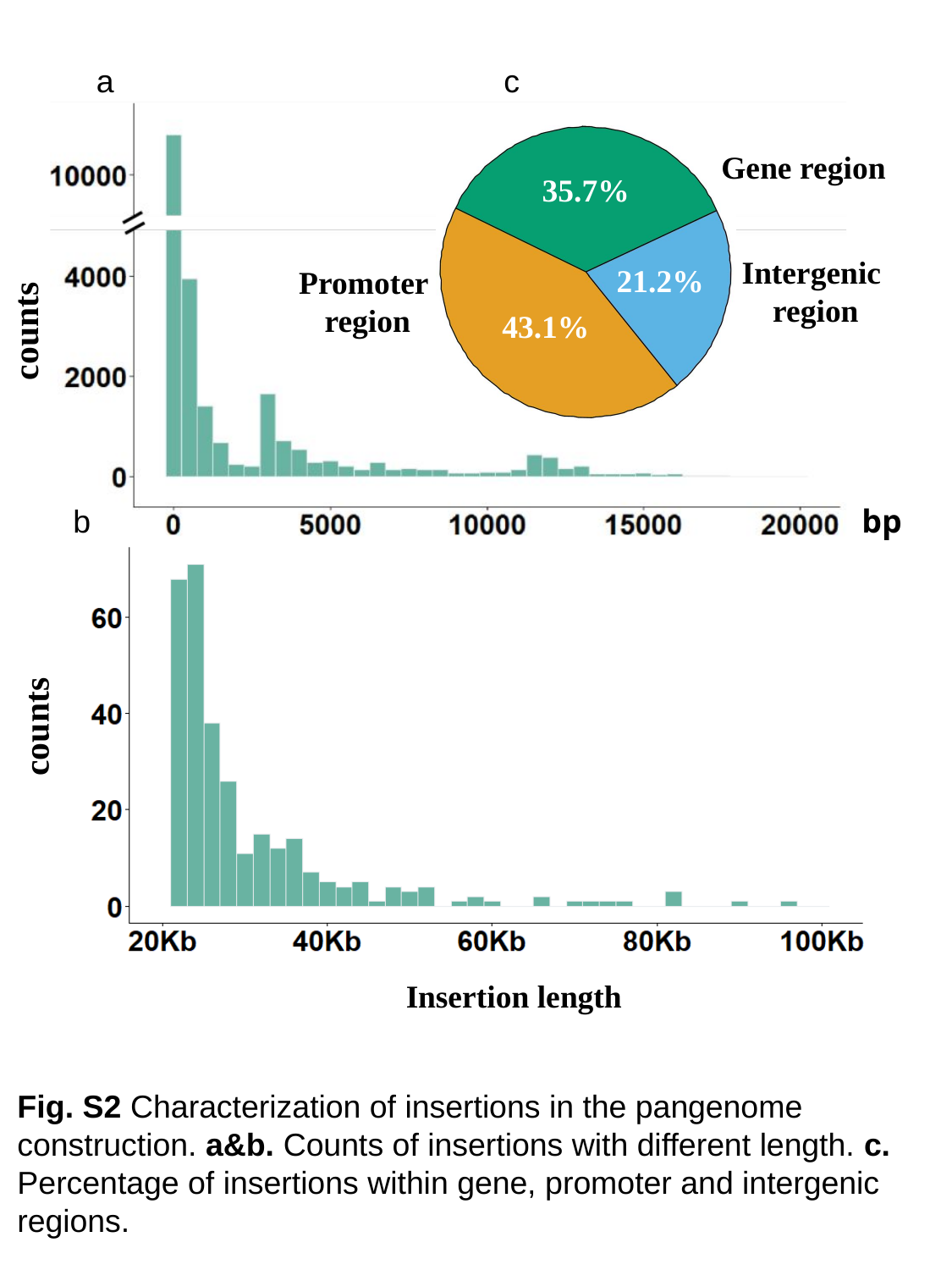

a
c
counts
bp
counts
Insertion length
35.7%
21.2%
43.1%
Gene region
Intergenic
region
Promoter
region
b
Fig. S2 Characterization of insertions in the pangenome construction. a&b. Counts of insertions with different length. c. Percentage of insertions within gene, promoter and intergenic regions.

## Slide 3
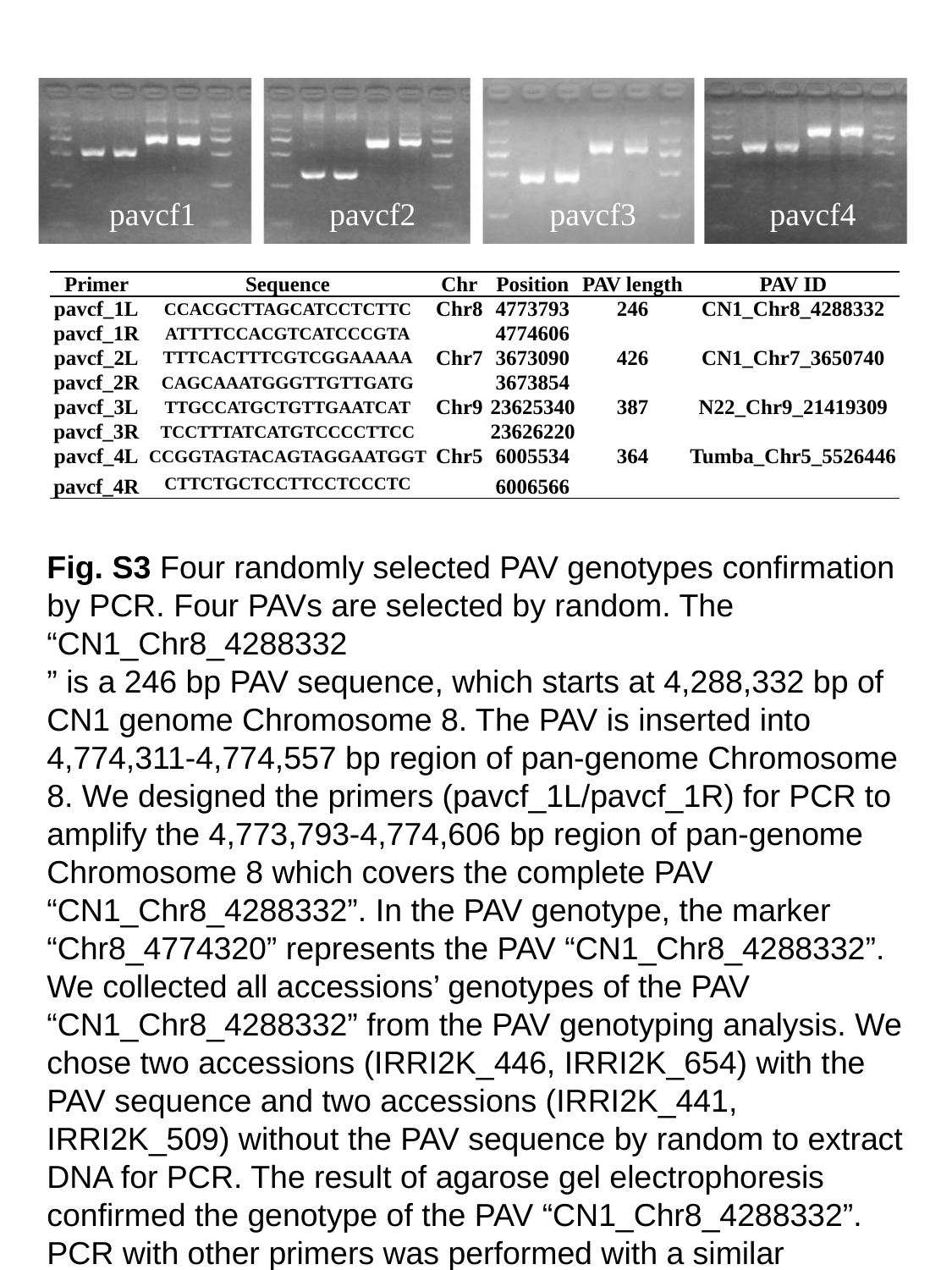

pavcf1
pavcf2
pavcf3
pavcf4
| Primer | Sequence | Chr | Position | PAV length | PAV ID |
| --- | --- | --- | --- | --- | --- |
| pavcf\_1L | CCACGCTTAGCATCCTCTTC | Chr8 | 4773793 | 246 | CN1\_Chr8\_4288332 |
| pavcf\_1R | ATTTTCCACGTCATCCCGTA | | 4774606 | | |
| pavcf\_2L | TTTCACTTTCGTCGGAAAAA | Chr7 | 3673090 | 426 | CN1\_Chr7\_3650740 |
| pavcf\_2R | CAGCAAATGGGTTGTTGATG | | 3673854 | | |
| pavcf\_3L | TTGCCATGCTGTTGAATCAT | Chr9 | 23625340 | 387 | N22\_Chr9\_21419309 |
| pavcf\_3R | TCCTTTATCATGTCCCCTTCC | | 23626220 | | |
| pavcf\_4L | CCGGTAGTACAGTAGGAATGGT | Chr5 | 6005534 | 364 | Tumba\_Chr5\_5526446 |
| pavcf\_4R | CTTCTGCTCCTTCCTCCCTC | | 6006566 | | |
Fig. S3 Four randomly selected PAV genotypes confirmation by PCR. Four PAVs are selected by random. The “CN1_Chr8_4288332
” is a 246 bp PAV sequence, which starts at 4,288,332 bp of CN1 genome Chromosome 8. The PAV is inserted into 4,774,311-4,774,557 bp region of pan-genome Chromosome 8. We designed the primers (pavcf_1L/pavcf_1R) for PCR to amplify the 4,773,793-4,774,606 bp region of pan-genome Chromosome 8 which covers the complete PAV “CN1_Chr8_4288332”. In the PAV genotype, the marker “Chr8_4774320” represents the PAV “CN1_Chr8_4288332”. We collected all accessions’ genotypes of the PAV “CN1_Chr8_4288332” from the PAV genotyping analysis. We chose two accessions (IRRI2K_446, IRRI2K_654) with the PAV sequence and two accessions (IRRI2K_441, IRRI2K_509) without the PAV sequence by random to extract DNA for PCR. The result of agarose gel electrophoresis confirmed the genotype of the PAV “CN1_Chr8_4288332”. PCR with other primers was performed with a similar process.

## Slide 4
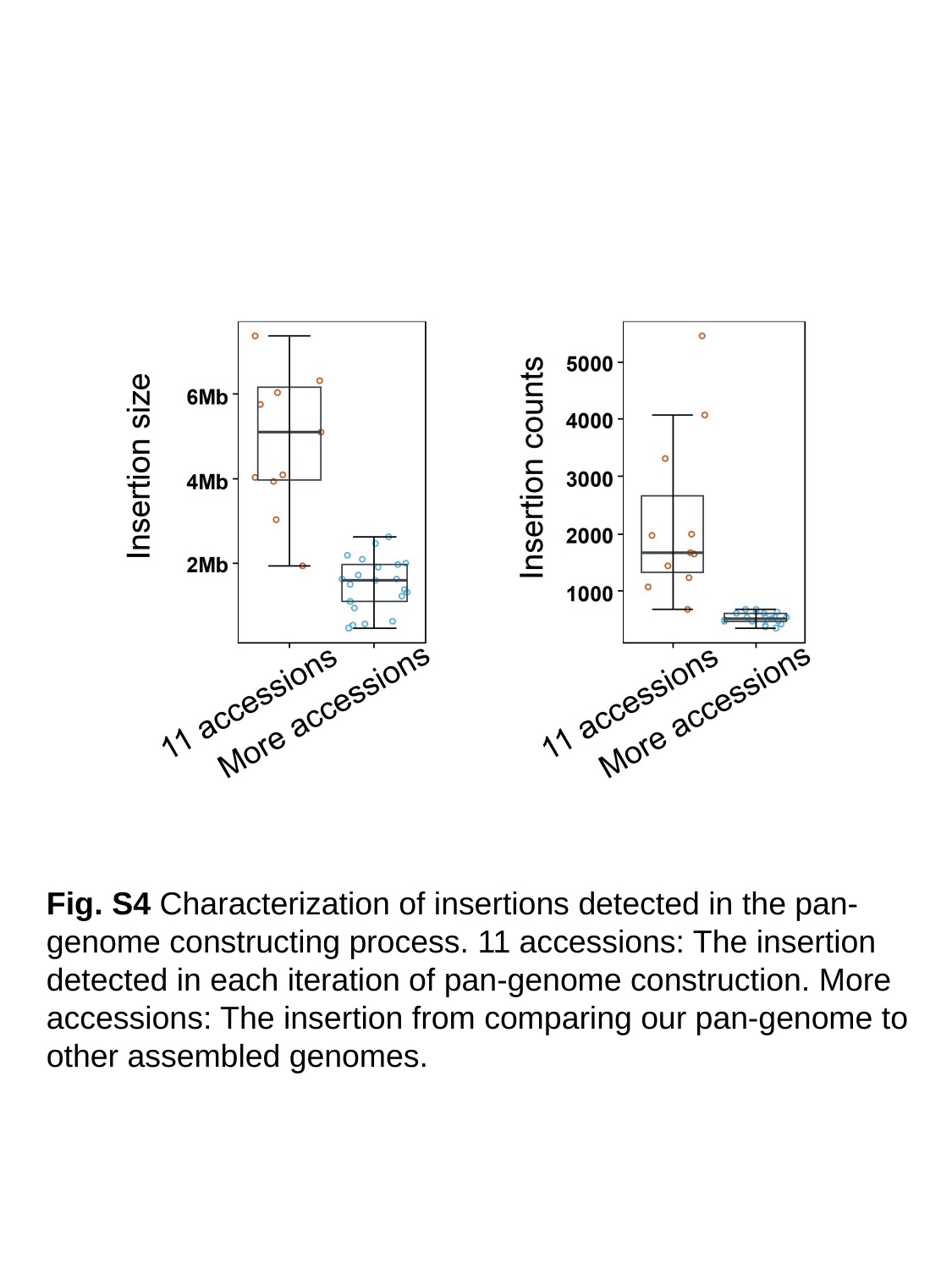

Fig. S4 Characterization of insertions detected in the pan-genome constructing process. 11 accessions: The insertion detected in each iteration of pan-genome construction. More accessions: The insertion from comparing our pan-genome to other assembled genomes.

## Slide 5
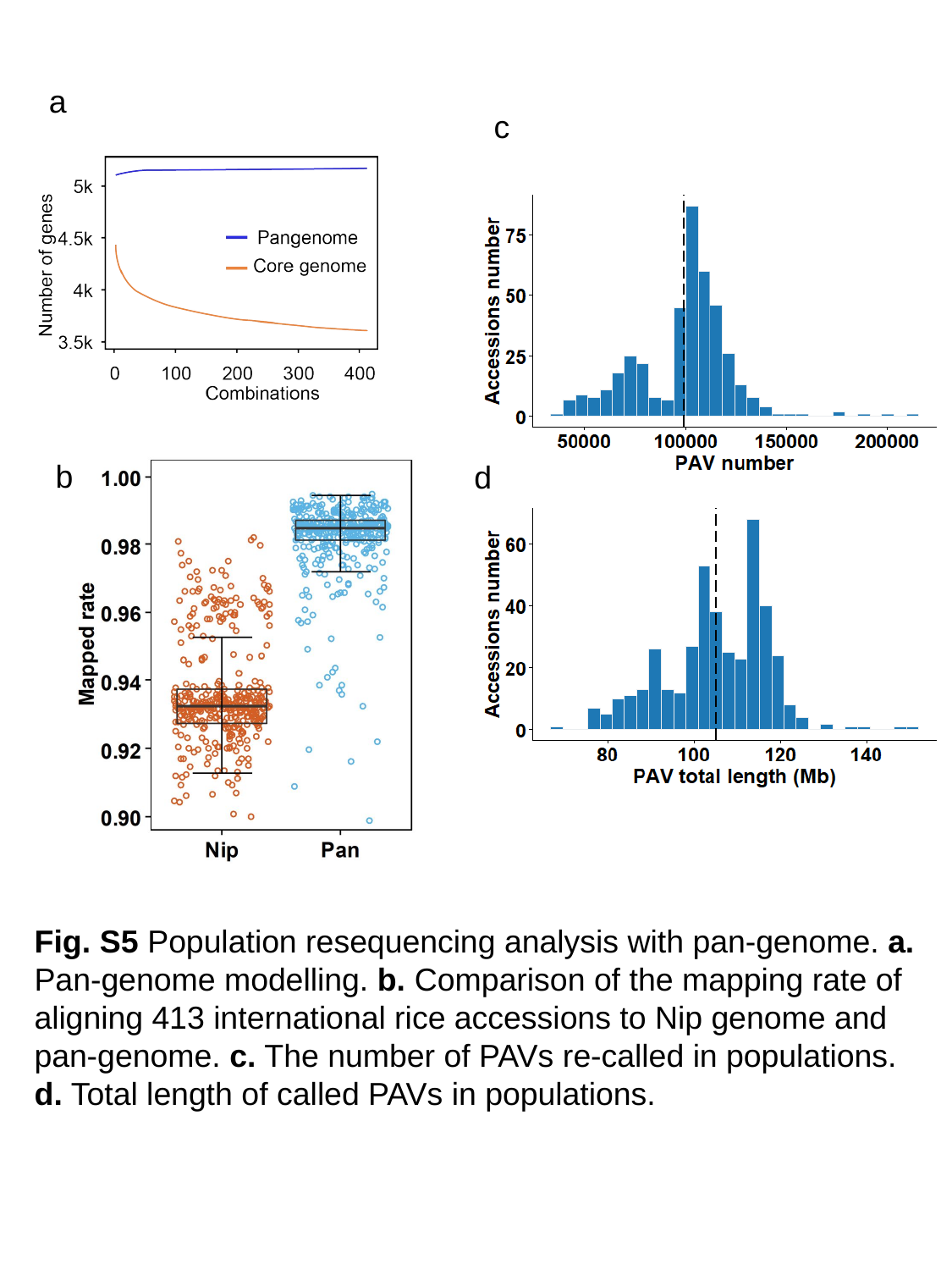

a
c
b
d
Fig. S5 Population resequencing analysis with pan-genome. a. Pan-genome modelling. b. Comparison of the mapping rate of aligning 413 international rice accessions to Nip genome and pan-genome. c. The number of PAVs re-called in populations. d. Total length of called PAVs in populations.

## Slide 6
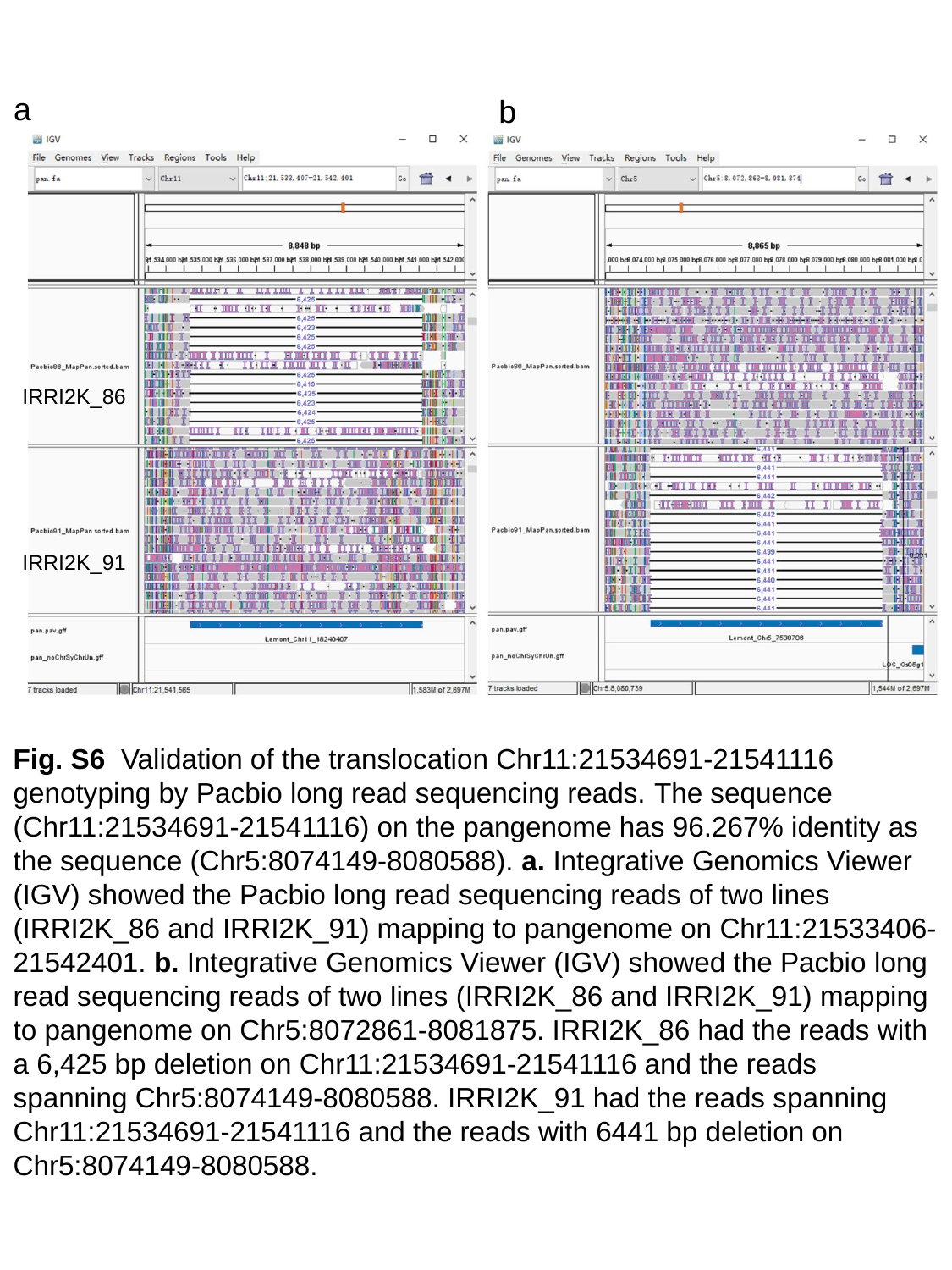

a
b
IRRI2K_86
IRRI2K_91
Fig. S6 Validation of the translocation Chr11:21534691-21541116 genotyping by Pacbio long read sequencing reads. The sequence (Chr11:21534691-21541116) on the pangenome has 96.267% identity as the sequence (Chr5:8074149-8080588). a. Integrative Genomics Viewer (IGV) showed the Pacbio long read sequencing reads of two lines (IRRI2K_86 and IRRI2K_91) mapping to pangenome on Chr11:21533406-21542401. b. Integrative Genomics Viewer (IGV) showed the Pacbio long read sequencing reads of two lines (IRRI2K_86 and IRRI2K_91) mapping to pangenome on Chr5:8072861-8081875. IRRI2K_86 had the reads with a 6,425 bp deletion on Chr11:21534691-21541116 and the reads spanning Chr5:8074149-8080588. IRRI2K_91 had the reads spanning Chr11:21534691-21541116 and the reads with 6441 bp deletion on Chr5:8074149-8080588.

## Slide 7
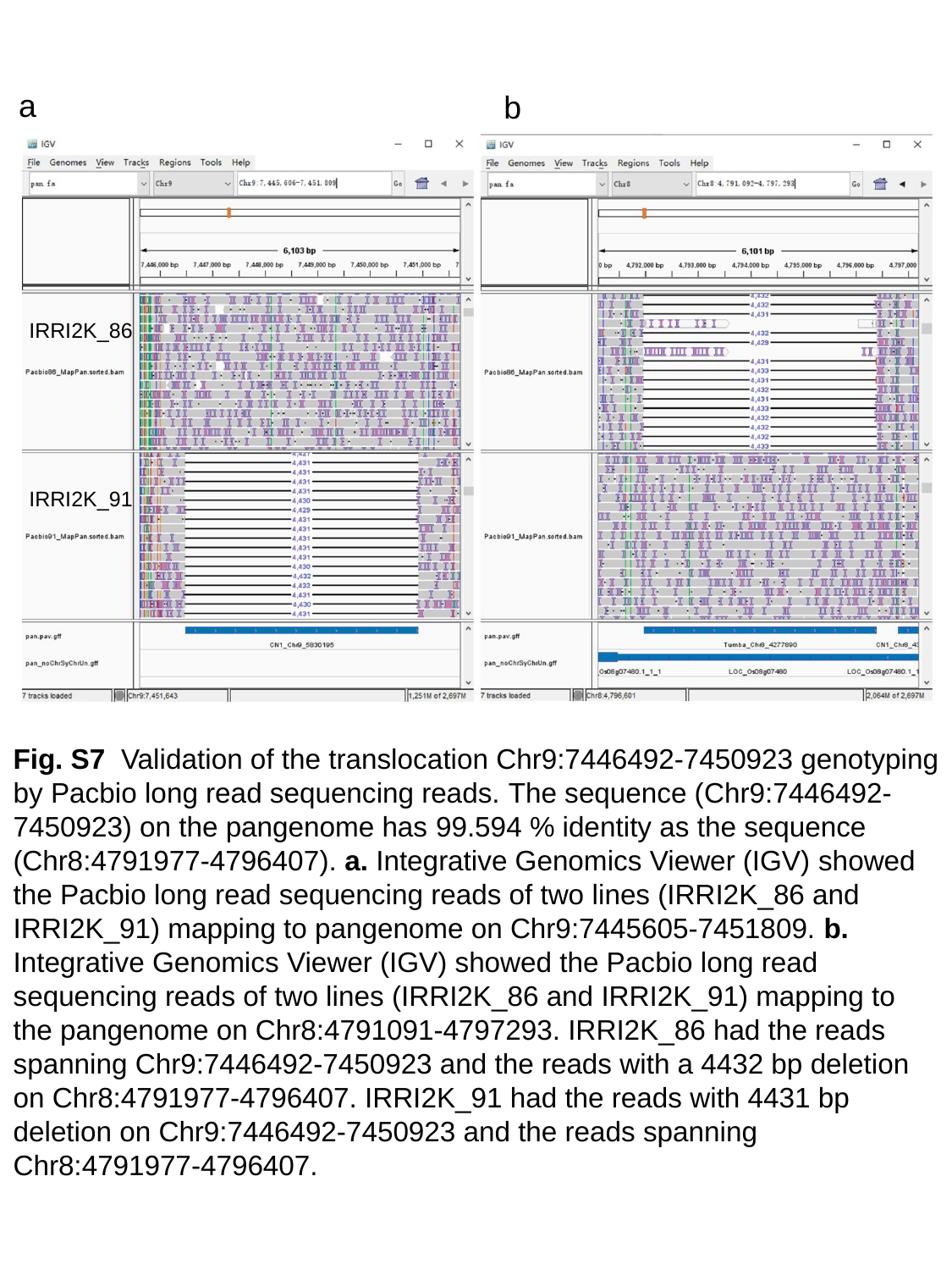

a
b
IRRI2K_86
IRRI2K_91
Fig. S7 Validation of the translocation Chr9:7446492-7450923 genotyping by Pacbio long read sequencing reads. The sequence (Chr9:7446492-7450923) on the pangenome has 99.594 % identity as the sequence (Chr8:4791977-4796407). a. Integrative Genomics Viewer (IGV) showed the Pacbio long read sequencing reads of two lines (IRRI2K_86 and IRRI2K_91) mapping to pangenome on Chr9:7445605-7451809. b. Integrative Genomics Viewer (IGV) showed the Pacbio long read sequencing reads of two lines (IRRI2K_86 and IRRI2K_91) mapping to the pangenome on Chr8:4791091-4797293. IRRI2K_86 had the reads spanning Chr9:7446492-7450923 and the reads with a 4432 bp deletion on Chr8:4791977-4796407. IRRI2K_91 had the reads with 4431 bp deletion on Chr9:7446492-7450923 and the reads spanning Chr8:4791977-4796407.

## Slide 8
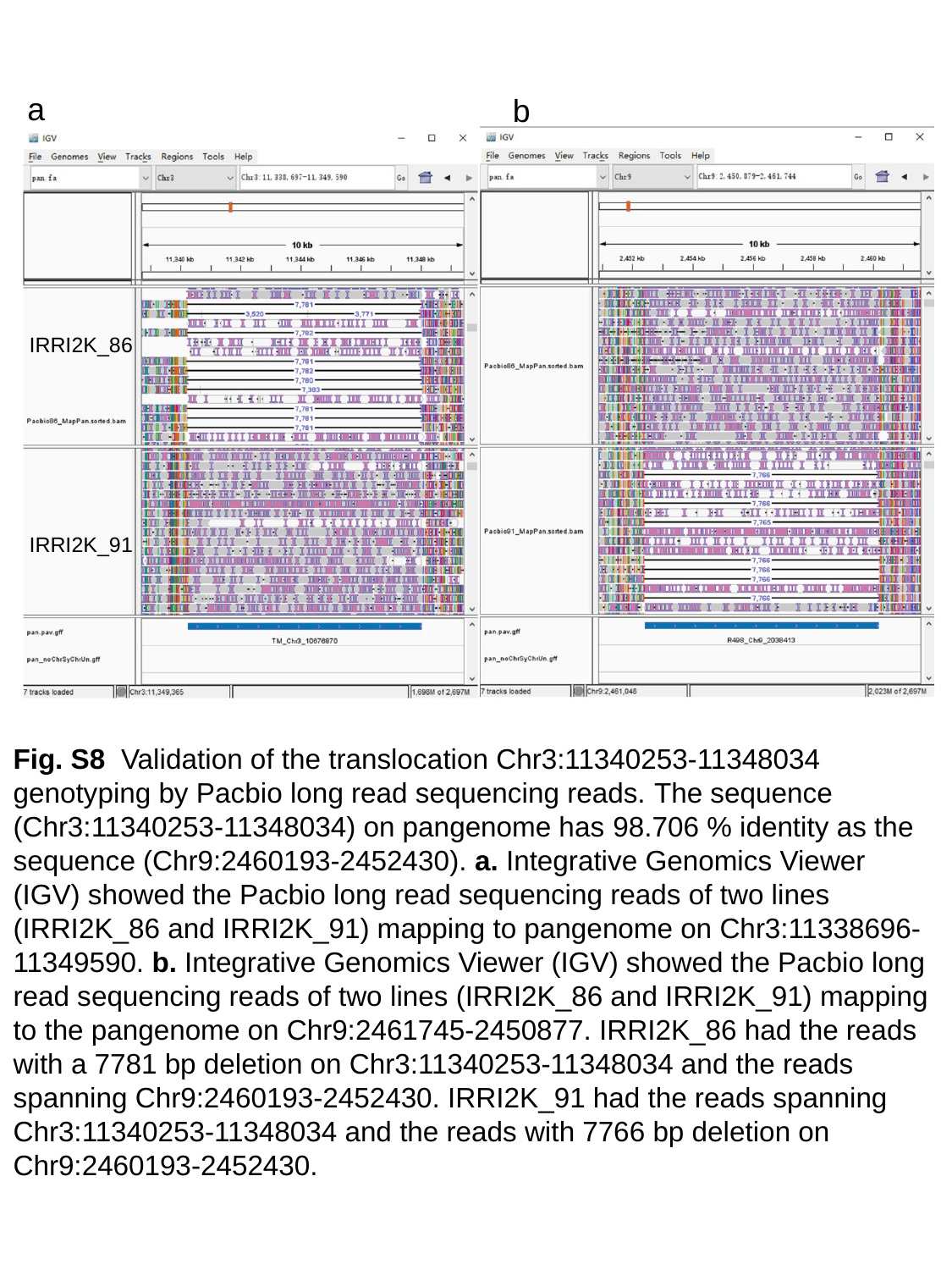

a
b
IRRI2K_86
IRRI2K_91
Fig. S8 Validation of the translocation Chr3:11340253-11348034 genotyping by Pacbio long read sequencing reads. The sequence (Chr3:11340253-11348034) on pangenome has 98.706 % identity as the sequence (Chr9:2460193-2452430). a. Integrative Genomics Viewer (IGV) showed the Pacbio long read sequencing reads of two lines (IRRI2K_86 and IRRI2K_91) mapping to pangenome on Chr3:11338696-11349590. b. Integrative Genomics Viewer (IGV) showed the Pacbio long read sequencing reads of two lines (IRRI2K_86 and IRRI2K_91) mapping to the pangenome on Chr9:2461745-2450877. IRRI2K_86 had the reads with a 7781 bp deletion on Chr3:11340253-11348034 and the reads spanning Chr9:2460193-2452430. IRRI2K_91 had the reads spanning Chr3:11340253-11348034 and the reads with 7766 bp deletion on Chr9:2460193-2452430.

## Slide 9
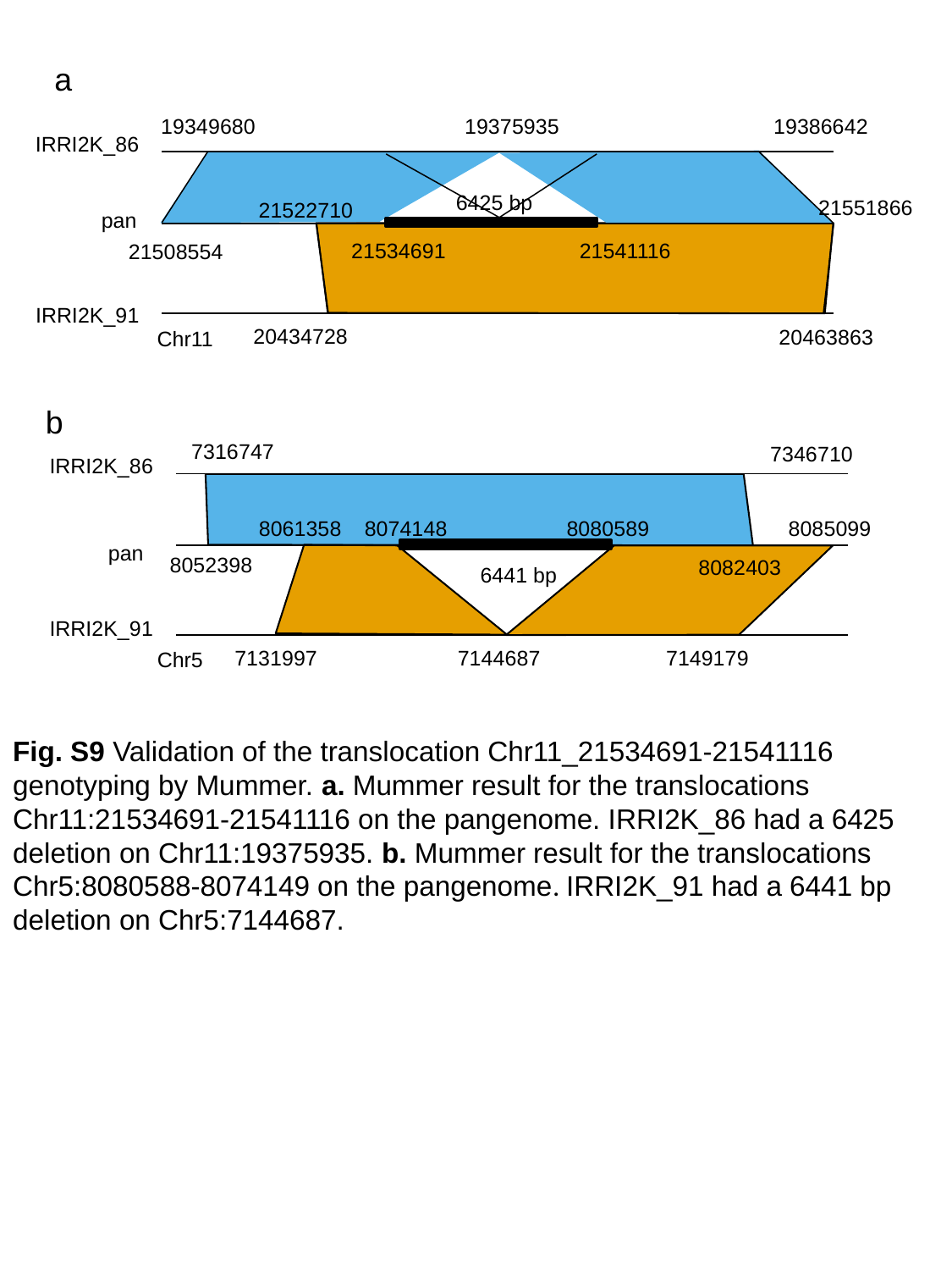

a
19349680
19375935
19386642
IRRI2K_86
6425 bp
21551866
21522710
pan
21534691
21541116
21508554
IRRI2K_91
20434728
20463863
Chr11
b
7316747
7346710
IRRI2K_86
pan
IRRI2K_91
8061358
8074148
8080589
8085099
8052398
8082403
6441 bp
7131997
7144687
7149179
Chr5
Fig. S9 Validation of the translocation Chr11_21534691-21541116 genotyping by Mummer. a. Mummer result for the translocations Chr11:21534691-21541116 on the pangenome. IRRI2K_86 had a 6425 deletion on Chr11:19375935. b. Mummer result for the translocations Chr5:8080588-8074149 on the pangenome. IRRI2K_91 had a 6441 bp deletion on Chr5:7144687.

## Slide 10
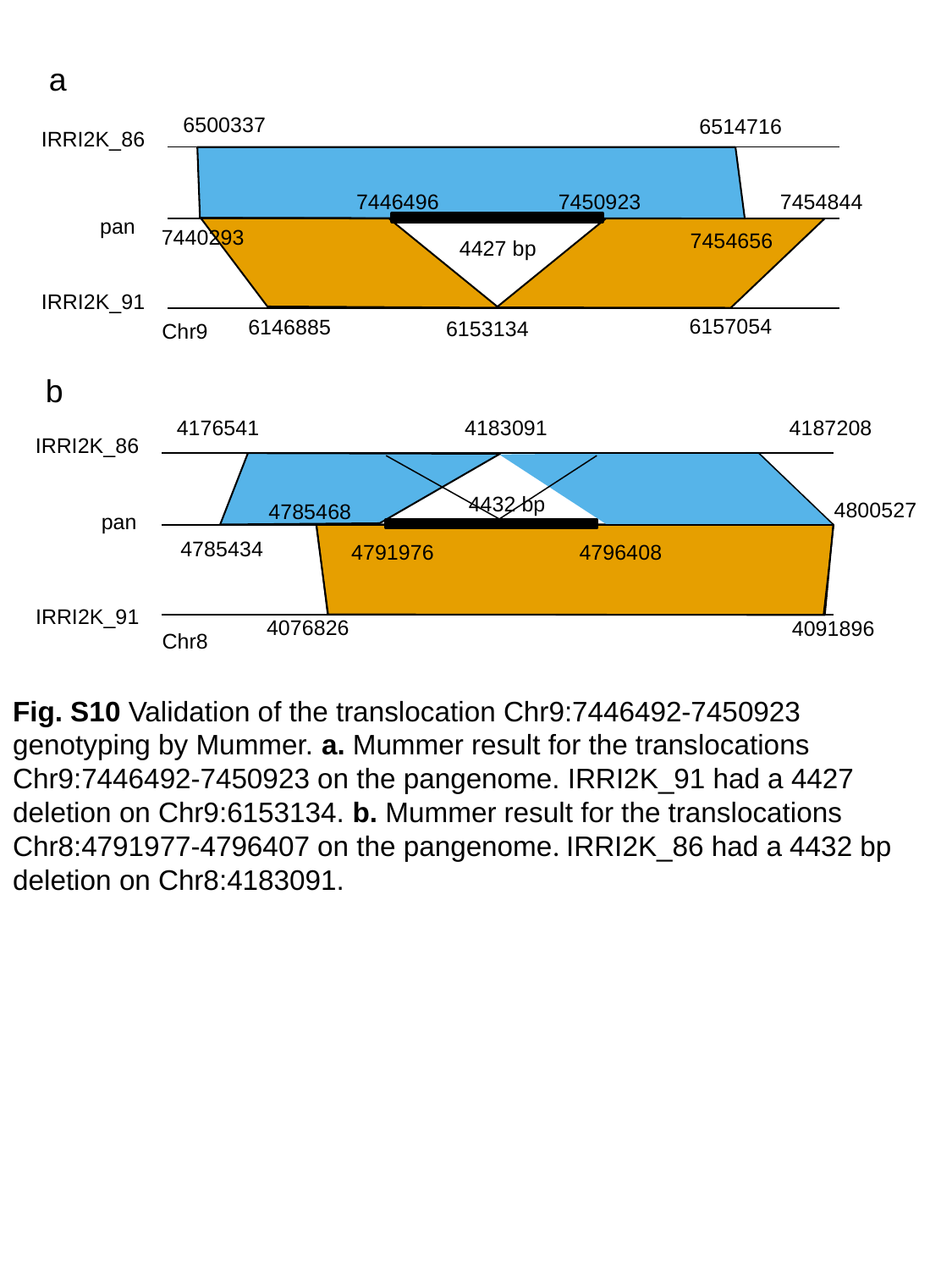

a
6500337
6514716
IRRI2K_86
pan
IRRI2K_91
7446496
7450923
7454844
7440293
7454656
4427 bp
6157054
6146885
6153134
Chr9
b
4176541
4183091
4187208
IRRI2K_86
4432 bp
4800527
4785468
pan
4785434
4791976
4796408
IRRI2K_91
4076826
4091896
Chr8
Fig. S10 Validation of the translocation Chr9:7446492-7450923 genotyping by Mummer. a. Mummer result for the translocations Chr9:7446492-7450923 on the pangenome. IRRI2K_91 had a 4427 deletion on Chr9:6153134. b. Mummer result for the translocations Chr8:4791977-4796407 on the pangenome. IRRI2K_86 had a 4432 bp deletion on Chr8:4183091.

## Slide 11
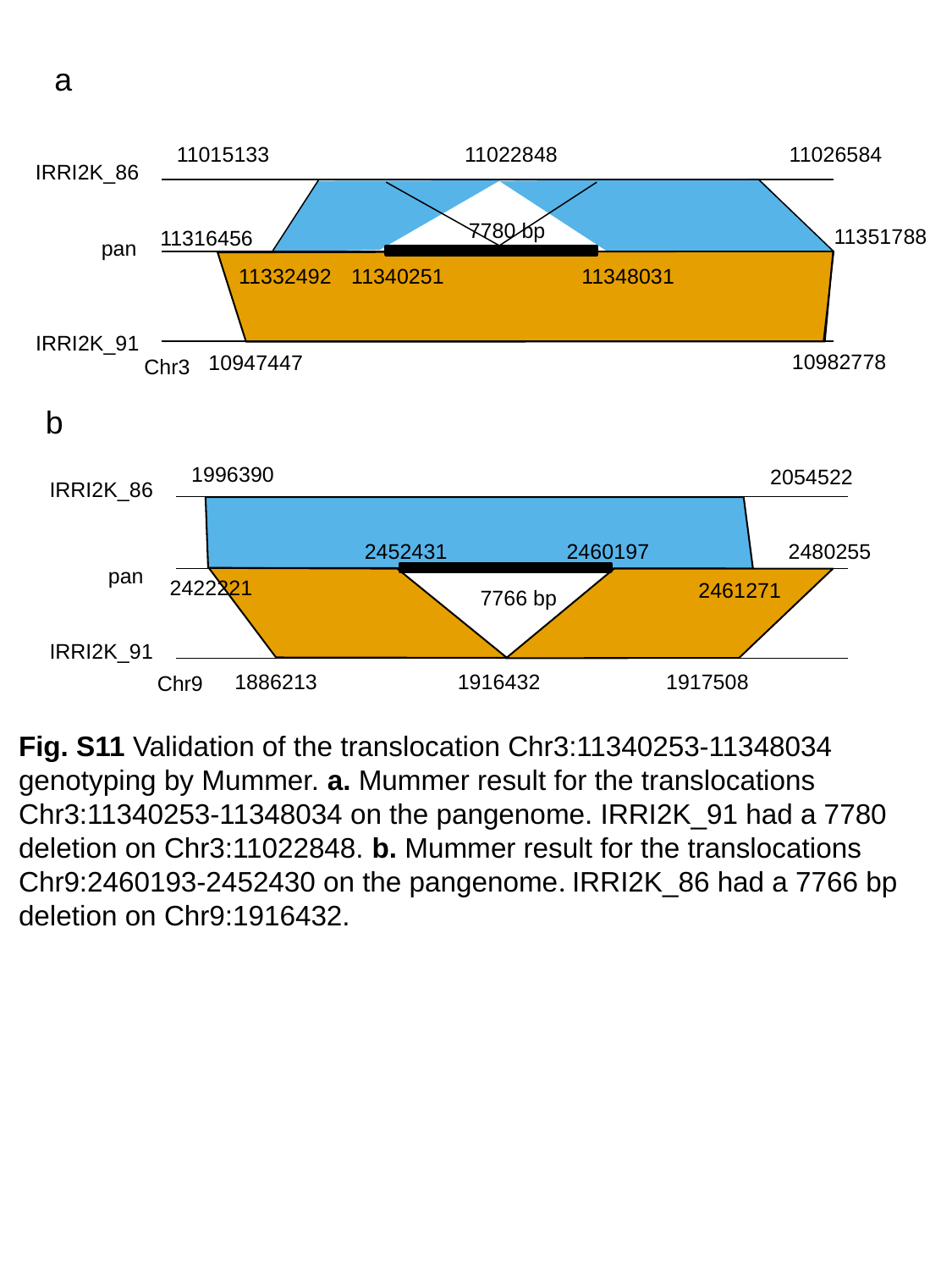

a
11015133
11022848
11026584
IRRI2K_86
7780 bp
11351788
11316456
pan
11332492
11340251
11348031
IRRI2K_91
10982778
10947447
Chr3
b
1996390
2054522
IRRI2K_86
pan
IRRI2K_91
2452431
2460197
2480255
2422221
2461271
7766 bp
1886213
1916432
1917508
Chr9
Fig. S11 Validation of the translocation Chr3:11340253-11348034 genotyping by Mummer. a. Mummer result for the translocations Chr3:11340253-11348034 on the pangenome. IRRI2K_91 had a 7780 deletion on Chr3:11022848. b. Mummer result for the translocations Chr9:2460193-2452430 on the pangenome. IRRI2K_86 had a 7766 bp deletion on Chr9:1916432.

## Slide 12
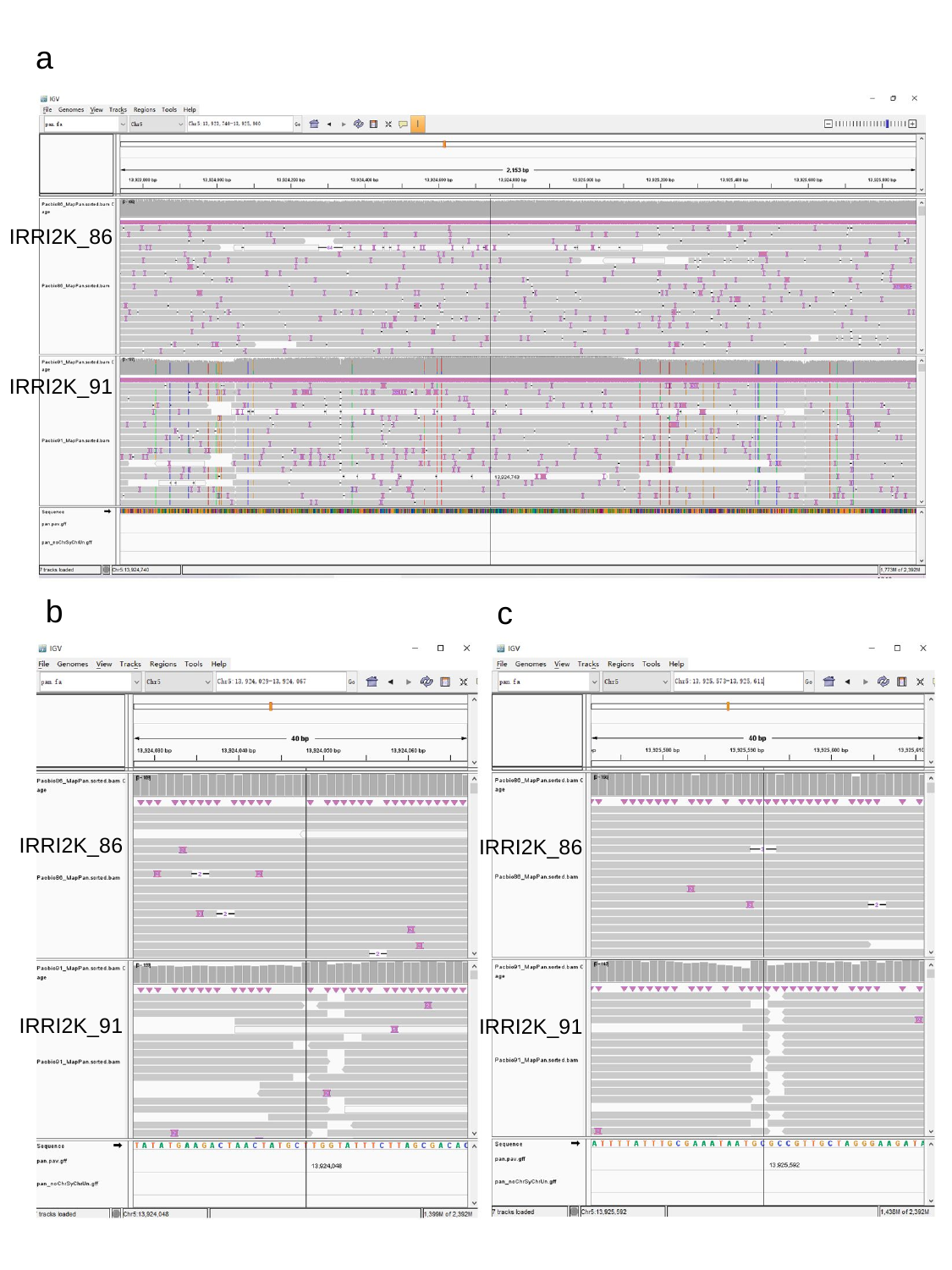

a
IRRI2K_86
IRRI2K_91
b
c
IRRI2K_86
IRRI2K_86
IRRI2K_91
IRRI2K_91

## Slide 13
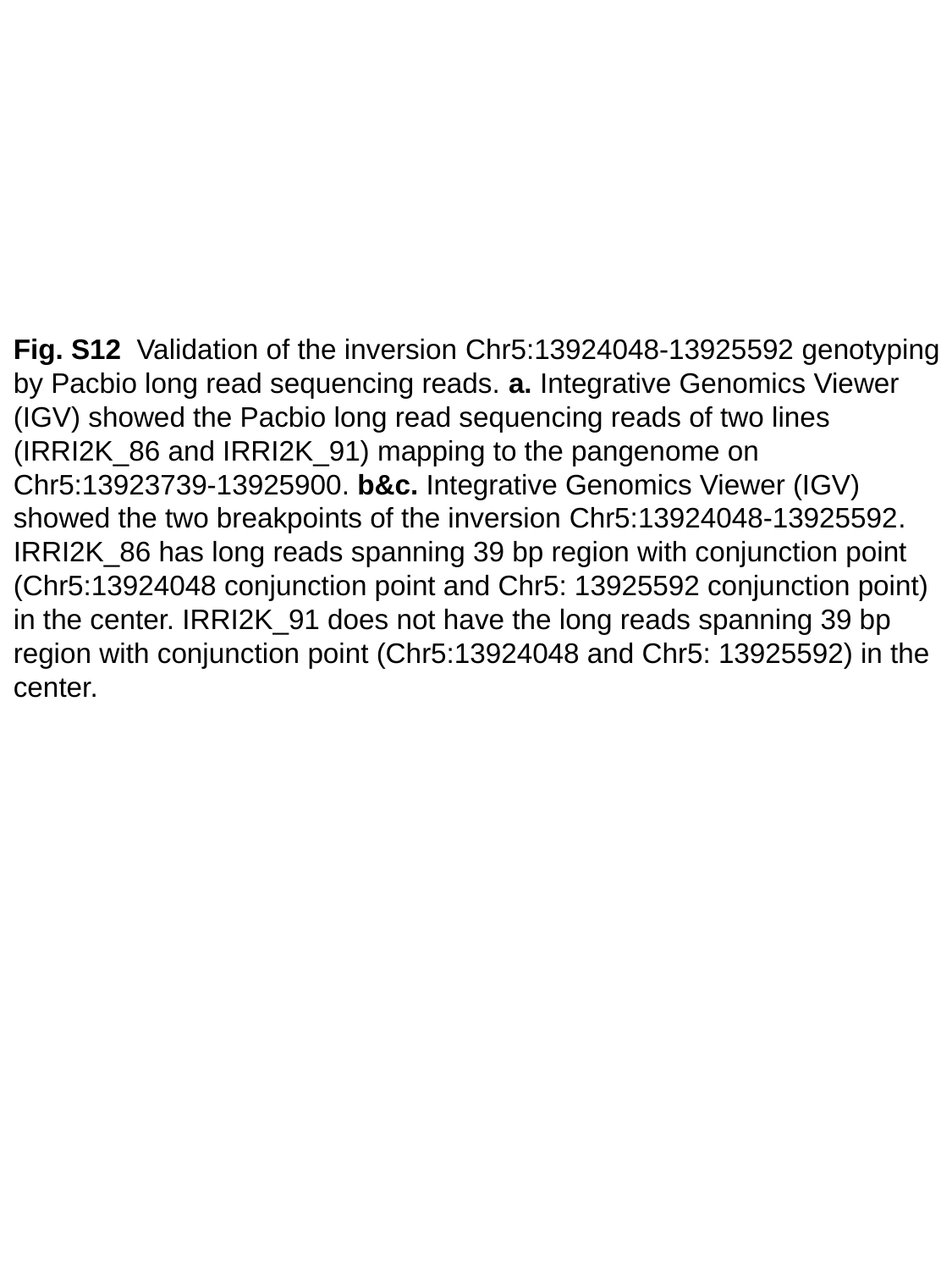

Fig. S12 Validation of the inversion Chr5:13924048-13925592 genotyping by Pacbio long read sequencing reads. a. Integrative Genomics Viewer (IGV) showed the Pacbio long read sequencing reads of two lines (IRRI2K_86 and IRRI2K_91) mapping to the pangenome on Chr5:13923739-13925900. b&c. Integrative Genomics Viewer (IGV) showed the two breakpoints of the inversion Chr5:13924048-13925592. IRRI2K_86 has long reads spanning 39 bp region with conjunction point (Chr5:13924048 conjunction point and Chr5: 13925592 conjunction point) in the center. IRRI2K_91 does not have the long reads spanning 39 bp region with conjunction point (Chr5:13924048 and Chr5: 13925592) in the center.

## Slide 14
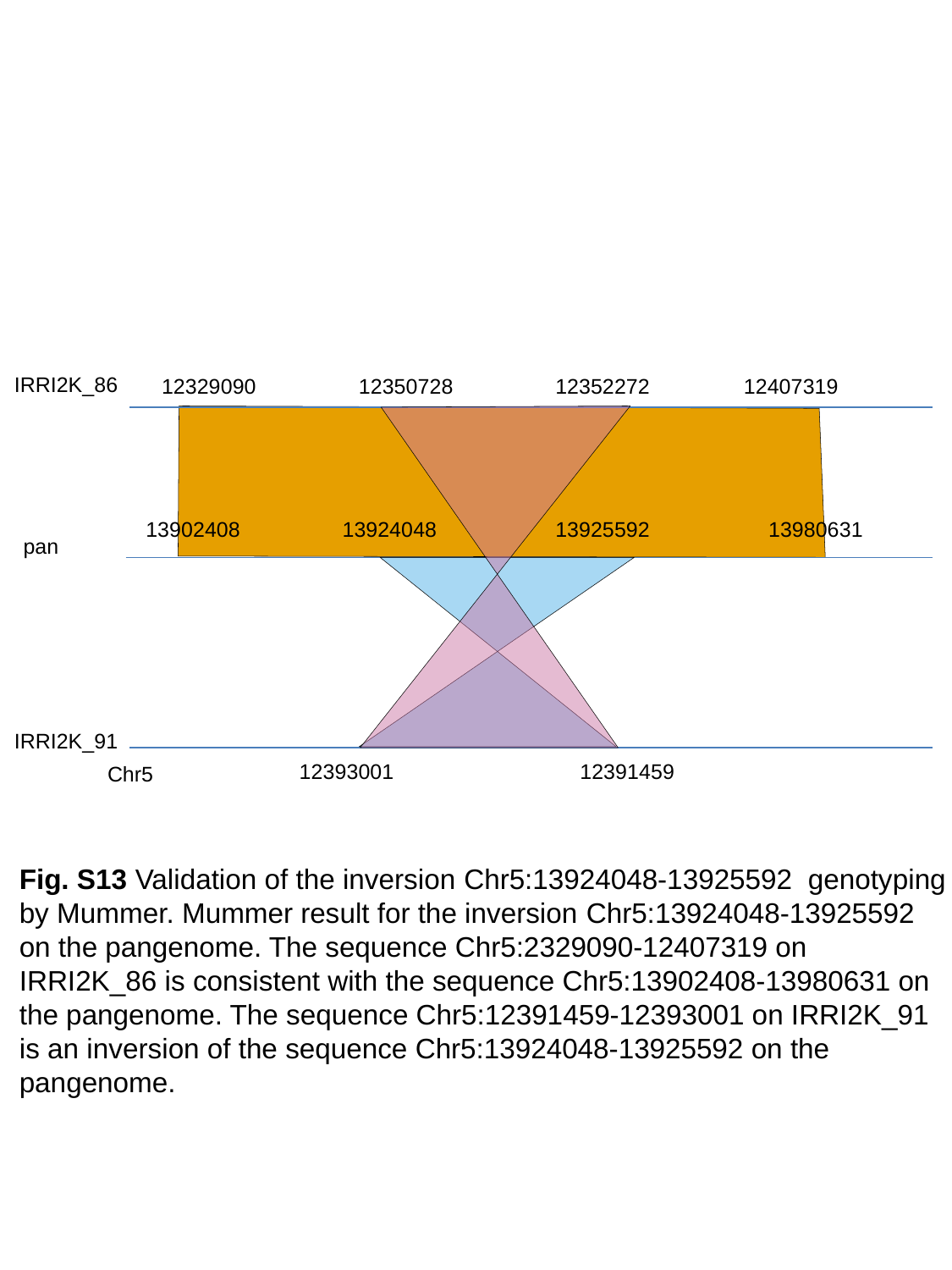

IRRI2K_86
12329090
12350728
12352272
12407319
13902408
13924048
13925592
13980631
pan
IRRI2K_91
12393001
12391459
Chr5
Fig. S13 Validation of the inversion Chr5:13924048-13925592 genotyping by Mummer. Mummer result for the inversion Chr5:13924048-13925592 on the pangenome. The sequence Chr5:2329090-12407319 on IRRI2K_86 is consistent with the sequence Chr5:13902408-13980631 on the pangenome. The sequence Chr5:12391459-12393001 on IRRI2K_91 is an inversion of the sequence Chr5:13924048-13925592 on the pangenome.

## Slide 15
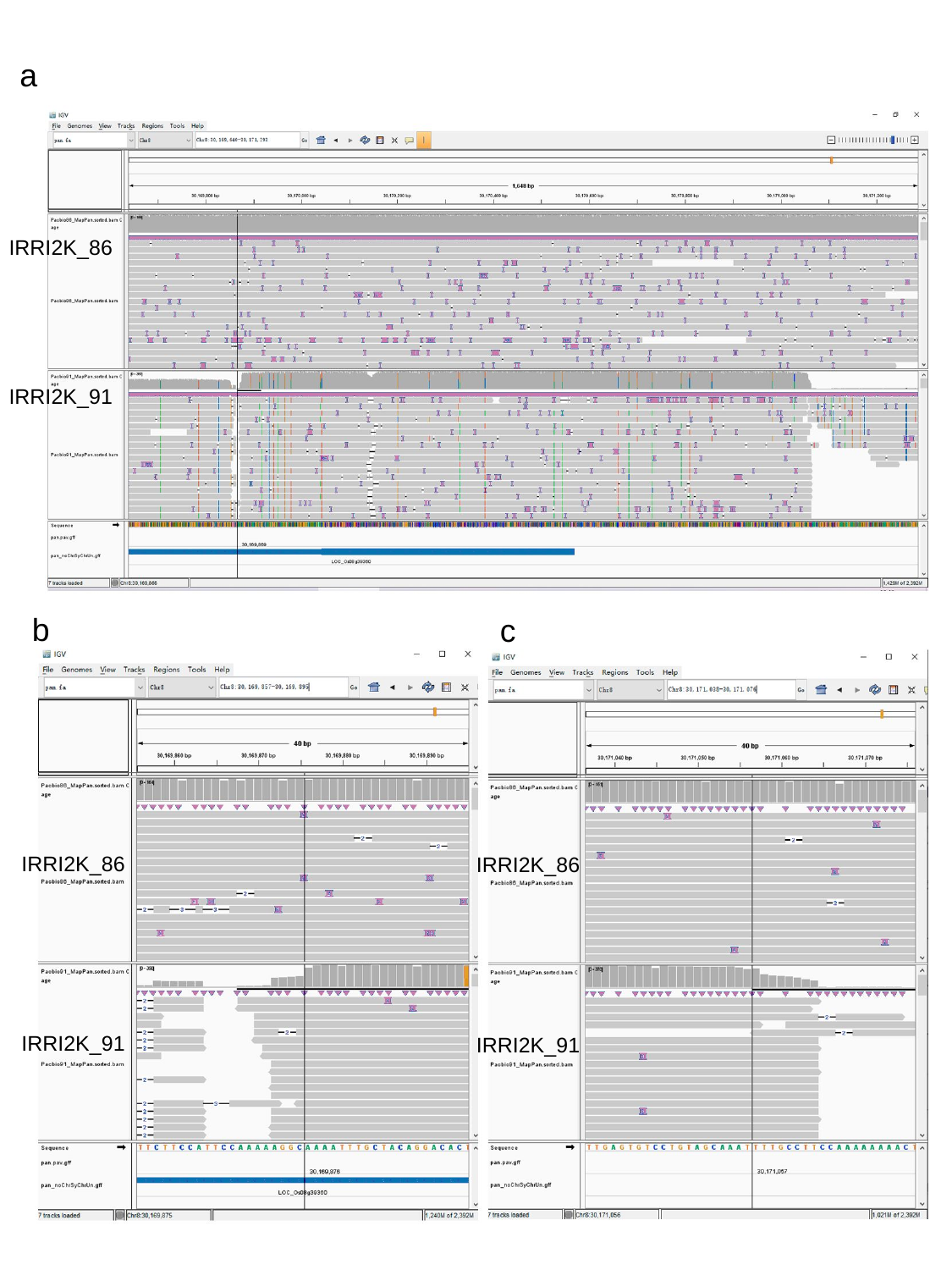

a
IRRI2K_86
IRRI2K_91
b
c
IRRI2K_86
IRRI2K_86
IRRI2K_91
IRRI2K_91

## Slide 16
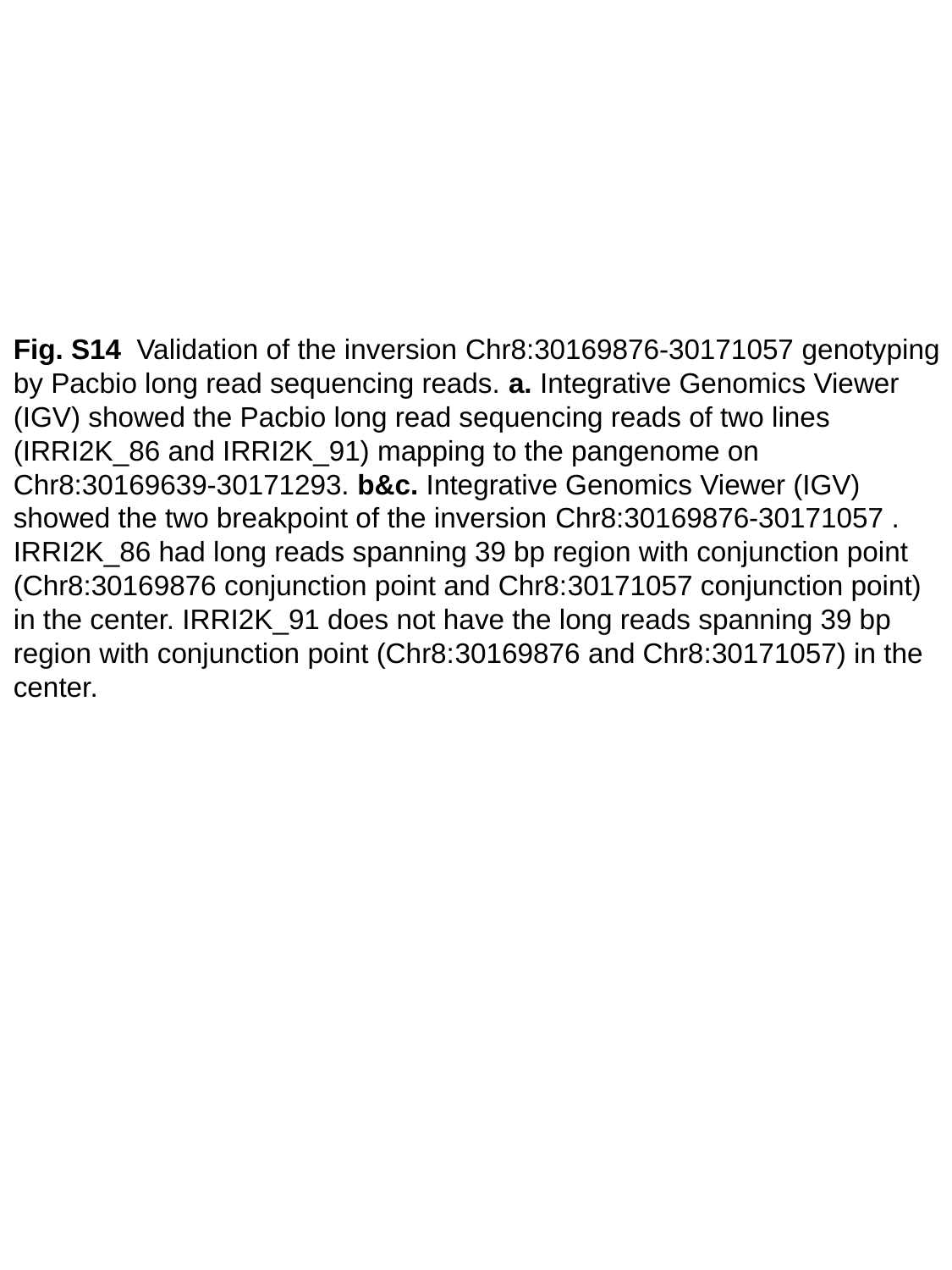

Fig. S14 Validation of the inversion Chr8:30169876-30171057 genotyping by Pacbio long read sequencing reads. a. Integrative Genomics Viewer (IGV) showed the Pacbio long read sequencing reads of two lines (IRRI2K_86 and IRRI2K_91) mapping to the pangenome on Chr8:30169639-30171293. b&c. Integrative Genomics Viewer (IGV) showed the two breakpoint of the inversion Chr8:30169876-30171057 . IRRI2K_86 had long reads spanning 39 bp region with conjunction point (Chr8:30169876 conjunction point and Chr8:30171057 conjunction point) in the center. IRRI2K_91 does not have the long reads spanning 39 bp region with conjunction point (Chr8:30169876 and Chr8:30171057) in the center.

## Slide 17
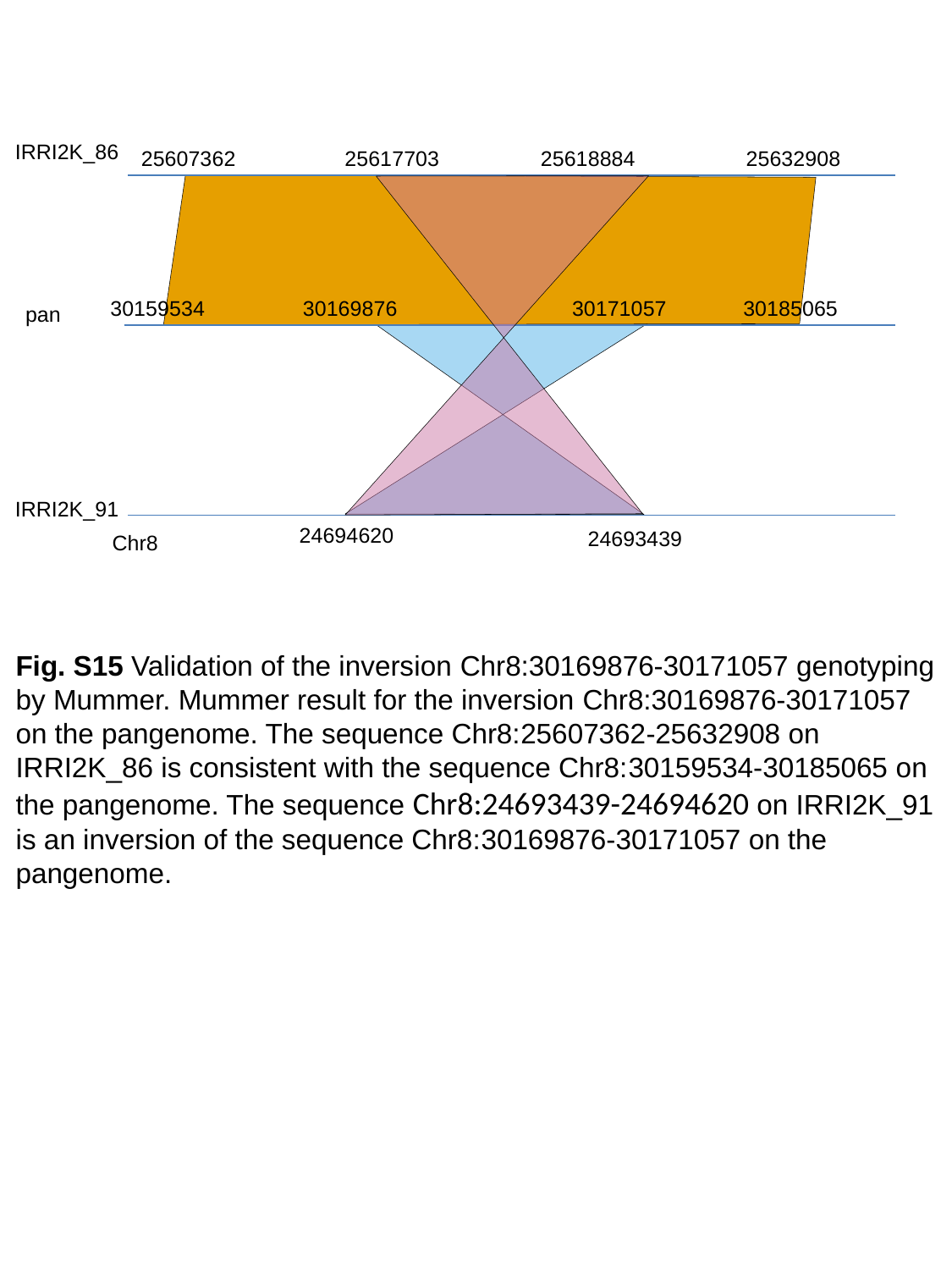

IRRI2K_86
25607362
25617703
25618884
25632908
30159534
30169876
30171057
30185065
pan
IRRI2K_91
24694620
24693439
Chr8
Fig. S15 Validation of the inversion Chr8:30169876-30171057 genotyping by Mummer. Mummer result for the inversion Chr8:30169876-30171057 on the pangenome. The sequence Chr8:25607362-25632908 on IRRI2K_86 is consistent with the sequence Chr8:30159534-30185065 on the pangenome. The sequence Chr8:24693439-24694620 on IRRI2K_91 is an inversion of the sequence Chr8:30169876-30171057 on the pangenome.

## Slide 18
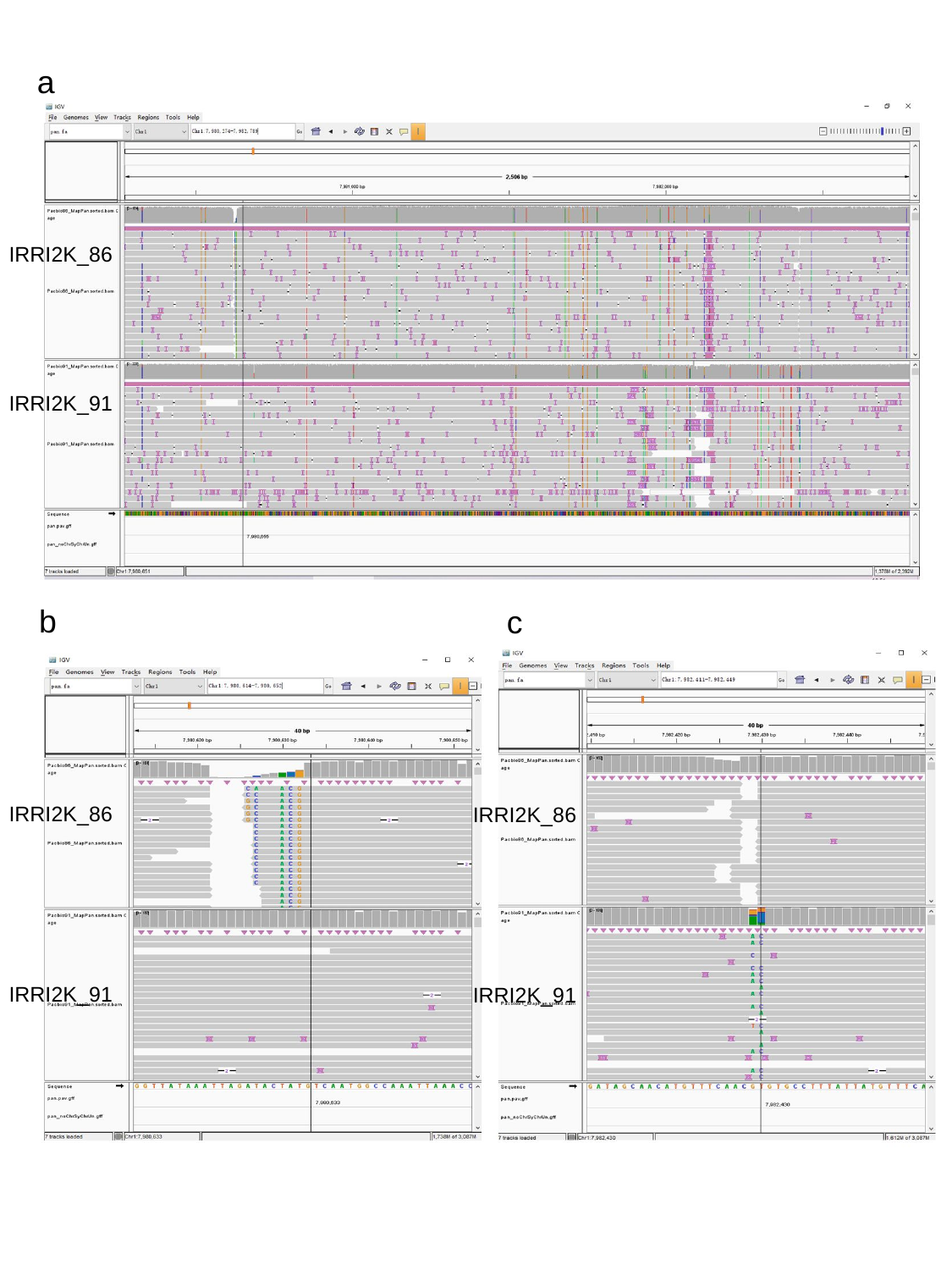

a
IRRI2K_86
IRRI2K_91
b
c
IRRI2K_86
IRRI2K_86
IRRI2K_91
IRRI2K_91

## Slide 19
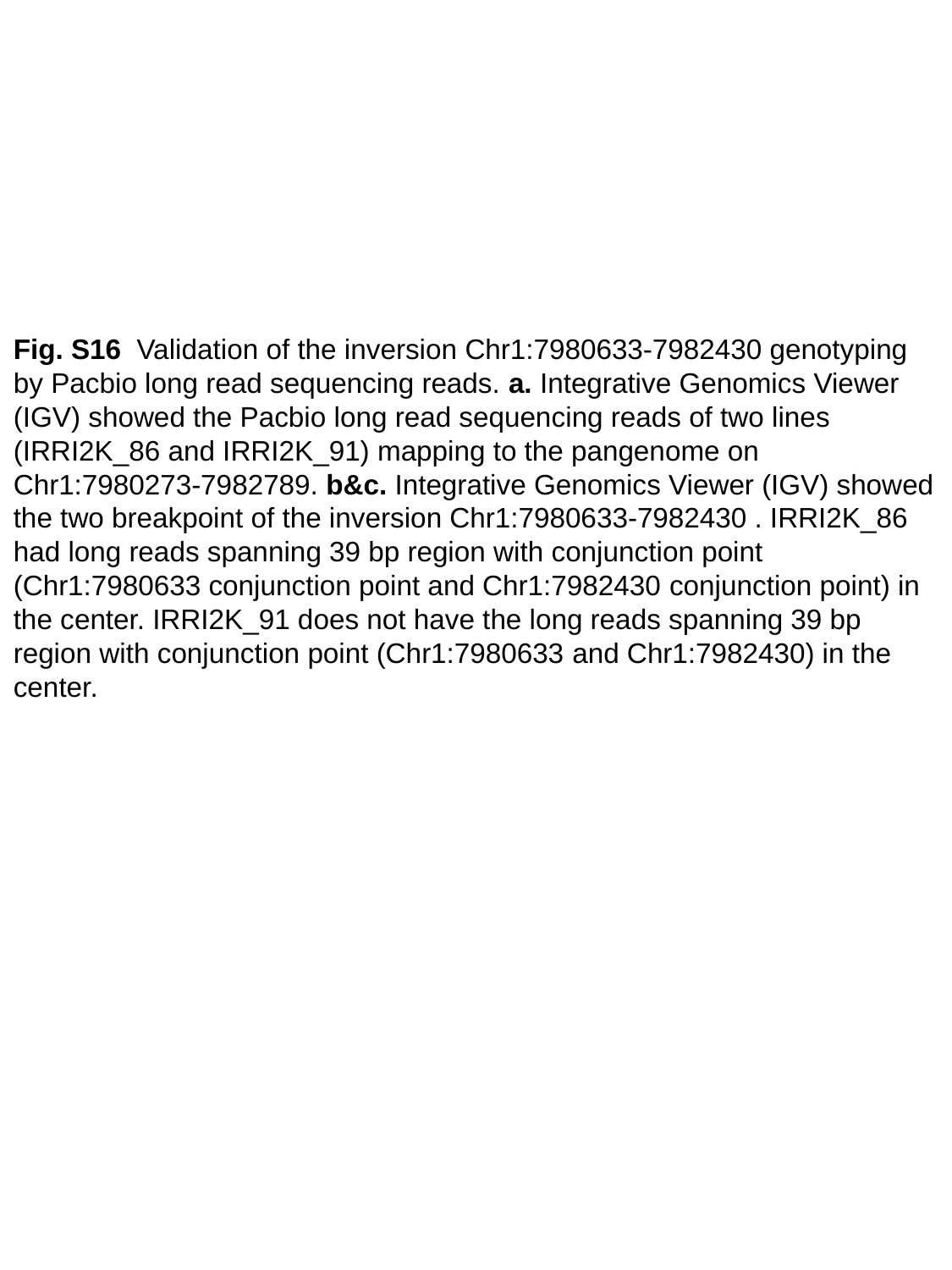

Fig. S16 Validation of the inversion Chr1:7980633-7982430 genotyping by Pacbio long read sequencing reads. a. Integrative Genomics Viewer (IGV) showed the Pacbio long read sequencing reads of two lines (IRRI2K_86 and IRRI2K_91) mapping to the pangenome on Chr1:7980273-7982789. b&c. Integrative Genomics Viewer (IGV) showed the two breakpoint of the inversion Chr1:7980633-7982430 . IRRI2K_86 had long reads spanning 39 bp region with conjunction point (Chr1:7980633 conjunction point and Chr1:7982430 conjunction point) in the center. IRRI2K_91 does not have the long reads spanning 39 bp region with conjunction point (Chr1:7980633 and Chr1:7982430) in the center.

## Slide 20
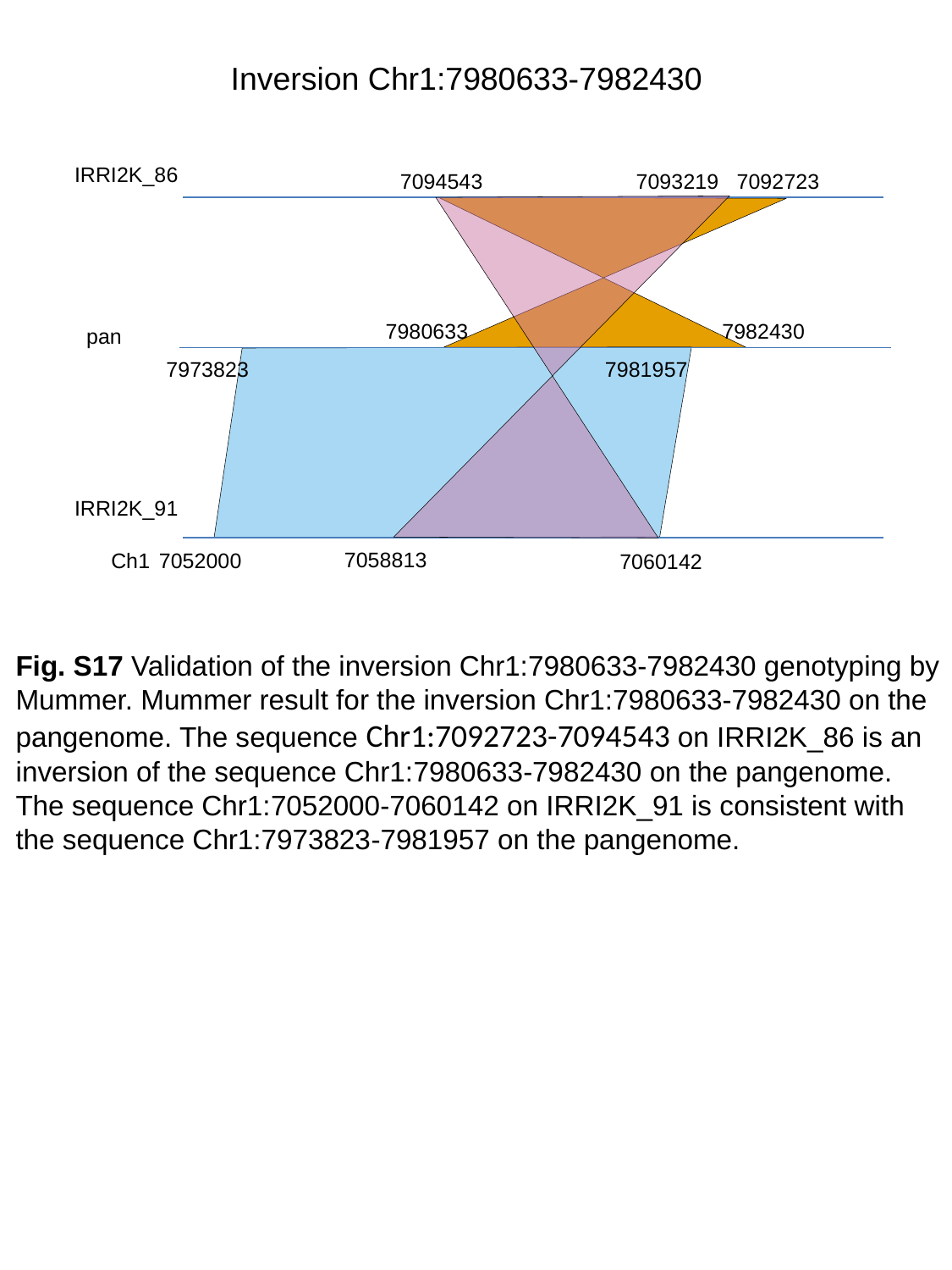

Inversion Chr1:7980633-7982430
IRRI2K_86
7093219
7094543
7092723
7980633
7982430
pan
7973823
7981957
IRRI2K_91
7058813
7052000
7060142
Ch1
Fig. S17 Validation of the inversion Chr1:7980633-7982430 genotyping by Mummer. Mummer result for the inversion Chr1:7980633-7982430 on the pangenome. The sequence Chr1:7092723-7094543 on IRRI2K_86 is an inversion of the sequence Chr1:7980633-7982430 on the pangenome. The sequence Chr1:7052000-7060142 on IRRI2K_91 is consistent with the sequence Chr1:7973823-7981957 on the pangenome.

## Slide 21
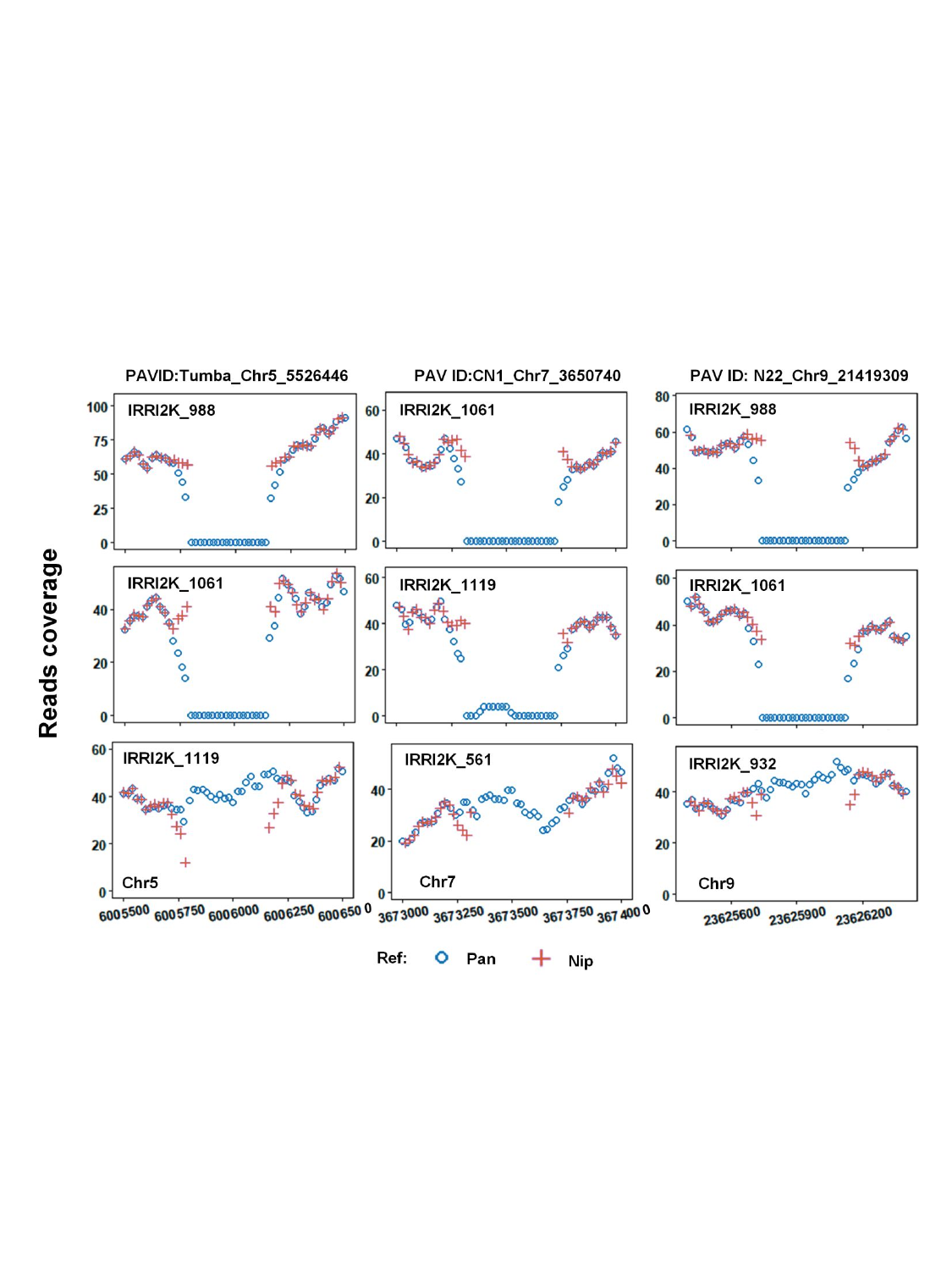

## Slide 22
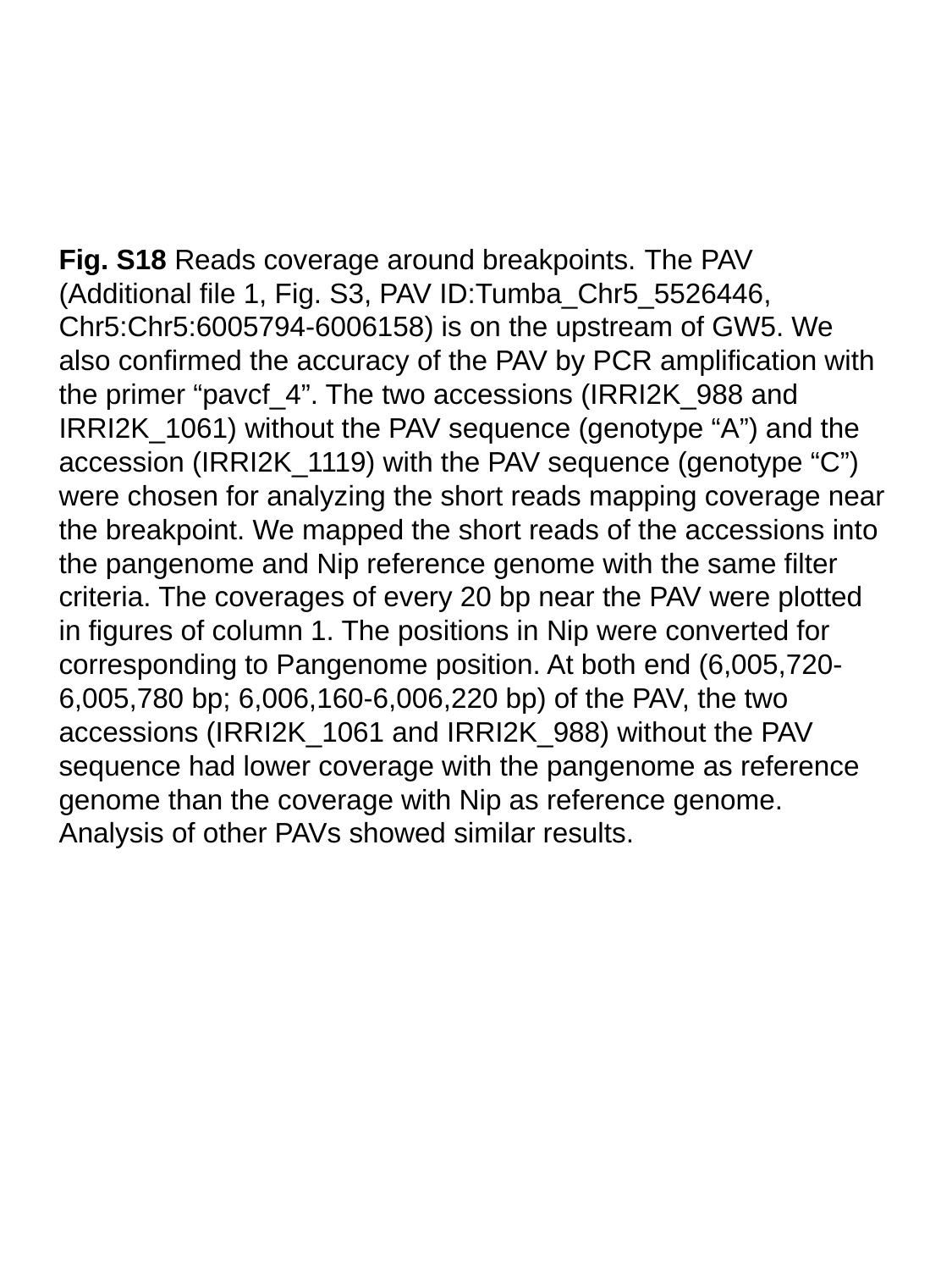

Fig. S18 Reads coverage around breakpoints. The PAV (Additional file 1, Fig. S3, PAV ID:Tumba_Chr5_5526446, Chr5:Chr5:6005794-6006158) is on the upstream of GW5. We also confirmed the accuracy of the PAV by PCR amplification with the primer “pavcf_4”. The two accessions (IRRI2K_988 and IRRI2K_1061) without the PAV sequence (genotype “A”) and the accession (IRRI2K_1119) with the PAV sequence (genotype “C”) were chosen for analyzing the short reads mapping coverage near the breakpoint. We mapped the short reads of the accessions into the pangenome and Nip reference genome with the same filter criteria. The coverages of every 20 bp near the PAV were plotted in figures of column 1. The positions in Nip were converted for corresponding to Pangenome position. At both end (6,005,720-6,005,780 bp; 6,006,160-6,006,220 bp) of the PAV, the two accessions (IRRI2K_1061 and IRRI2K_988) without the PAV sequence had lower coverage with the pangenome as reference genome than the coverage with Nip as reference genome. Analysis of other PAVs showed similar results.

## Slide 23
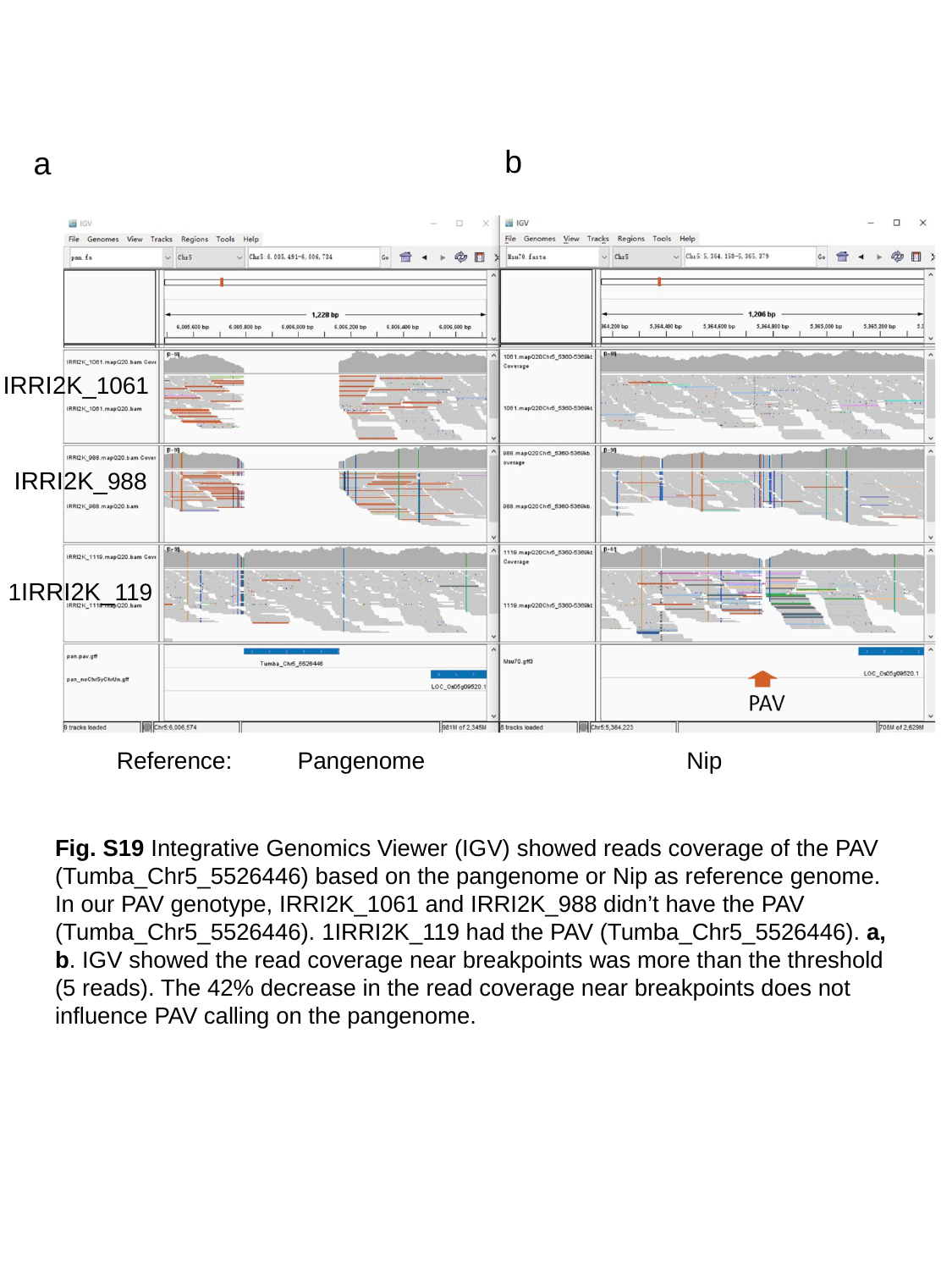

b
a
IRRI2K_1061
IRRI2K_988
1IRRI2K_119
Reference: Pangenome Nip
# Fig. S19 Integrative Genomics Viewer (IGV) showed reads coverage of the PAV (Tumba_Chr5_5526446) based on the pangenome or Nip as reference genome. In our PAV genotype, IRRI2K_1061 and IRRI2K_988 didn’t have the PAV (Tumba_Chr5_5526446). 1IRRI2K_119 had the PAV (Tumba_Chr5_5526446). a, b. IGV showed the read coverage near breakpoints was more than the threshold (5 reads). The 42% decrease in the read coverage near breakpoints does not influence PAV calling on the pangenome.

## Slide 24
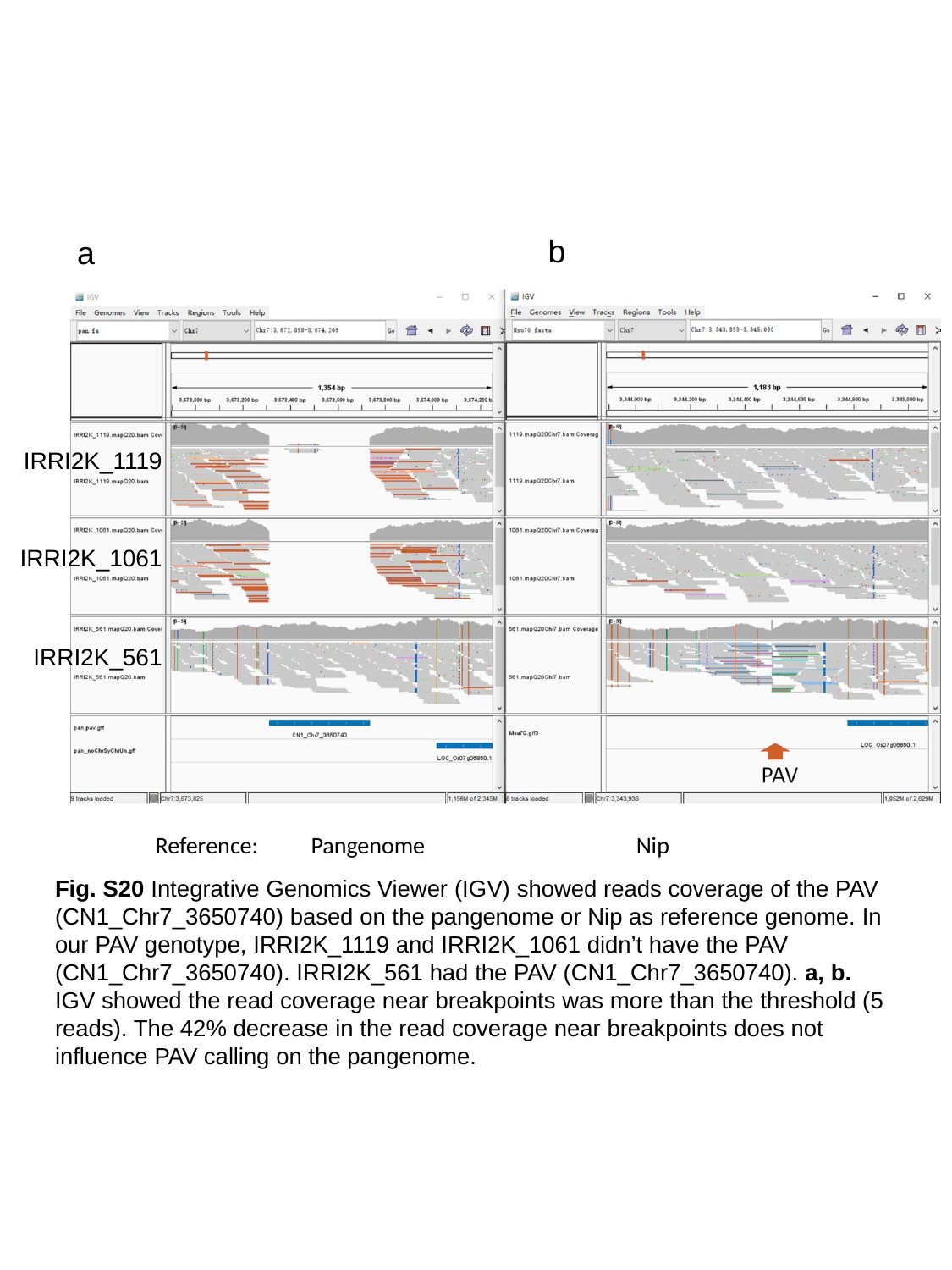

b
a
IRRI2K_1119
IRRI2K_1061
IRRI2K_561
Reference: Pangenome Nip
Fig. S20 Integrative Genomics Viewer (IGV) showed reads coverage of the PAV (CN1_Chr7_3650740) based on the pangenome or Nip as reference genome. In our PAV genotype, IRRI2K_1119 and IRRI2K_1061 didn’t have the PAV (CN1_Chr7_3650740). IRRI2K_561 had the PAV (CN1_Chr7_3650740). a, b. IGV showed the read coverage near breakpoints was more than the threshold (5 reads). The 42% decrease in the read coverage near breakpoints does not influence PAV calling on the pangenome.

## Slide 25
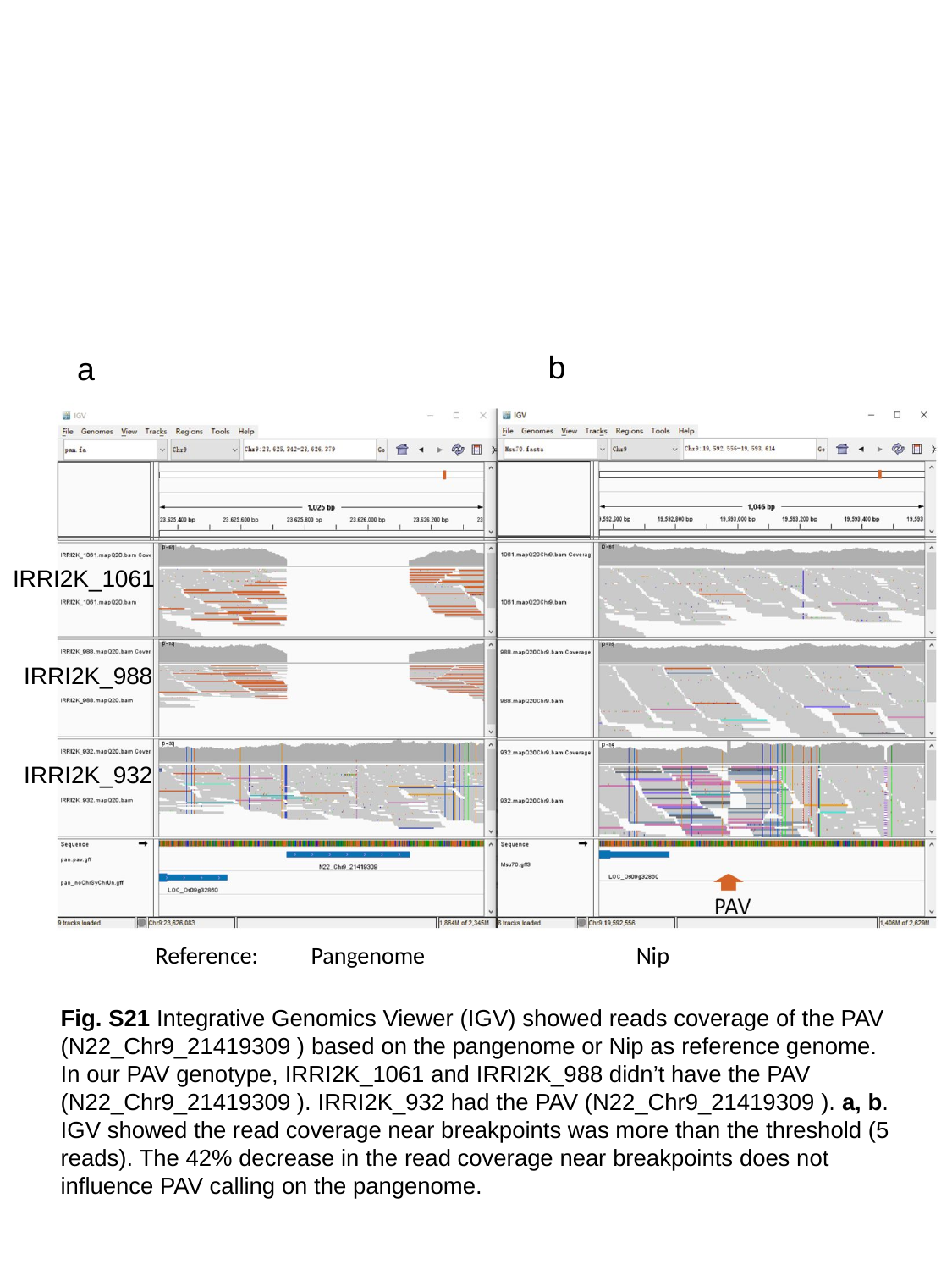

b
a
IRRI2K_1061
IRRI2K_988
IRRI2K_932
Reference: Pangenome Nip
Fig. S21 Integrative Genomics Viewer (IGV) showed reads coverage of the PAV (N22_Chr9_21419309 ) based on the pangenome or Nip as reference genome. In our PAV genotype, IRRI2K_1061 and IRRI2K_988 didn’t have the PAV (N22_Chr9_21419309 ). IRRI2K_932 had the PAV (N22_Chr9_21419309 ). a, b. IGV showed the read coverage near breakpoints was more than the threshold (5 reads). The 42% decrease in the read coverage near breakpoints does not influence PAV calling on the pangenome.

## Slide 26
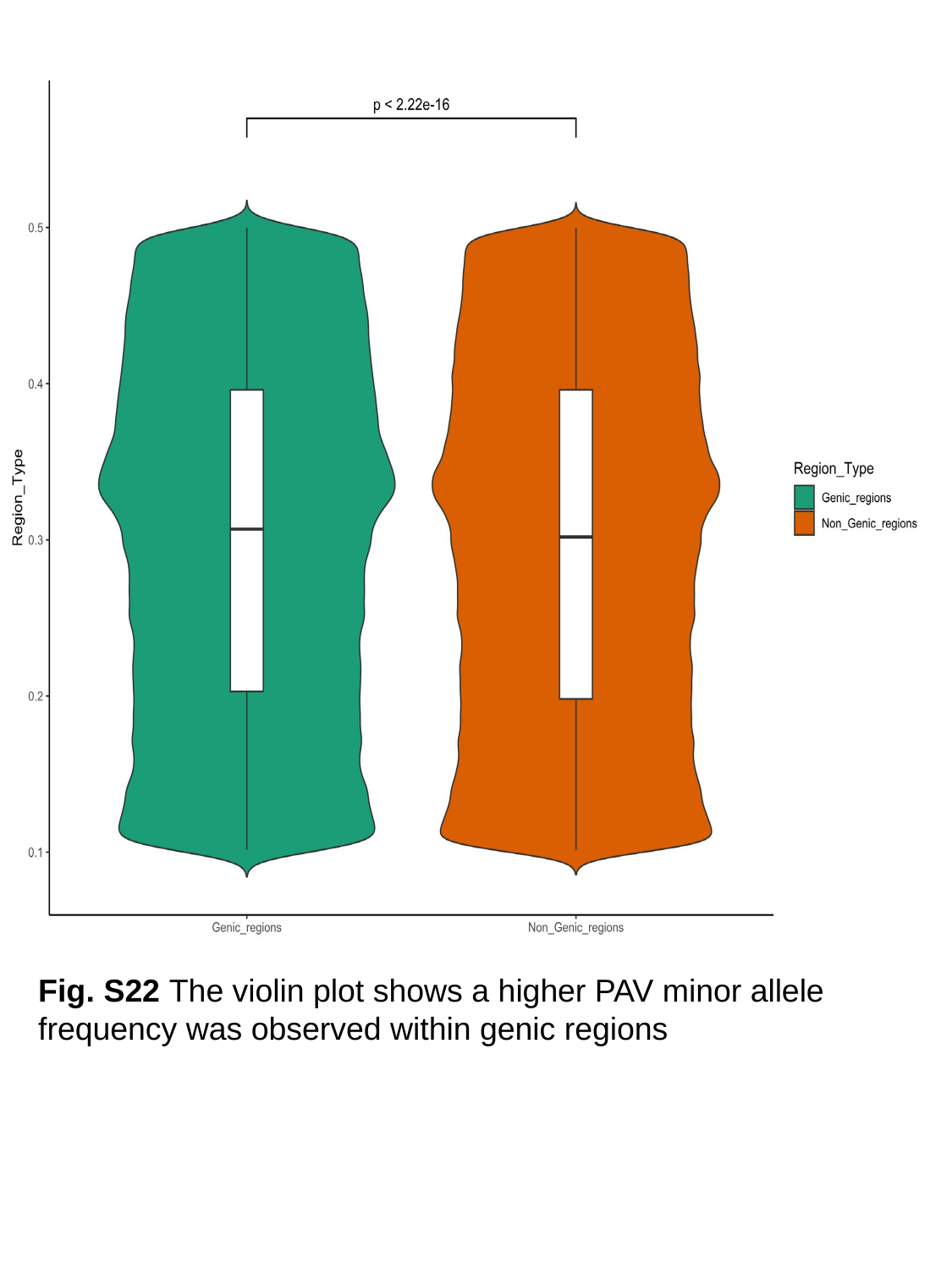

Fig. S22 The violin plot shows a higher PAV minor allele frequency was observed within genic regions

## Slide 27
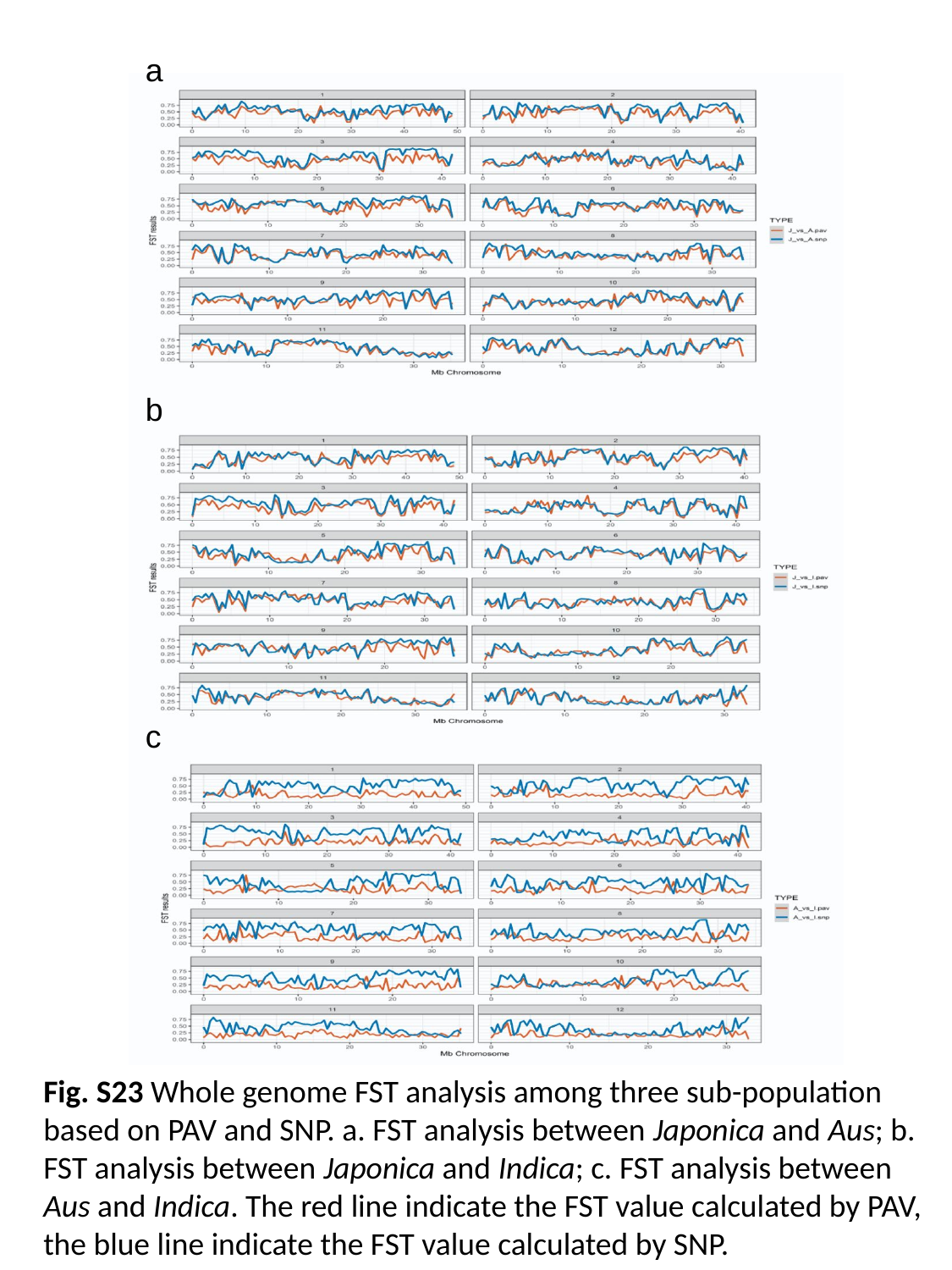

a
b
c
Fig. S23 Whole genome FST analysis among three sub-population based on PAV and SNP. a. FST analysis between Japonica and Aus; b. FST analysis between Japonica and Indica; c. FST analysis between Aus and Indica. The red line indicate the FST value calculated by PAV, the blue line indicate the FST value calculated by SNP.

## Slide 28
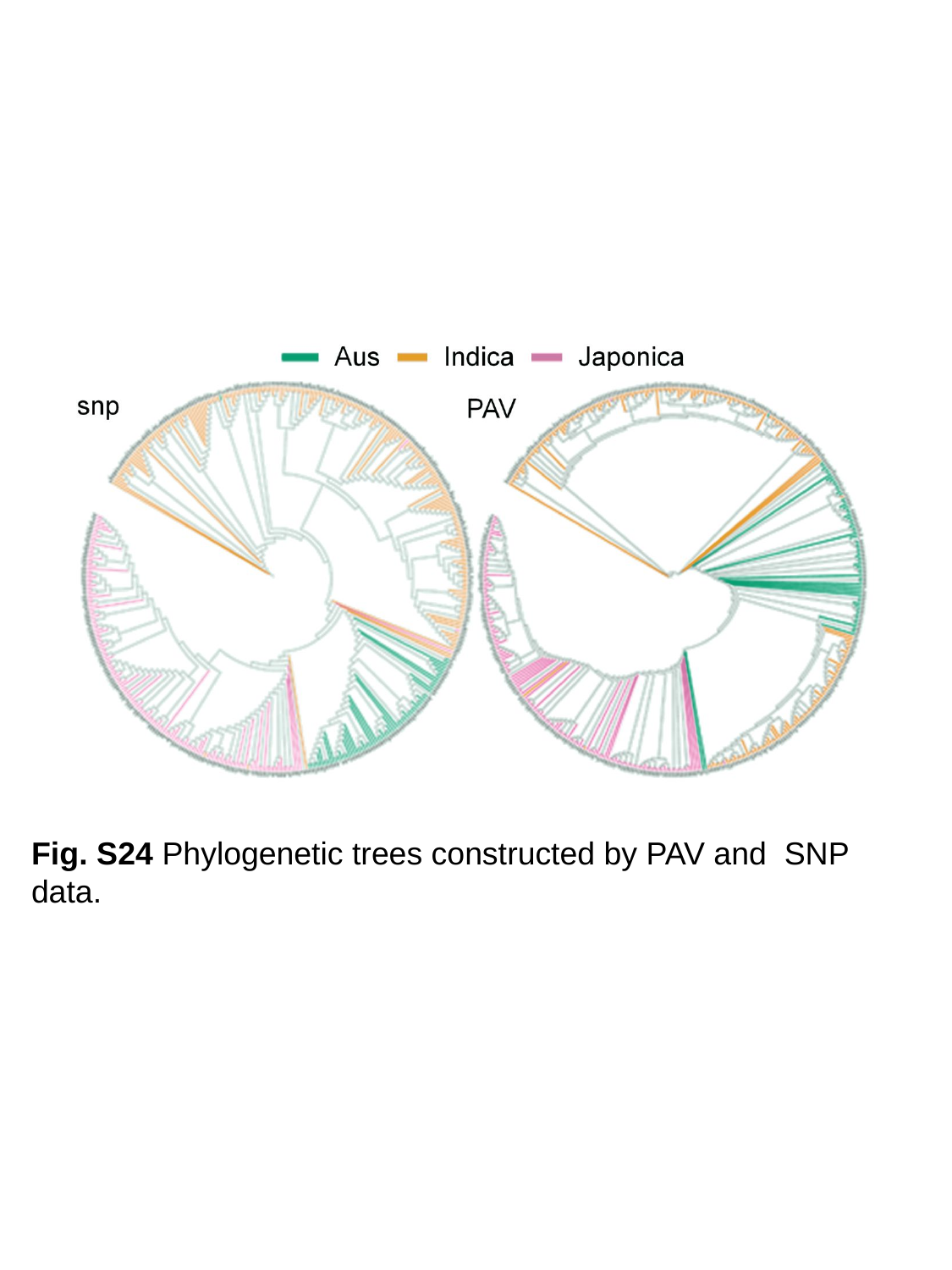

Fig. S24 Phylogenetic trees constructed by PAV and SNP data.

## Slide 29
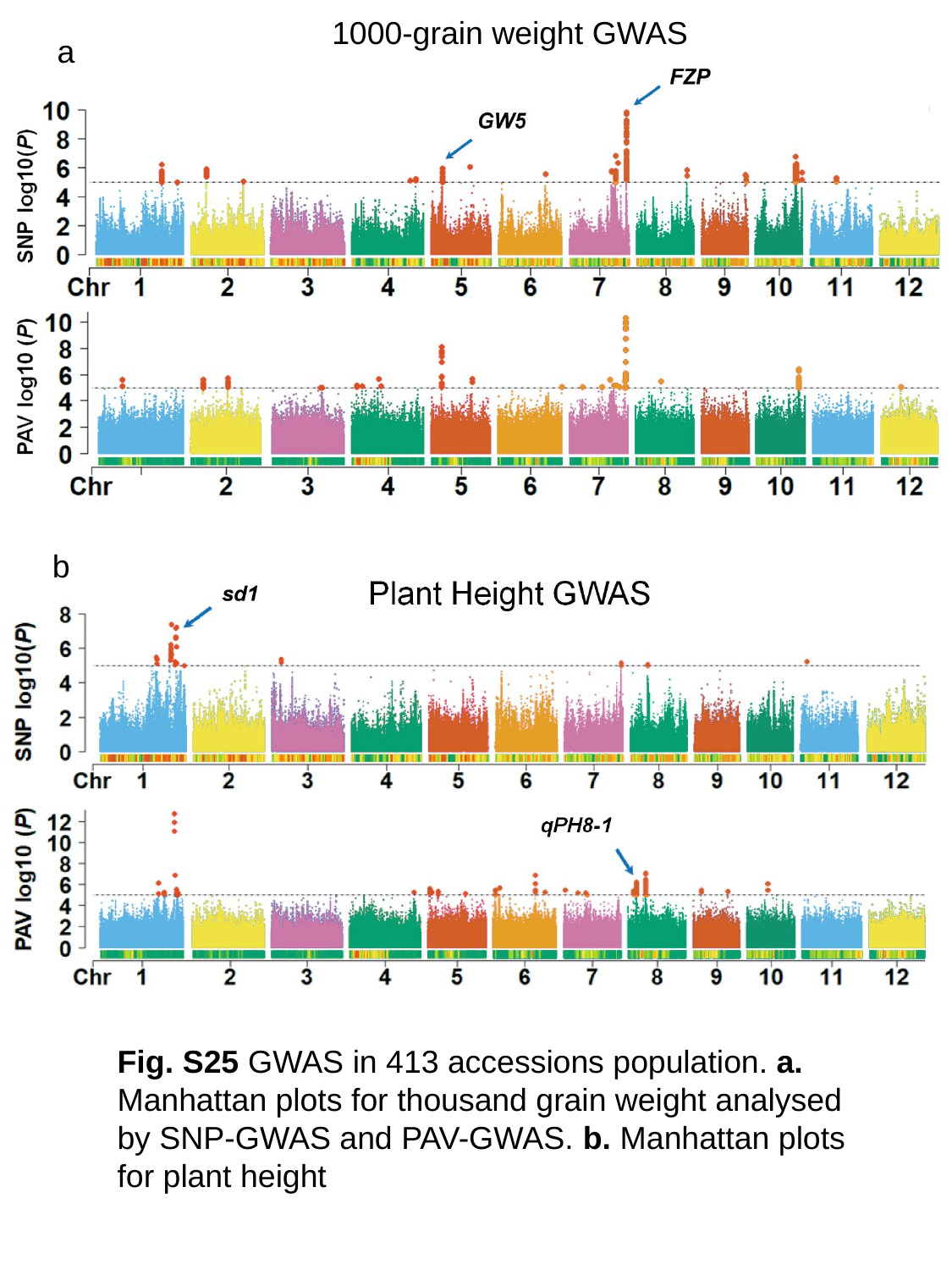

1000-grain weight GWAS
a
b
Fig. S25 GWAS in 413 accessions population. a. Manhattan plots for thousand grain weight analysed by SNP-GWAS and PAV-GWAS. b. Manhattan plots for plant height

## Slide 30
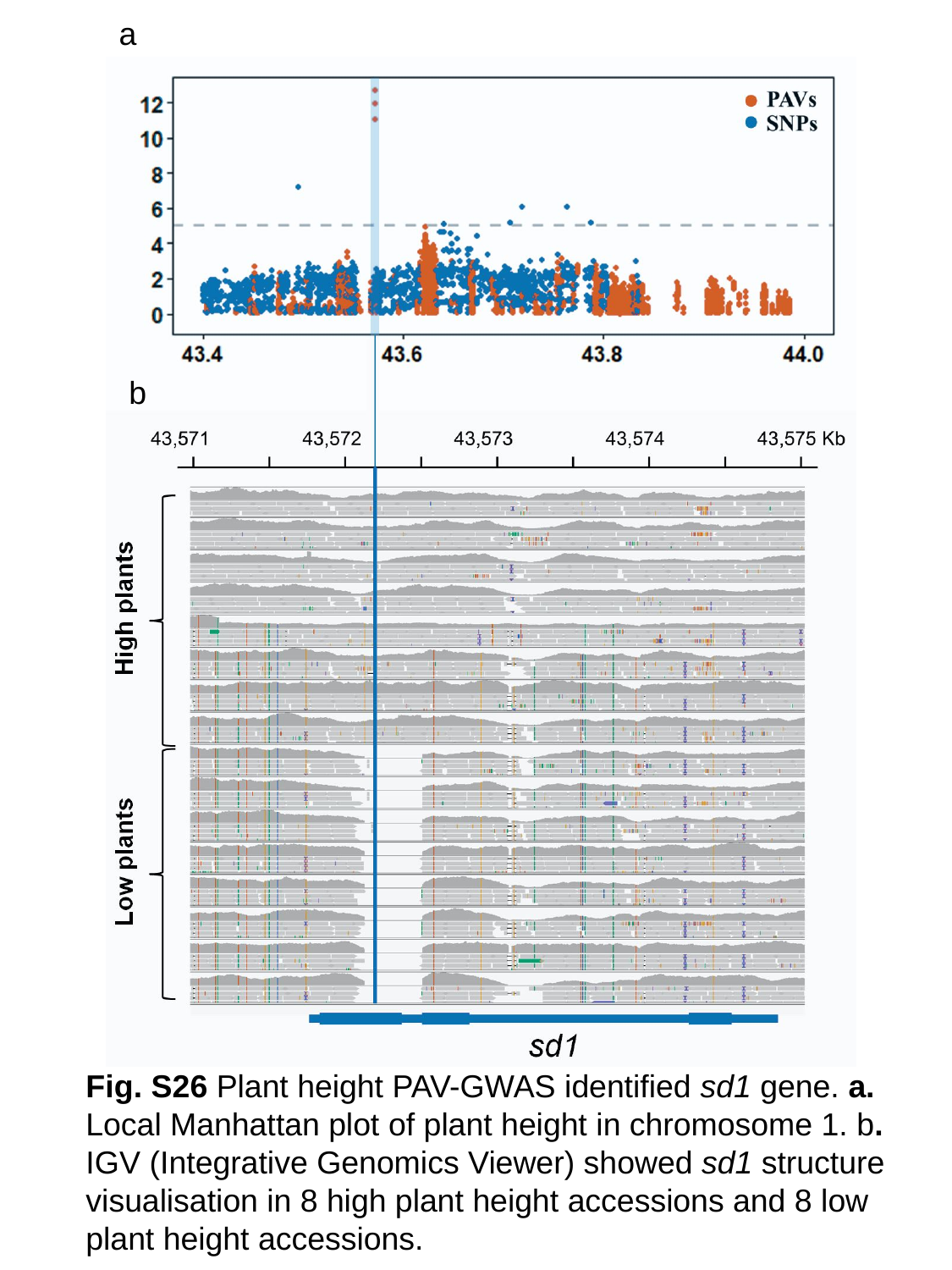

a
b
Fig. S26 Plant height PAV-GWAS identified sd1 gene. a. Local Manhattan plot of plant height in chromosome 1. b. IGV (Integrative Genomics Viewer) showed sd1 structure visualisation in 8 high plant height accessions and 8 low plant height accessions.

## Slide 31
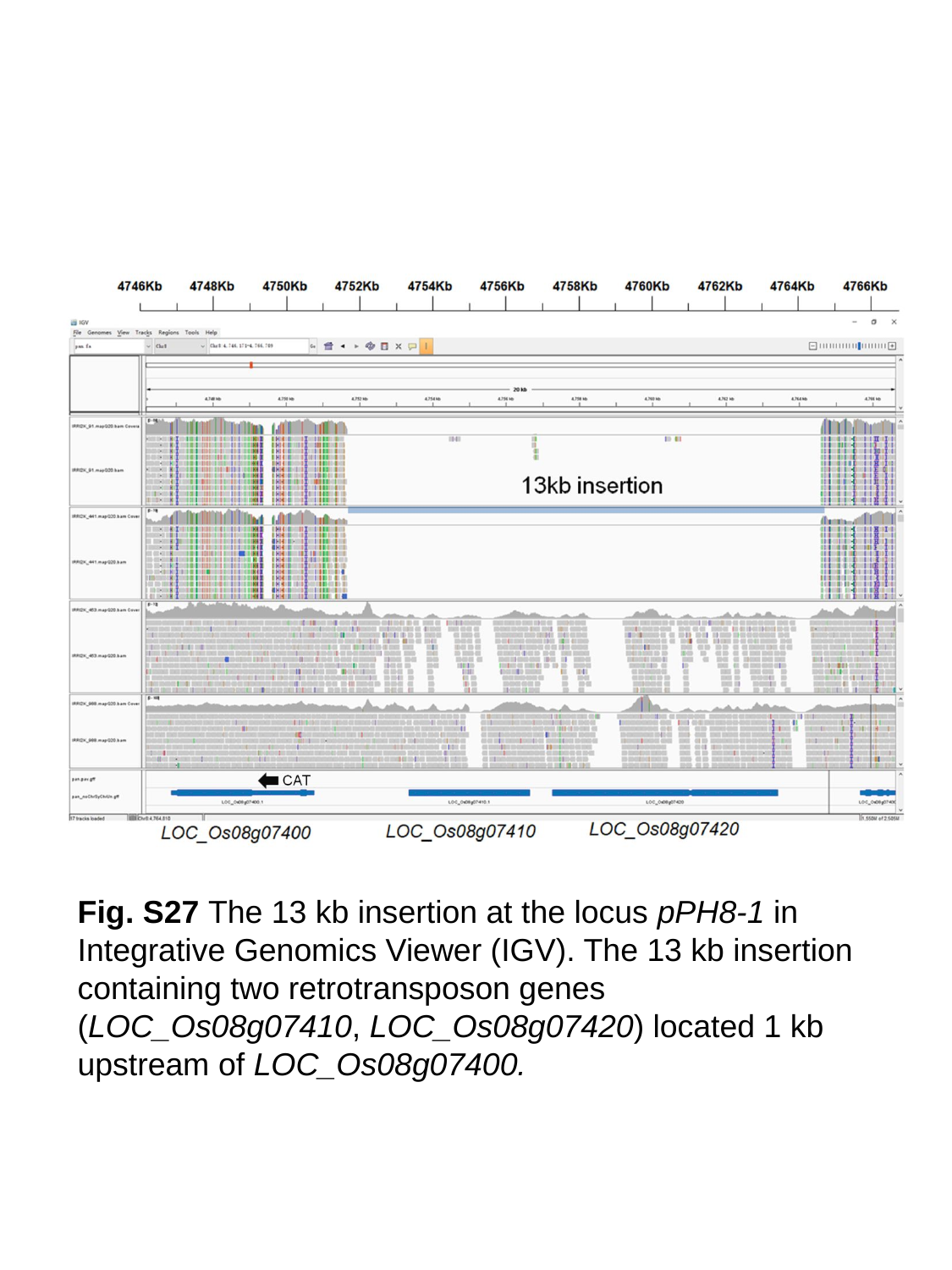

Fig. S27 The 13 kb insertion at the locus pPH8-1 in Integrative Genomics Viewer (IGV). The 13 kb insertion containing two retrotransposon genes (LOC_Os08g07410, LOC_Os08g07420) located 1 kb upstream of LOC_Os08g07400.

## Slide 32
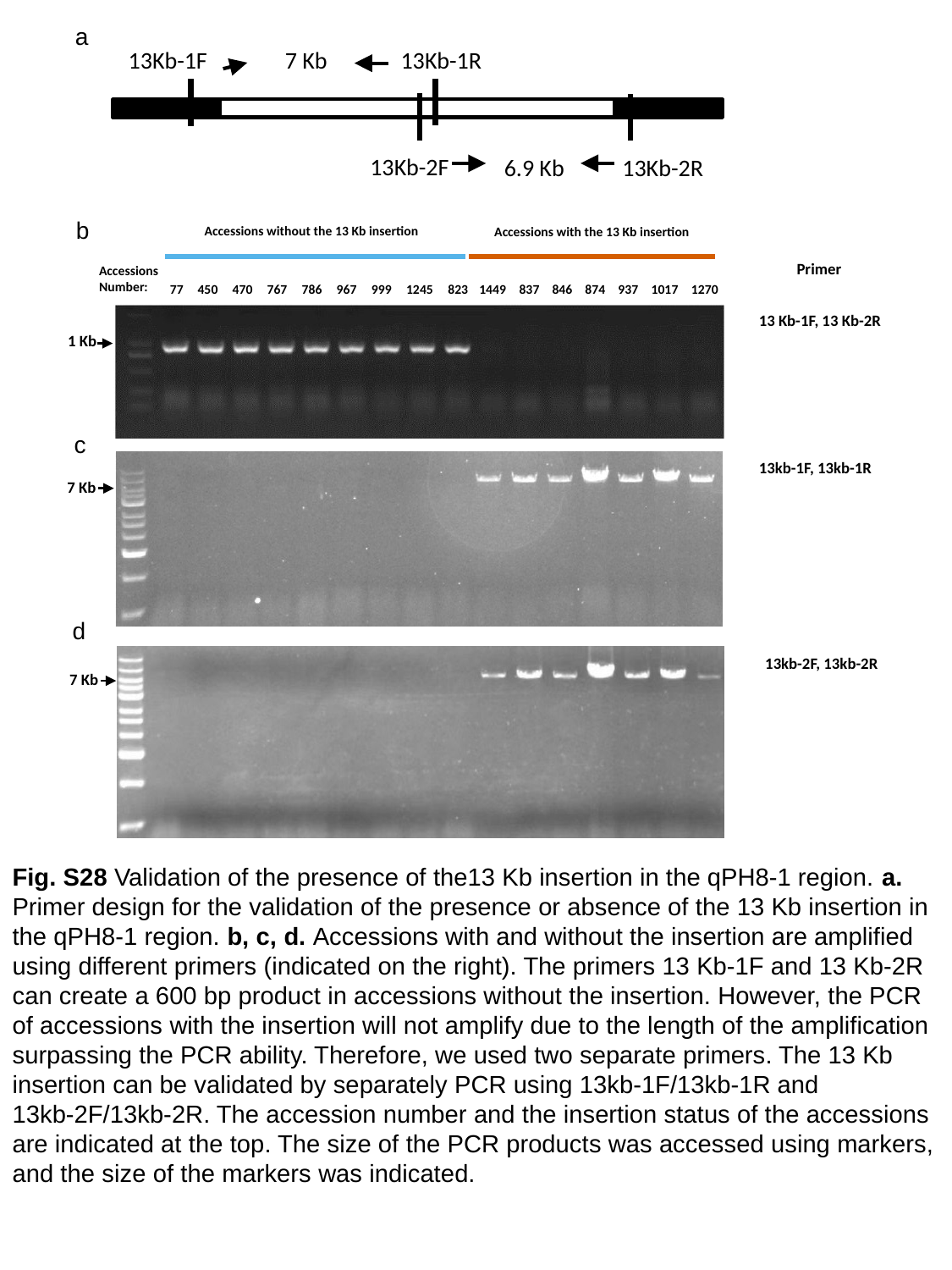

a
7 Kb
13Kb-1F
13Kb-1R
13Kb-2F
6.9 Kb
13Kb-2R
b
Accessions without the 13 Kb insertion
Accessions with the 13 Kb insertion
Primer
Accessions Number:
77
450
470
767
786
967
999
1245
823
1449
837
846
874
937
1017
1270
13 Kb-1F, 13 Kb-2R
1 Kb
c
13kb-1F, 13kb-1R
7 Kb
d
13kb-2F, 13kb-2R
7 Kb
Fig. S28 Validation of the presence of the13 Kb insertion in the qPH8-1 region. a. Primer design for the validation of the presence or absence of the 13 Kb insertion in the qPH8-1 region. b, c, d. Accessions with and without the insertion are amplified using different primers (indicated on the right). The primers 13 Kb-1F and 13 Kb-2R can create a 600 bp product in accessions without the insertion. However, the PCR of accessions with the insertion will not amplify due to the length of the amplification surpassing the PCR ability. Therefore, we used two separate primers. The 13 Kb insertion can be validated by separately PCR using 13kb-1F/13kb-1R and 13kb-2F/13kb-2R. The accession number and the insertion status of the accessions are indicated at the top. The size of the PCR products was accessed using markers, and the size of the markers was indicated.
