# Supplementary material for: A pangenome analysis pipeline provides insights into functional gene identification in rice
Source: Genome Biol. 2023 Jan 26;24:19. doi: 10.1186/s13059-023-02861-9 (PMC9878884; doi:10.1186/s13059-023-02861-9)
Supplement: Supplementary file 3 — Additional file 3: Supplemental notes. [file 13059_2023_2861_MOESM3_ESM.docx]

**A pangenome analysis pipeline provides insights into functional gene identification in rice**

**Supplementary Notes**

Jian Wang^1†^, Wu Yang^1†^, Shaohong Zhang^1^, Haifei Hu^1, 2*^, Yuxuan Yuan^3^, Jingfang Dong^1^, Luo Chen^1^, Yamei Ma^1^, Tifeng Yang^1^, Lian Zhou^1^, Jiansong Chen^1^, Bin Liu^1^, Chengdao Li^2*^, David Edwards^4*^, Junliang Zhao^1*^

^1^ Rice Research Institute & Guangdong Key Laboratory of New Technology in Rice Breeding & Guangdong Rice Engineering Laboratory Guangdong Academy of Agricultural Sciences, Guangzhou 510640, China

^2^ Western Crop Genetics Alliance, Murdoch University, Western Australia, 6150

^3^ School of Life Sciences and State Key Laboratory of Agrobiotechnology, The Chinese University of Hong Kong, Hong Kong SAR, China

^4^ School of Biological Sciences and Centre for Applied Bioinformatics, The University of Western Australia, Perth, WA, Australia

^†^Jian Wang and Wu Yang contributed equally to this work.

*Correspondence: huhaifei@gdaas.cn; c.li@murdoch.edu.au; dave.edwards@uwa.edu.au; zhao_junliang@gdaas.cn

**Genome assemblies of IRRI2K_86 and IRRI2K_91**

The High-molecular-weight genomic DNA of IRRI2K_86 and IRRI2K_91 were sent to Beijing Beirui Hekang Biotechnology (BerryGenomics, China) for next-generation sequencing using the llumina NovaSeq6000 platform and Pacbio sequencing using Pacbio SeqII platform. The Pacbio raw data was implemented to De novo assemble with the software MECAT [1]. A polishing of the assembly was performed with the 100× Illumina reads using NextPloish [2]. Using the Nipponbare genome as a reference, we aligned the assembled contigs using Minimap2 [3]. RaGOO [4] was then used to order the assembly contigs. Space between contigs was artificially filled in with 100 “N” blocks.

**Evaluation of potential translocations and inversions**

To demonstrate the accuracy of translocation identification, we further validated the translocation genotyping results by aligning the Pacbio long read sequencing reads of two lines (IRRI2K_86 and IRRI2K_91) of the 413 accessions to the pangenome using minimap2 v2.2.4 [3]. For example, we found the sequence (Chr11:21,534,691-21,541,116) on pangenome has 96.267% identity as the sequence (Chr5:8,074,149-8,080,588) and these two sequences were defined as a translocation (Additional file2: Table S5). In our translocation genotyping results, the sequence of Chr11:21,534,691-21,541,116 is absent and the Chr5:8,074,149-8,080,588 sequence is present in the IRRI2K_86. In contrast, the sequence of Chr11:21,534,691-21,541,116 is present and the Chr5:8,074,149-8,080,588 sequence is absent in the IRRI2K_91.

The long read alignment result (Additional file1: Fig S6) shows IRRI2K_86 has the reads with a 6,425 bp deletion on Chr11:21,534,691-2,1541,116 and the reads spanning Chr5:8,074,149-8,080,588. IRRI2K_91 had the reads spanning Chr11:21,534,691-21,541,116 and the reads with 6,441 bp deletion on Chr5:8,074,149-8,080,588. We further validated the translocations genotyping results by comparing the genomes of accession IRRI2K_86 and IRRI2K_91 with the pangenome using Mummer v4.0.0 [5]. The result (Additional file1: Fig S9) shows IRRI2K_86 has a 6,425 deletion on Chr11:19,375,935 and IRRI2K_91 has a 6,441 bp deletion on Chr5: 7,144,687. This result demonstrates the accuracy of translocation detection and genotyping in our pipeline.

For inversions, we further validate the inversion genotyping results. For example, we found an inversion on Chr5:13,924,048-13,925,592 (Additional file2: Table S8). In our inversion genotyping results, IRRI2K_91 has the inversion on regions of Chr5:13,924,048-13,925,592 compared to our pangenome, and IRRI2K_86 does not contain the inversion, which was synthetic with our pangenome on regions of Chr5:13,924,048-13,925,592. The Pacbio long read alignment result (Additional file1: Fig S12) shows IRRI2K_86 has long reads spanning 39 bp region with conjunction point (Chr5:13,924,048 conjunction point and Chr5: 13,925,592 conjunction point) in the center. But IRRI2K_91 does not have the long reads spanning 39 bp region with conjunction point (locations: Chr5:13,924,048 and Chr5: 13,925,592) in the center. We further validated the inversion genotyping results by comparing the genome assemblies of accession IRRI2K_86 and IRRI2K_91 with the pangenome using Mummer v4.0.0 [5]. The result shows (Additional file1: Fig S13) the sequence Chr5: 12,329,090-12,407,319 on IRRI2K_86 is consistent with the sequence Chr5: 13902408- 13,980,631 on pangenome. Therefore, this indicates the sequence Chr5: 12,391,459-12,393,001 on IRRI2K_91 is an inversion of the sequence Chr5:13,924,048- 13,925,592 on the pangenome.

**Evaluation of mapping of short reads near breakpoints**

Three PAV sequences (Tumba_Chr5_5,526,446, CN1_Chr7_3,650,740 and N22_Chr9_21,419,309) confirmed by PCR amplification were used for the evaluation of mapping of short reads near breakpoints. We mapped the short reads of the selected accessions against the pangenome and Nip reference genome using BWA MEM [6]. The read coverages of every 20 bp near the breakpoints were counted. The positions in the Nip reference genome were converted to the pangenome position coordinates.

The results (Additional file1: Fig S18 and Additional file2: Table S12) discover that if the accession does not contain the insertion sequence introduced by pangenome construction, there would be about a 44% decrease in the read coverage near breakpoints using pangenome compared to the Nipponbare reference. Based on our evaluation, with sufficient sequencing coverage of short-read sequencing data, mapping coverage decreased near breakpoints does not affect PAV calling in our pipeline.

**Reference**

1. Xiao C-L, Chen Y, Xie S-Q, Chen K-N, Wang Y, Han Y, Luo F, Xie Z: **MECAT: fast mapping, error correction, and de novo assembly for single-molecule sequencing reads.** *nature methods* 2017, **14:**1072-1074.

2. Hu J, Fan J, Sun Z, Liu S: **NextPolish: a fast and efficient genome polishing tool for long-read assembly.** *Bioinformatics* 2020.

3. Li H: **Minimap2: pairwise alignment for nucleotide sequences.** *Bioinformatics* 2018, **34:**3094-3100.

4. Alonge M, Soyk S, Ramakrishnan S, Wang X, Goodwin S, Sedlazeck FJ, Lippman ZB, Schatz MC: **RaGOO: fast and accurate reference-guided scaffolding of draft genomes.** *Genome biology* 2019, **20:**1-17.

5. Marçais G, Delcher AL, Phillippy AM, Coston R, Salzberg SL, Zimin A: **MUMmer4: A fast and versatile genome alignment system.** *PLoS computational biology* 2018, **14:**e1005944.

6. Li H: **Aligning sequence reads, clone sequences and assembly contigs with BWA-MEM.** *arXiv preprint arXiv:13033997* 2013.
