# Supplementary material for: A pangenome analysis pipeline provides insights into functional gene identification in rice
Source: Genome Biol. 2023 Jan 26;24:19. doi: 10.1186/s13059-023-02861-9 (PMC9878884; doi:10.1186/s13059-023-02861-9)
Supplement: Supplementary file 4 — Additional file 4. Uncropped images for the blots in Figure S3 and Figure S28. [file 13059_2023_2861_MOESM4_ESM.pptx]

## Slide 1
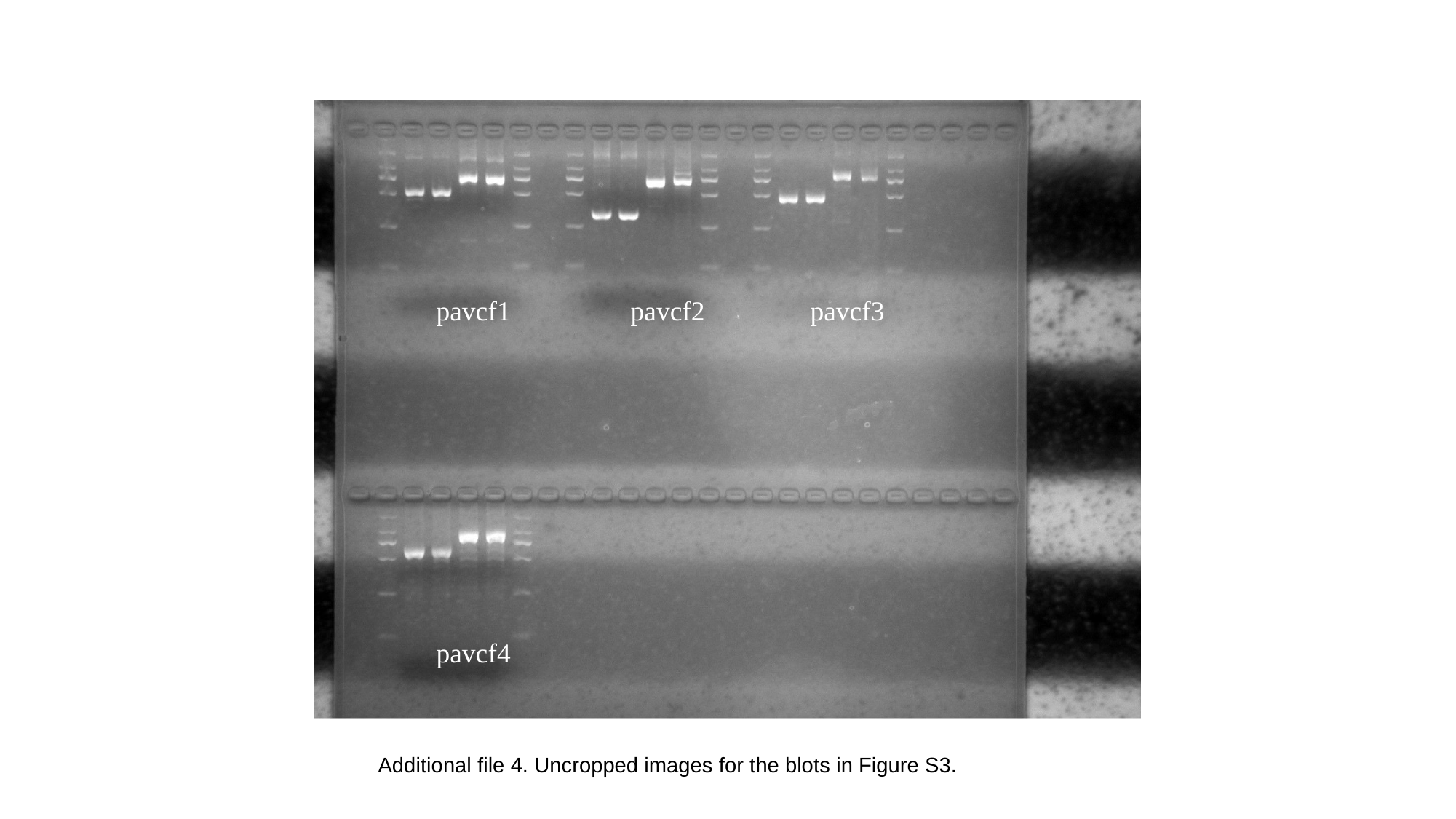

pavcf1
pavcf2
pavcf3
pavcf4
Additional file 4. Uncropped images for the blots in Figure S3.

## Slide 2
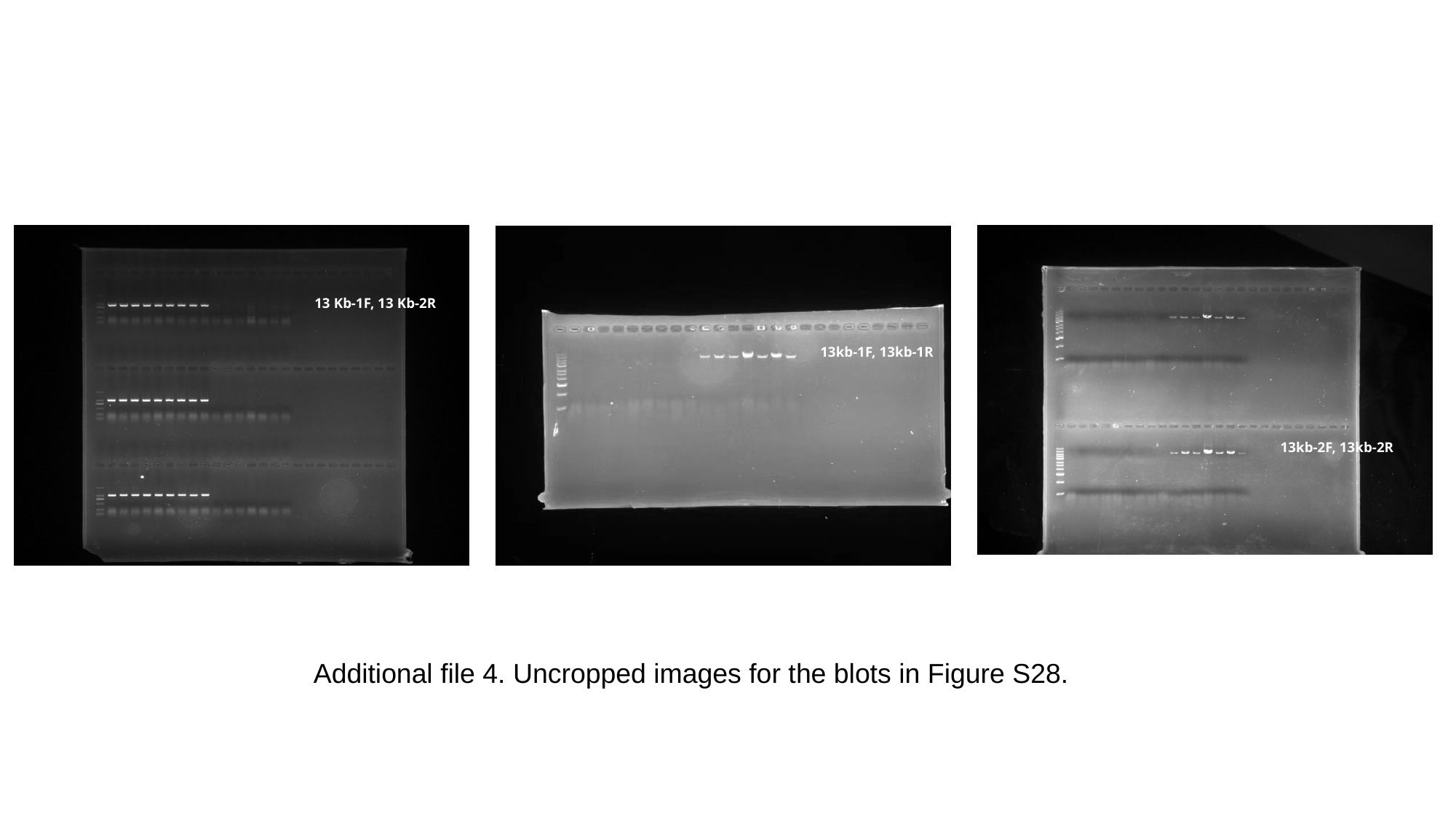

13 Kb-1F, 13 Kb-2R
13kb-1F, 13kb-1R
13kb-2F, 13kb-2R
Additional file 4. Uncropped images for the blots in Figure S28.
