# Supplementary material for: A pangenome analysis pipeline provides insights into functional gene identification in rice
Source: Genome Biol. 2023 Jan 26;24:19. doi: 10.1186/s13059-023-02861-9 (PMC9878884; doi:10.1186/s13059-023-02861-9)
Supplement: Supplementary file 5 — Additional file 5. Review history. [file 13059_2023_2861_MOESM5_ESM.docx]

Review History

**First round of review**

**Reviewer 1**

**Were you able to assess all statistics in the manuscript, including the appropriateness of statistical tests used?**

No, I do not feel adequately qualified to assess the statistics.

**Comments to author:**

In this manuscript, Wang and colleagues introduce a new analysis pipeline to construct pangenomes. In contrast to current direction in the field, the authors do not construct a graph-base pangenome but a coordinate-linked linear pangenome to allow implementation of standard computational tools for downstream genomics analyses. The authors use their approach to construct a pangenome for rice and identify previously known and unknown loci that are associated with variation in grain size and plant height. The manuscript is written well, however, most figure legends are very sparse and lack essential information. Overall, the linear pangenome approach is presented as a straight-forward approach for identifying hidden genetic variation with phenotypic consequences and could be useful for studies in plants and other species.

Major comments:

L99: Can the authors explain how mapping of short reads will be influenced by the many breakpoints of the segments that are introduced during the pangenome construction (see Fig. 1A)?

L100: The authors explain that they call PAVs between genomes. However, it remains unclear how linear pangenomes display more complicated SVs such as translocations and inversions. Also, how does the quality of the input assemblies affect the construction of linear pangenomes.

L228: The authors describe Hap1 in the text as haplotype with both a 1'212 bp and a 950 bp deletion. However, Hap1 is displayed in Fig. 5C as haplotype with 370 bp deletion. Please explain this discrepancy.

L244: The authors identify a 13 kb insertion at the locus pPH8-1, which is located 1 kb upstream of LOC_Os08g07400. However, in line 252, the authors write that LOC_Os08g07400 is located 2 kb downstream of the 13 kb insertion. Please explain this discrepancy. The authors should present a model of the haplotype with exact coordinates and sizes for the insertion(s) and associated gene(s).

L400: The authors explain that missing linkage of the retrotransposon insertion with SNPs in the flanking region at pPH8-1 may be caused by recent insertion events. This explanation would suggest that the transposon insertion occurred multiple times in many independent haplotypes. Can the authors comment?

Fig. 6C: The authors compare expression levels of LOC_Os08g07400 between lines with and without the 13-kb retrotransposon element. However, statistics are missing to compare the two groups and it remains unclear whether the presence of the 13 kb insertion is associated with significant differences in gene expression. Also, the legend lacks information about error bars (s.d. or s.e.), sample sizes, and the explanation for panel C "presence of 13-kb" should be written more precise. The gene expression and plant height data should be better displayed in box plots.

L256 and Fig. 7: The authors find very low linage between the 13 kb insertion at pPH8-1 and SNPs in the flanking regions. Given this surprising observation, the authors should experimentally confirm the presence of the 13 kb insertion at pPH8-1 by amplifying the insertion sites using PCR and rule out the possibility of an assembly error or translocation event.

Minor:

L72: The authors point out that the limitation of graph-based genomes is that common bioinformatic tools are not suitable for graph-based genomes. However, recent studies (e.g. https://www.nature.com/articles/s41586-022-04808-9) demonstrate the power of graph-based pangenomes and how standard tools (Kallisto etc.) can be readily applied.

Fig. 5C: The red star above Hap1 is not described in the legend.

L230: Please clarify how the 1'212 bp deletion upstream of qSW5 can disrupt the coding region of GW5. In Fig. 5C, the 1'212 bp deletion is far away from the coding region of the GW5 gene.

L279: The sentence "Furthermore, which are common in plants." is incomplete.

L281: The authors write that it may be challenging to present the complexity of plant genomes in graph-based pangenomes. They use a citation to support this statement (ref. 30), however, the cited author (Hübner) proposes that "the graph-pangenome system can become a standard platform".

L432: Since the manuscript introduces a new pipeline for pangenome construction and analyses, the authors should make the code available in a public repository (GitHub) to allow reproduction of their results and/or construction of pangenomes in other species.

Fig. A and B: The legend does not explain the difference between (A) and (B) but it appears that (B) is a zoomed-in version of (A). However, in (A) there are two regions at ~5Mbp that surpass the significance threshold of 5 -log(P) while in (B) there is only one significant region. The authors should specify in the legend the difference between (A) and (B) and explain the absence of the second significant region in (B).

**Reviewer 2**

**Were you able to assess all statistics in the manuscript, including the appropriateness of statistical tests used?**

Yes, and I have assessed the statistics in my report.

**Comments to author:**

Summary of paper:

It has been well known that genomic diversity of a species cannot be presented by a single reference genome, which highlights the importance of pan-genome analyses. In this manuscript, the authors developed a pipeline (PSVCP) for a linear pan-genome construction and PAV genotype calling. They tested the pipeline by constructing a high-quality rice pan-genome using reference genomes of 12 cultivars and the pan-genome had higher contiguity and BUSCOs than the previous 3K rice pan-genome. Capturing coordinate information of PAVs in pan-genome is a novel approach and useful for downstream analysis, including PAV-based GWAS. They also compared PAV-based and SNP-based GWAS for thousand grain weight and plant height using 413 international rice accessions and the new pan-genome, which shows the strength of PAV-based GWAS over SNP-based GWAS. The manuscript is valuable for future pan-genome studies and the PSVCP pipeline is likely to be widely used for pan-genomics.

Major comments:

1. In the pan-genome construction part of PSVCP, the pipeline seems straightforward by adding new additional sequences to the reference genome from each previous step. It is unclear though how other SVs like translocations and inversions, which might have variable flanking sequences, are handled in the pipeline? The manuscript would be improved by addressing this explicitly. One suggestion would be to test in silico the effects of inversions, translocations of different sizes, regions. Are these seen as novel sequence in the resulting linear pan-genome, and how do they affect downstream mapping and variant calling?

2. It would be great to have a list and details of the 413 rice accessions as a supplementary table and also to release the PAV matrix.

3. Figure legends and methods sections require more details in general.

Minor comments:

Any specific reasons to include only the 413 accessions re-sequenced lines rather than the >3000 that are available? As it currently stands the paper is largely about methods but were a larger selection of genotypes used it could be elevated to provide a major resource for the rice genomics community. It seems relatively straightforward for the authors to run the pipeline on a complete dataset of short read data across rice and provide the resulting PAV matrix as a supplemental dataset, even without changing the core of the paper focusing on the 413 lines they resequenced.

There seems to be a missed opportunity to test selection on PAV. The dataset the authors generated seems powerful to address various hypotheses. The authors could, for example, ask whether genic PAV have lower minor allele freq (purifying selection) than intergenic PAV.

Pg 2, Line 60, "efficiently" should be "efficient".

Pg 3, Line 101, "level based" could be just "level".

Pg 3, Line 101, remove "'" at the end of accessions'

Pg 3. Lines 111-114, It is confusing where 35.7 % came from. It would be helpful to make it clear if 35.7% is the percentage out of 43.1% or out of the total 100%. In addition, 6,797 sequences were fully nested in 5,925 Nipponbare genes? That means ~27.6% sequences out of 100% (24,585 sequences) were completely resided in the genes?

Pg 5, Line 231, "accord" should be "accordance".

Pg 7, Line 279, "Furthermore, which are common in plants" should be re-written.

Pg 8, Line 350, "The phylogeny variations" could be rephrased as "The topological variation between SNP- and PAV-based phylogenetic analyses".

Pg 10, Line 434, Is MSU same as NIP because I do not see MSU in Table S1, but NIP instead.

Pg 10, Line 437, I found that the acronym "NIP" was first mentioned here, so it should be clarified what this means here. "Nipponbare (NIP)"

In Figure 4, The color of lines should be labeled in Fst Analysis.

In the legend of Figure 4, "datga" needs to be changed to "data"?

The authors should cite: https://www.nature.com/articles/s41422-022-00685-z.

**Authors Response**

**Point-by-point responses to the reviewers’ comments:**

Reviewer #1:

In this manuscript, Wang and colleagues introduce a new analysis pipeline to construct pangenomes. In contrast to current direction in the field, the authors do not construct a graph-base pangenome but a coordinate-linked linear pangenome to allow implementation of standard computational tools for downstream genomics analyses. The authors use their approach to construct a pangenome for rice and identify previously known and unknown loci that are associated with variation in grain size and plant height. The manuscript is written well, however, most figure legends are very sparse and lack essential information. Overall, the linear pangenome approach is presented as a straight-forward approach for identifying hidden genetic variation with phenotypic consequences and could be useful for studies in plants and other species.

Major comments:

1. L99: Can the authors explain how mapping of short reads will be influenced by the many breakpoints of the segments that are introduced during the pangenome construction (see Fig. 1A)?

*Response: Thanks for this comment. We have investigated short read mapping near the PAV breakpoints. In our pipeline, a sequence was determined as present if its read coverage is larger than 5x in a 20bp window. We found an average of 18.5x (44%) decrease in the read coverage near 20bp of the break point for the accessions without the PAV and an average of 15.8x(46%) increase in the read coverage near 20bp of the break point for the accessions containing the PAV. With an average of 42x coverage in our short-read sequencing data, these changes in the read coverage near the breakpoint do not affect our PAV calling results. We have provided three examples in Figure S18-S21 and Tables 12-14, and described the results in line 164-168 to demonstrate that the read coverage changes near breakpoints do not influence PAV calling results. Based on our evaluation, with sufficient sequencing coverage of short-read sequencing data, mapping coverage decrease near breakpoints does not affect PAV calling in our pipeline. Furthermore, we have provided a recommended sequencing coverage of more than 10x for users on our pipeline GitHub page.*

2. L100: The authors explain that they call PAVs between genomes. However, it remains unclear how linear pangenomes display more complicated SVs such as translocations and inversions. Also, how does the quality of the input assemblies affect the construction of linear pangenomes.

*Response: Thanks for the reviewer's comments. Identification and display of other large SVs, such as translocations and inversions, are still challenging in pangenomics studies, even for the advanced graph-based pangenome. Therefore, the current main stream linear pangenomes approaches cannot display translocations and inversions. We have included additional content in our manuscript to discuss this limitation (see lines 361-366)*

*Although it is hard to display the large SVs directly, we have attempted to improve our pipeline by including additional functions to provide the position information of large SVs (translocations and inversions) and genotype potential translocations and inversions at the population level using short reads. See details below:*

*We aligned novel insertions to the reference genome using BLAST+ and identified novel insertions that can further be classified as translocations. We determined the presence or absence of the translocation by looking at the read mapping around its breakpoints, examining reads spanning a 39 bp region with a conjunction point at the center. Using this method, we identified potential translocations in the pangenome. We further genotyped all translocations based on the short-read mapping results by calculating the coverage of the reads spanning the junctions (39 bp region with conjunction point in the center) of the translocation in the pangenome. We have included a further description of the translocations results in lines 160-164.*

*To demonstrate the accuracy of translocation identification, we validated the translocation genotyping results by aligning the Pacbio long read sequencing reads of two lines (IRRI2K_86 and IRRI2K_91) from the 413 accessions to the pangenome. We found the sequence (Chr11:21,534,691-21,541,116) on the pangenome has 96.267% identity to the sequence (Chr5:8,074,149-8,080,588) and these two sequences were defined as a translocation. In our translocation genotyping results, the sequence of Chr11:21534691-21541116 is absent and the Chr5:8074149-8080588 sequence is present in the IRRI2K_86. In contrast, the sequence of Chr11:21534691-21541116 is present and the Chr5:8074149-8080588 sequence is absent in the IRRI2K_91.*

*The long read alignment result showed IRRI2K_86 had the reads with a 6425 bp deletion on Chr11:21534691-21541116 and the reads spanning Chr5:8074149-8080588. IRRI2K_91 had the reads spanning Chr11:21534691-21541116 and the reads with 6441 bp deletion on Chr5:8074149-8080588. We further validated the translocations genotyping results by comparing the genomes of accession IRRI2K_86 and IRRI2K_91 with the pangenome using Mummer. The result showed IRRI2K_86 had a 6425 deletion on Chr11:19375935 and IRRI2K_91 had a 6441 bp deletion on Chr5: 7144687. The result demonstrates the accuracy of translocation detection and genotyping in our pipeline, which is summarized in Figures S6-S11 and Tables S5-S7.*

*For inversions, we identified the inversions by comparing each genome used for pangenome construction with the pangenome using Assemblitics. We further genotyped each potential inversion in the pangenome from 413 accessions by examining the mapping coverage of the 39 bp region with breakpoints in the centre. We further validate the inversion genotyping results by comparing the genomes of accession IRRI2K_86 and IRRI2K_91 with the pangenome using mummer. We found an inversion on Chr5:13924048-13925592. In our inversion genotyping results, IRRI2K_91 had the inversion in regions of Chr5:13924048-13925592 compared to our pangenome and IRRI2K_86 did not contain the inversion, which was syntenic with our pangenome in regions of Chr5:13924048-13925592. The Pacbio long read alignment results showed IRRI2K_86 had long reads spanning 39 bp region with conjunction point (Chr5:13924048 conjunction point and Chr5: 13925592 conjunction point) in the center. But IRRI2K_91 did not have the long reads spanning the 39 bp region with conjunction point (Chr5:13924048 conjunction point and Chr5: 13925592 conjunction point) in the center. We further validated the inversion genotyping results by comparing the genomes of accession IRRI2K_86 and IRRI2K_91 with the pangenome using mummer. The results showed the sequence Chr5: 12329090-12407319 on IRRI2K_86 is consistent with the sequence Chr5: 13902408- 13980631 on the pangenome. Therefore, this indicates the sequence Chr5: 12391459-12393001 on IRRI2K_91 is an inversion of the sequence Chr5:13924048- 13925592 on the pangenome. The result is summarized in Figures S12-S17 and Table S8-S11.*

*Since identifying and genotyping translocations and inversions are still challenging using short-read sequencing, we carefully described the identified SVs as potential inversions and translocations and included additional sentences to inform the limitations of short-reading sequencing for identifying translocation and inversion in lines 361-366 and our GitHub page.*

*See changes: “PSVCP is robust in placing novel PAV sequences into the linear pangenome, it may be limited to display more complex SVs such as translocations and inversions, which is a challenge in current pangenomics studies, even for the advanced graph-based pangenome. In addition, due to its read length, short-read sequencing data may have lower sensitivity for SVs detection compared with long-read sequencing data.”*

*As for the second question, the quality of the input assemblies is important for the linear pangenome construction using our pipeline since the novel PAV sequence identification relies on whole genome comparisons. The genomes we used for pangenome construction were assembled to high-quality and the BUSCO and N50 of these genomes are now provided in Table S1. High-quality genome assemblies enable high accuracy in genome comparison and SV identification. Therefore, we suggest that users should use high-quality genome assemblies for pangenome construction by our pipeline (described on our Github page).*

3. L228: The authors describe Hap1 in the text as haplotype with both a 1'212 bp and a 950 bp deletion. However, Hap1 is displayed in Fig. 5C as haplotype with 370 bp deletion. Please explain this discrepancy.

*Response: We are sorry for the unclear description of our results in the manuscript. To clarify this description, we have revised the manuscript accordingly in lines 263-269 as following: “We further analysed the PAV genotypes, and three haplotypes (Hap1-3) according to the pangenome were identified in our GWAS panel. The accessions with Hap1 (containing the 370bp deletion and without the 1,212 bp and 950 bp deletions) showed significantly lower grain weight than accessions with the other two haplotypes (Hap2, Hap3) with p-values (two-tailed student’s t-test) of 7.47X10-5 and 6.02X10-9 respectively (Fig. 5c)."*

4. L244: The authors identify a 13 kb insertion at the locus pPH8-1, which is located 1 kb upstream of LOC_Os08g07400. However, in line 252, the authors write that LOC_Os08g07400 is located 2 kb downstream of the 13 kb insertion. Please explain this discrepancy. The authors should present a model of the haplotype with exact coordinates and sizes for the insertion(s) and associated gene(s).

*Response: We are sorry for the discrepancy in our description. The identified 13 kb insertion is located 1 kb upstream of LOC_Os08g07400, while the transposon is located 2 kb upstream of the gene. We have corrected and revised this sentence in lines 290-292 as following: “Expression analysis showed that the presence and absence of this 13 Kb sequence significantly correlated with the expression level of LOC_Os08g07400, located 1 kb downstream from the 13 Kb sequence”.*

*This discrepancy was because we confused the two different elements in the description, one is the sequence PAV, and the other is a transposon. This was not related to the coordinate of the pangenome. We now include a figure to clarify this. See Fig S27.*

5. L400: The authors explain that missing linkage of the retrotransposon insertion with SNPs in the flanking region at pPH8-1 may be caused by recent insertion events. This explanation would suggest that the transposon insertion occurred multiple times in many independent haplotypes . Can the authors comment?

*Response: Thanks for the reviewer's comment. Our statement is based on the assumption that transposon insertion occurred multiple times in independent haplotypes. This assumption has been discussed by Lewerentz et al. (Lewerentz et al., Transposon activity, local duplications and propagation of structural variants across haplotypes drive the evolution of the Drosophila S2 cell line. 2019 BMC Genomics, 23: 276) and Lai et al. (Lai et al, Gene movement by Helitron transposons contributes to the haplotype variability of maize. PNAS, 2005, 102(25): 9068-9073) that independent transposon insertions can contribute to haplotypes variations. To clarify this, we have rewritten the sentence on lines 454-456 and included two additional citations to support our statement.*

*See changes: ”Recent independent retrotransposon insertion may cause the low LD of SNPs by breaking previous linkage disequilibrium across haplotypes”.*

6. Fig. 6C: The authors compare expression levels of LOC_Os08g07400 between lines with and without the 13-kb retrotransposon element. However, statistics are missing to compare the two groups and it remains unclear whether the presence of the 13 kb insertion is associated with significant differences in gene expression. Also, the legend lacks information about error bars (s.d. or s.e.), sample sizes, and the explanation for panel C "presence of 13-kb" should be written more precise. The gene expression and plant height data should be better displayed in box plots.

*Response: Thanks for the comment. We have remade the box plots in Fig 6c and used the two-tailed Student's t-tests to determine the significant differences to better display the associations between the presence of the 13kb insertions and the expression level of LOC_Os08g07400. In addition, we have provided further information in the Fig 6 legend to describe the missing information (error bars and sample sizes).*

7. L256 and Fig. 7: The authors find very low linage between the 13 kb insertion at pPH8-1 and SNPs in the flanking regions. Given this surprising observation, the authors should experimentally confirm the presence of the 13 kb insertion at pPH8-1 by amplifying the insertion sites using PCR and rule out the possibility of an assembly error or translocation event.

*Response: Thanks for the suggestions. Based on our PAVs phenotyping results, we have randomly selected nine accessions without this insertion and seven accessions with this insertion for PCR validation. We have included these results in Fig S28. The used primer is shown in Table S25.*

*PCR using the primers spanning the whole insertion resulted in a PCR product of 600 bp in accessions without the insertion, while no product was amplified in accessions containing this insertion. Due to the restriction of the ability of PCR to amplify large segments, we separated the insertion into two segments and designed two primer sets to amplify each of these segments respectively. The results (Fig S28 c, d) showed no amplification in accessions without the 13kb insertion, while both segments can be amplified in accessions with the insertion.*

*The results validated our study's PAV genotypes, ruling out the possibility of an assembly error or translocation event.*

Minor:

8. L72: The authors point out that the limitation of graph-based genomes is that common bioinformatic tools are not suitable for graph-based genomes. However, recent studies (e.g. https://www.nature.com/articles/s41586-022-04808-9) demonstrate the power of graph-based pangenomes and how standard tools (Kallisto etc.) can be readily applied.

*Response: Thanks for the suggestions. Although some standard tools have been applied to the graph-based tomato pangenome, the efficiency and use of these tools still heavily rely on the complexity of the pangenome graph. We still lack scalable software and mature data structures specifically for graph-based pangenome analysis. We have revised our sentence in lines 82-84 to clarify this: "scalable software and mature data structures designed explicitly for graph-based pangenome analysis are still limited at present".*

9. Fig. 5C: The red star above Hap1 is not described in the legend.

*Response: The red star indicated the position of the highest PAV signal in the pangenome in GWAS. We have included an additional description for the red star in the Fig. 5C legend.*

10. L230: Please clarify how the 1'212 bp deletion upstream of qSW5 can disrupt the coding region of GW5. In Fig. 5C, the 1'212 bp deletion is far away from the coding region of the GW5 gene.

*Response: We are sorry for the unclear writing. We have clarified our description in lines 269-272:*

*See changes: “This result is in accordance with a previous study, which demonstrated that the 950 bp deletion decreased the expression of the functional gene (qSW5), while the 1,212 bp deletion disrupts the coding region of qSW5 , leading to TGW phenotype variations”.*

*This locus contains two functional genes, namely the qSW5 and GSE5. The 1,212 bp deletion disrupts the coding region of qSW5, while the 950 bp deletion is located downstream and affects the expression of qSW5. The 370 bp deletion is located upstream of GSE5. Based on the previous study (Jiafan Liu et al. Nature Plants, 2017), GW5 acts in the brassinosteroid signalling pathway to regulate grain width and weight in rice. The mechanism controlling grain weight in this locus is complicated and is affected by different allele combinations of both genes. In the present study, we focused on the identification of functional haplotypes by GWAS, while the mechanisms underlying the qSW5 controlling the phenotypes need further investigation.*

11. L279: The sentence "Furthermore, which are common in plants." is incomplete.

*Response: We have rewritten the sentence in lines 321-323. See changes: " Furthermore, using the current graph-based pangenome, it is still difficult to detect complex structural variations such as translocations and inversions that are common in plants.*

12. L281: The authors write that it may be challenging to present the complexity of plant genomes in graph-based pangenomes. They use a citation to support this statement (ref. 30), however, the cited author (Hübner) proposes that "the graph-pangenome system can become a standard platform".

*Response: Thanks for the comment. In the cited paper, the author proposes that the graph-pangenome will become a standard platform in the future. We tried to present here that the current graph-based pangenome strategy is limited in presenting complex structural variations, which were also described in the cited paper by Hübner "Despite its compelling versatility, a graph-pangenome assembly is still challenging especially in species with large complex genomes". To clarify this, we have revised our sentence in lines 325-327: " As such, the current graph-based pangenomes may not fully represent large structural variations, which is still a great challenge for plant pangenomic studies".*

13. L432: Since the manuscript introduces a new pipeline for pangenome construction and analyses, the authors should make the code available in a public repository (GitHub) to allow reproduction of their results and/or construction of pangenomes in other species.

*Response: Thanks for the suggestion. We have released the code on GitHub (https://github.com/wjian8/psvcp_v1.01) and have attached an example dataset to help users to test and repeat the analysis in other species.*

14. Fig. A and B: The legend does not explain the difference between (A) and (B) but it appears that (B) is a zoomed-in version of (A). However, in (A) there are two regions at ~5Mbp that surpass the significance threshold of 5 -log(P) while in (B) there is only one significant region. The authors should specify in the legend the difference between (A) and (B) and explain the absence of the second significant region in (B).

*Response: Thanks for the suggestion. We have improved Fig 6a and its legend to clarify this misunderstanding. In Fig 6a, The most significant hit was detected around the 10 Mb region. Two regions (PAV markers at 3,117,040 and 4,761,060 bp) also surpass the significance threshold of 5 -log(P). Fig. 6b is a zoomed-in version of Fig. 6a, showing the regions within 3.5Mbp to 6Mbp. Therefore, in Fig. 6b, there is only one significant hit.*

Reviewer #2:

Summary of paper:

It has been well known that genomic diversity of a species cannot be presented by a single reference genome, which highlights the importance of pan-genome analyses. In this manuscript, the authors developed a pipeline (PSVCP) for a linear pan-genome construction and PAV genotype calling. They tested the pipeline by constructing a high-quality rice pan-genome using reference genomes of 12 cultivars and the pan-genome had higher contiguity and BUSCOs than the previous 3K rice pan-genome. Capturing coordinate information of PAVs in pan-genome is a novel approach and useful for downstream analysis, including PAV-based GWAS. They also compared PAV-based and SNP-based GWAS for thousand grain weight and plant height using 413 international rice accessions and the new pan-genome, which shows the strength of PAV-based GWAS over SNP-based GWAS. The manuscript is valuable for future pan-genome studies and the PSVCP pipeline is likely to be widely used for pan-genomics.

Major comments:

1. In the pan-genome construction part of PSVCP, the pipeline seems straightforward by adding new additional sequences to the reference genome from each previous step. It is unclear though how other SVs like translocations and inversions, which might have variable flanking sequences, are handled in the pipeline? The manuscript would be improved by addressing this explicitly. One suggestion would be to test in silico the effects of inversions, translocations of different sizes, regions. Are these seen as novel sequence in the resulting linear pan-genome, and how do they affect downstream mapping and variant calling?

*Response: Thanks for the reviewer's comments. Identification and display of other large SVs, such as translocations and inversions, is still challenging in pangenomics studies, even for the advanced graph-based pangenome. Therefore, the current main stream linear pangenomes approaches cannot display translocations and inversions. We have included additional content in our manuscript to discuss this limitation (lines 361-366)*

*Although it is hard to display the large SVs directly, we have attempted to improve our pipeline by including additional functions to provide the position information of large SVs (translocations and* *inversions) and genotype potential translocations and inversions at the population level using short reads. See details below:*

*We aligned novel insertions to the reference genome using BLAST+ and identified novel insertions that can further be classified as translocations. We determined the presence or absence of the translocation by looking at the read mapping around its breakpoints, examining reads spanning a 39 bp region with a conjunction point at the center. Using this method, we identified potential translocations in the pangenome. We further genotyped all translocations based on the short-read mapping results by calculating the coverage of the reads spanning the junctions (39 bp region with conjunction point in the center) of the translocation in the pangenome. We have included a further description of the translocations results in lines 160-164.*

*To demonstrate the accuracy of translocation identification, we validated the translocation genotyping results by aligning the Pacbio long read sequencing reads of two lines (IRRI2K_86 and IRRI2K_91) of the 413 accessions to the pangenome. We found the sequence (Chr11:21,534,691-21,541,116) on the pangenome has 96.267% identity to the sequence (Chr5:8,074,149-8,080,588) and these two sequences were defined as a translocation. In our translocation genotyping results, the sequence of Chr11:21534691-21541116 is absent and the Chr5:8074149-8080588 sequence is present in the IRRI2K_86. In contrast, the sequence of Chr11:21534691-21541116 is present and the Chr5:8074149-8080588 sequence is absent in the IRRI2K_91.*

*The long read alignment result showed IRRI2K_86 had the reads with a 6425 bp deletion on Chr11:21534691-21541116 and the reads spanning Chr5:8074149-8080588. IRRI2K_91 had the reads spanning Chr11:21534691-21541116 and the reads with 6441 bp deletion on Chr5:8074149-8080588. We further validated the translocations genotyping results by comparing the genomes of accession IRRI2K_86 and IRRI2K_91 with the pangenome using mummer. The result showed IRRI2K_86 had a 6425 deletion on Chr11:19375935 and IRRI2K_91 had a 6441 bp deletion on Chr5: 7144687. The result demonstrates the accuracy of translocation detection and genotyping in our pipeline, which is summarized in Fig S6-S11 and Tables S5-S7.*

*For inversions, we identified the inversions by comparing each genome used for pangenome construction with the pangenome using Assemblitics. We further genotyped each potential inversion in the pangenome from 413 accessions by examining the mapping coverage of the 39 bp region with breakpoints in the centre. We further validate the inversion genotyping results by comparing the genomes of accession IRRI2K_86 and IRRI2K_91 with the pangenome using mummer. We found an inversion on Chr5:13924048-13925592. In our inversion genotyping results, IRRI2K_91 had the inversion in regions of Chr5:13924048-13925592 compared to our pangenome and IRRI2K_86 did not contain the inversion, which was syntenic with our pangenome in regions of Chr5:13924048-13925592. The Pacbio long read alignment result showed IRRI2K_86 had long reads spanning 39 bp region with conjunction point (Chr5:13924048 conjunction point and Chr5: 13925592 conjunction point) in the center. But IRRI2K_91 did not have the long reads spanning the 39 bp region with conjunction point (Chr5:13924048 conjunction point and Chr5: 13925592 conjunction point) in the center. We further validated the inversion genotyping results by comparing the genomes of accession IRRI2K_86 and IRRI2K_91 with the pangenome using mummer. The results showed the sequence Chr5: 12329090-12407319 on IRRI2K_86 is consistent with the sequence Chr5: 13902408- 13980631 on the pangenome. Therefore, this indicates the sequence Chr5: 12391459-12393001 on IRRI2K_91 is an inversion of the sequence Chr5:13924048- 13925592 on the pangenome. The result is summarized in Fig S12-S17 and Table S8-S11.*

*Since identifying and genotyping translocations and inversions is still challenging using short-read sequencing, we carefully described the identified SVs as potential inversions and translocations and included additional sentences to inform the limitations of short-reading sequencing for identifying translocation and inversion in lines 361-366 and our GitHub page.*

*See changes: “PSVCP is robust in placing novel PAV sequences into the linear pangenome, it may be limited to display more complex SVs such as translocations and inversions, which is a challenge in current pangenomics studies, even for the advanced graph-based pangenome. In addition, due to its read length, short-read sequencing data may have lower sensitivity for SVs detection compared with long-read sequencing data.”*

2. It would be great to have a list and details of the 413 rice accessions as a supplementary table and also to release the PAV matrix.

*Response: We have included the details of the 413 rice accessions in Supplementary Table S24. The constructed rice pangenome, the annotated files and the PAV matrix now have been uploaded to a public database (https://osf.io/38gtp/).*

3. Figure legends and methods sections require more details in general.

*Response: Thanks for the comment. We have improved the descriptions in the figure legends and included further details in the method sections.*

Minor comments:

4. Any specific reasons to include only the 413 accessions re-sequenced lines rather than the >3000 that are available? As it currently stands the paper is largely about methods but were a larger selection of genotypes used it could be elevated to provide a major resource for the rice genomics community. It seems relatively straightforward for the authors to run the pipeline on a complete dataset of short read data across rice and provide the resulting PAV matrix as a supplemental dataset, even without changing the core of the paper focusing on the 413 lines they resequenced.

*Response: Thanks for the comments. The accessions from this study were selected from the RPD panel (McCouch SR et al. Open access resources for genome-wide association mapping in rice. Nature Communications, 2016) based on diversity and representativeness calculated using the whole genome genotypes. The RPD panel contains about 2000 accessions and is a representative collection from worldwide (96 countries). This panel was selected alongside the 3K panel (Wang et al. Genomic variation in 3,010 diverse accessions of Asian cultivated rice. Nature. 2018) parallel by the International Rice Research Institute. Both panels are representative in terms of the genetic diversity of worldwide Asian rice. Furthermore, in this study, we sequenced in a higher coverage (>50X) than the 3K data set (~15X), which can result in more accurate SVs identification and characterization. To make it more clear, we have provided further information about sample selection in lines 477-481.*

*We have also followed the reviewer's comment by generating an additional PAV matrix using 3k data (>15X) and this data is now provided as supplementary data, which can be accessed via https://osf.io/38gtp/. The sequencing data used to generate the additional PAV matrix is summarized in Table S4.*

5. There seems to be a missed opportunity to test selection on PAV. The dataset the authors generated seems powerful to address various hypotheses. The authors could, for example, ask whether genic PAV have lower minor allele freq (purifying selection) than intergenic PAV.

*Response: Thanks for the suggestions. We have performed an additional analysis based on our PAV matrix to detect the minor allele frequency between genic PAV and intergenic PAV. The result shows that genic PAV regions have a slightly higher minor allele frequency than intergenic PAV regions. This result is displayed in Fig S22 and Table S17, and included in lines 185-188.*

*See changes ”We further analysed the minor allele frequency (MAF) of PAVs within genic and intergenic regions. A higher MAF was observed within genic regions (Additional file 1: Fig. S22 and Additional file 2: Table S17), suggesting a stronger purifying selection for PAVs in genic regions.”*

6. Pg 2, Line 60, "efficiently" should be "efficient".

*Response: Thanks for the careful review. We have changed it as suggested in Line 62.*

7. Pg 3, Line 101, "level based" could be just "level".

*Response: Thanks for the careful review. We have changed it as suggested in line 114.*

8. Pg 3, Line 101, remove "'" at the end of accessions'

*Response: Thanks for the suggestions. We have changed it as suggested in line 112.*

9. Pg 3. Lines 111-114, It is confusing where 35.7 % came from. It would be helpful to make it clear if 35.7% is the percentage out of 43.1% or out of the total 100%. In addition, 6,797 sequences were fully nested in 5,925 Nipponbare genes? That means ~27.6% sequences out of 100% (24,585 sequences) were completely resided in the genes?

*Response: 35.7% is the percentage out of the total of 100%. We clarify this in lines 132-133. See changes “while 35.7% of the additional PAV sequences overlapped with genic regions”.*

*We analysed the distribution of these additional PAV sequences. We found that 43.1% overlapped ±2 kb upstream/downstream of genes. In comparison, 35.7% of the additional PAV sequences overlapped with genic regions (Fig. S2c). 6,797 sequences were fully nested in 5,925 Nipponbare genes, indicating ~27.6% of sequences out of 100% (24,585 sequences) completely resided in the Nipponbare genes. We found that 1,939 new genes de novo annotated in other genomes had an intersection with 1,988 PAV sequences. The percentage of PAV sequences overlapped with genic regions was 35.7% ((6,797 + 1,988) / 24,585).*

10. Pg 5, Line 231, "accord" should be "accordance".

*Response: Thanks for the suggestions. We have changed it as suggested in line 269.*

11. Pg 7, Line 279, "Furthermore, which are common in plants" should be rewritten.

*Response: We have rewritten the sentence in Lines 321-323. See changes: " Furthermore, using the current graph-based pangenome, it is still difficult to detect complex structural variations such as translocations and inversions that are common in plants.”*

12. Pg 8, Line 350, "The phylogeny variations" could be rephrased as "The topological variation between SNP- and PAV-based phylogenetic analyses".

*Response: Thanks for the suggestions. We have changed it as suggested in line 402.*

13. Pg 10, Line 434, Is MSU same as NIP because I do not see MSU in Table S1, but NIP instead.

*Response: Thanks for the suggestions. NIP represents the Nipponbare cultivar, which is the accession used for rice genome assembly. MSU is the reference genome assembled and annotated for Nipponbare. We have kept these terms consistent in the revised manuscript.*

14. Pg 10, Line 437, I found that the acronym "NIP" was first mentioned here, so it should be clarified what this means here. "Nipponbare (NIP)"

*Response: Thanks for the suggestions. We have changed it as suggested in Line 256.*

15. In Figure 4, The color of lines should be labeled in Fst Analysis.

In the legend of Figure 4, "datga" needs to be changed to "data"?

*Response: Thanks for the suggestions. We have changed it as suggested.*

16. The authors should cite: https://www.nature.com/articles/s41422-022-00685-z

*Response: Thanks for the suggestions. We have cited this paper as suggested in lines 77-80.*

**Second round of review**

**Reviewer 1**

I appreciate the authors' efforts to adequately address all my previous comments and concerns on the original manuscript.

**Reviewer 2**

The authors have largely addressed the previous comments and the manuscript is definitely improved.

Remaining comments:

Regarding the test of selection on PAV based on allele frequencies. I am confused by the authors interpretation. The authors show that genic PAV are at higher minor allele frequency, which is the opposite from what one would expect if they are subject to stronger purifying selection, but conclude the opposite. It may be important for the authors to comment on limits to MAF distributions when using only a subset of genome assemblies.

**Authors Response**

**Point-by-point responses to the reviewers’ comments:**

Reviewer #1:

I appreciate the authors' efforts to adequately address all my previous comments and concerns on the original manuscript.

*Response: Thanks for your constructive comments and we appreciate your help in improving the overall quality of the manuscript.*

Reviewer #2:

The authors have largely addressed the previous comments and the manuscript is definitely improved.

Remaining comments:

Regarding the test of selection on PAV based on allele frequencies. I am confused by the authors interpretation. The authors show that genic PAV are at higher minor allele frequency, which is the opposite from what one would expect if they are subject to stronger purifying selection, but conclude the opposite. It may be important for the authors to comment on limits to MAF distributions when using only a subset of genome assemblies.

*Response: Thanks to the reviewer for pointing out our inaccurate interpretation of the results. We agreed that higher purifying selection could lead to a reduction in genetic diversity and a lower MAF. We also agreed with the reviewer that the number and diversity of sequencing data could lead to variations in the PAV matrix and affect the measurement of MAF distributions.*

*We have amended our interpretation of the relationship between MAF and the purifying selection and included a caveat on using a subset of sequencing data to calculate the MAF as follow in the revised manuscript:*

*"We further analysed the minor allele frequency (MAF) of PAVs within genic and intergenic regions. A higher MAF was observed within genic regions (Additional file 1: Fig. S22 and Additional file 2: Table S17), suggesting a lower purifying selection for PAVs in genic regions. It is noteworthy that although our analysis was based on a representative subset of an international diverse panel (the selected 413 accessions), analysis using this subset may not fully display the MAF distribution of all rice germplasms.”*
